# Stereoselective Substituted Oxasilinane Synthesis from Diallylsilanes via Electrophile-Promoted Rearrangement, RCM, and Re-Catalyzed Allylic Transposition

**DOI:** 10.1021/acs.joc.6c00566

**Published:** 2026-07-24

**Authors:** Gregory W. O’Neil, Inna A. Fomina, Christopher R. Myers, Zachary T. Rademacher, Rawley M. Spencer, Robert F. Berger

**Affiliations:** Department of Chemistry, 1632 Western Washington University, Bellingham, Washington 98229, United States

## Abstract

A new synthesis of substituted oxasilinanes is described, starting from diallylsilanes and featuring a diastereoselective rhenium­(VII) oxide-catalyzed allylic transposition of an intermediate 8-membered ring cyclic silyl ether. The results obtained suggest reversibility for the allylic transposition reaction, evidenced by time-dependent diastereoselectivity and formation of the more thermodynamically stable *cis-*substitued oxasilinane product.

## Graphical abstract

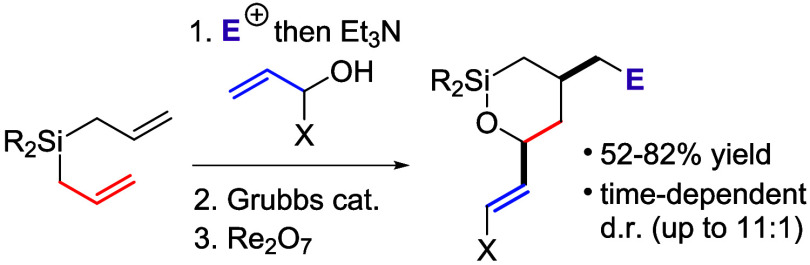

## Introduction

Our group has had an interest in synthesizing ring systems containing both silicon and oxygen.^1–6^ These compounds can serve as useful synthetic intermediates, for instance with silicon “protecting” the oxygen atom,^7^ allowing for further transformations that would otherwise be sensitive to the presence of a mildly acidic hydroxyl group. Oxidative desilylation can introduce additional hydroxyl groups,^8^ leading to useful polyol products. Alternatively, silicon may be retained to produce valuable polymeric materials^9^ or small molecules that may be of interest to medicinal chemists.^10,11^

One method we have utilized for synthesizing silicon and oxygen-containing rings begins from polyallylsilanes and employs a sequence of iodine-promoted rearrangement followed by ring-closing metathesis (RCM) with Grubbs first- (**Ru–I**) or second generation catalysts (**Ru–II**).^3,4^ In this way, we have been able to prepare a variety of oxasilacycles (Scheme 1). Other reports of oxasilacycle formation by RCM can be found, with the resulting products proving to be useful intermediates in synthesis.^12–18^ For example, Volchkov et al. reported the use of RCM product 8-membered oxasilacycles in subsequent rhenium-catalyzed 1,3-allylic transposition reactions to form ring-contracted oxasilinanes.^19^ This methodology then enabled an efficient synthesis of the anticancer natural product amphidinolide V.^20^ We questioned if the 8-membered oxasilacycles we had synthesized via diallylsilane rearrangement/RCM might also undergo Re-catalyzed allylic transposition.^21–25^

**Scheme 1:**
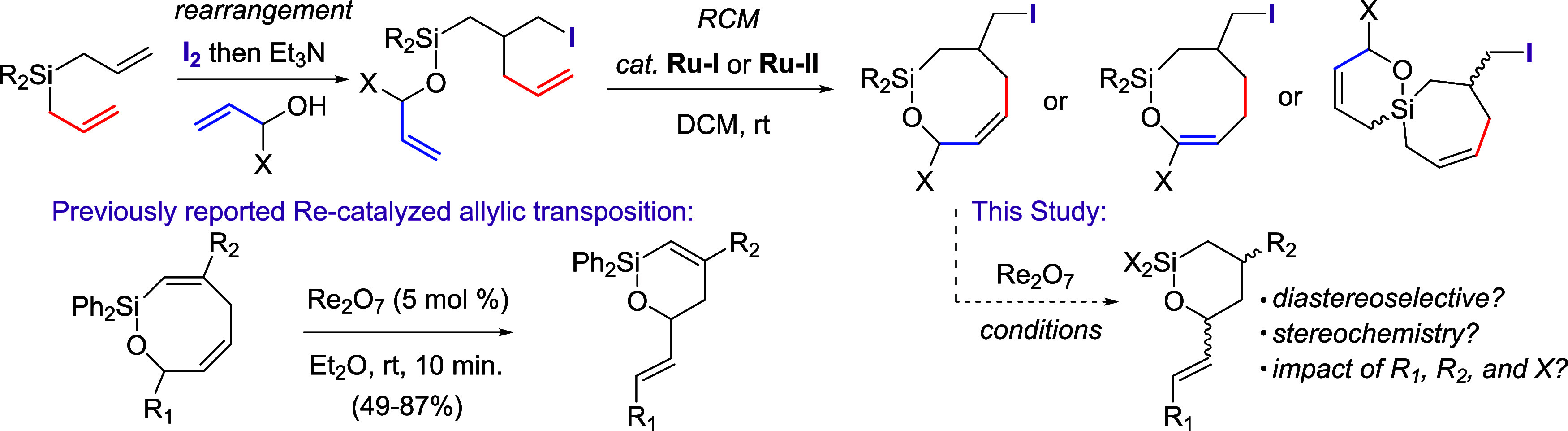
Oxasilinane Synthesis by Combining Diallylsilane Rearrangement/RCM Methodology with Re-Catalyzed Allylic Transposition of 8-Membered Oxasilacycles

## Results and Discussion

As an initial test, an ether solution of compound **1** ^3^ was treated with Re_2_O_7_ for 10 min at room temperature according to Volchkov’s method (Scheme 2).^19^ ^1^H NMR analysis of the reaction mixture showed a small amount of the expected product **2** (∼30% conversion) along with primarily unreacted starting material. Extending the reaction time to 90 min resulted in complete conversion, producing **2** in 68% isolated yield. Notably, not only the conversion, but also the diastereomeric ratio (d.r.) of **2** was different than what was observed from the 10 min/30% conversion reaction. More specifically, at 10 min the d.r. of **2** was ∼1.3:1 but increased to 8.5:1 after 90 min.

**Scheme 2:**
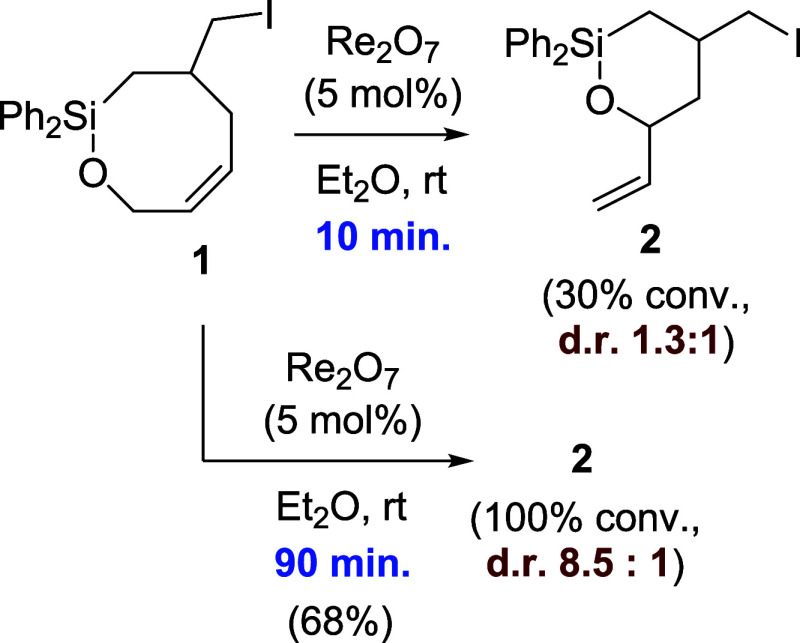
Re-Catalyzed Allylic Transposition of **1** and Time-Dependent Diastereoselectivity

To investigate this further, reactions of **1** with Re_2_O_7_ were performed and quenched at several additional time intervals. As shown in Table 1, the d.r. steadily increases over time. For example, the d.r. was 1.7:1 at 15 min, 2.8:1 at 30 min, and 4.7:1 at 1 h before reaching a maximum of 11:1 after 3 h.

**Table 1:**
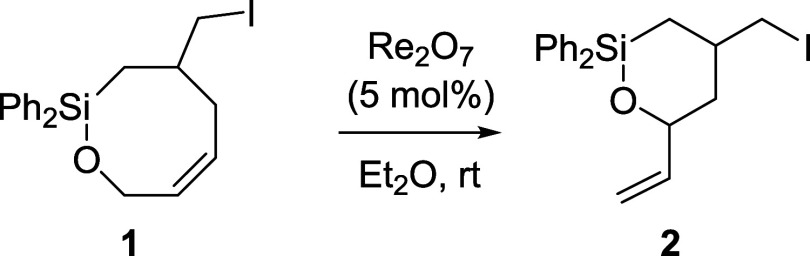
Results from Re-Catalyzed Conversion of **1** into **2** at Different Time Increments^c^

time	d.r.^a^	%conversion^a^ (yield^b^)
15 min	1.7:1	67
30 min	2.8:1	100
1 h	4.7:1	100
3 h	10.9:1	100 (80)
6 h	11.0:1	100

a From ^1^H NMR analysis.

b Isolated yield from reaction performed at 0.5 g scale.

c Reactions were performed by adding Re_2_O_7_ (5 mol %) to an ether solution of **1** (0.1 g) at room temperature and quenching at the times indicated by filtering through silica gel.

One explanation is that **2**, being an allylic ether, can undergo equilibration by reacting further with Re_2_O_7_ (Scheme 3). This would be consistent with Volchkov’s report, who noted some racemization in their reactions when using enantioenriched starting materials, and the extent of racemization was time dependent.^19^ The authors suggested a competing cationic mechanism (as opposed to a concerted [3,3]-sigmatropic rearrangement) via intermediate **Re–I** to explain the observed loss in enantiopurity. Based on our results, reversibility in oxasilinane formation from intermediate **Re–I** might also contribute to racemization in their system. NMR analysis of **2** indicated that the major diastereomer has both the allyl- and iodomethyl groups oriented equatorial about the oxasilinane ring,^26,27^ consistent with an equilibration process that would favor the more thermodynamically stable compound. As a further test, compound **2** was isolated as a 1.8:1 mixture following an allylic transposition reaction that proceeded with partial conversion (*ref*. Table 1). Resubjection of this 1.8:1 diastereomeric mixture to Re_2_O_7_ for 6 h produced **2** as an 11:1 mixture of diastereomers in 83% yield (see Supporting Information, Figure S3).

**Scheme 3:**
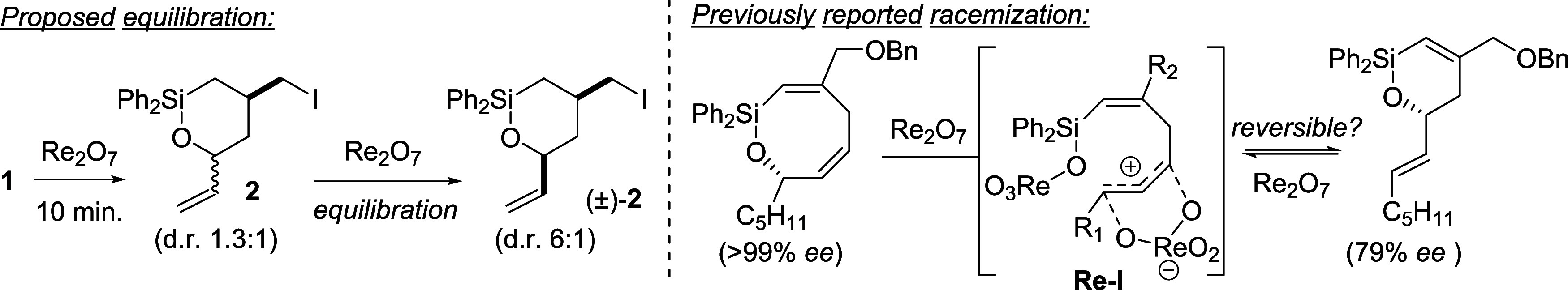
Proposed Equilibration Mechanism to Explain Time-Dependent Diastereoselectivity (Left) and Racemization^19^ (Right) during Re­(VII) Oxide Catalyzed Allylic Transpositions of 8-Membered Ring Silyl Ethers^a^

a The structure of **Re–I** is adapted from the report of Volchkov et al.,^19^ where dashed lines are understood to represent partial bonds with some loss of stereochemical information.

To confirm that the major diastereomer obtained is the thermodynamic product, DFT calculations were performed. In addition to calculating the energy difference between the different diastereomers of **2**, calculations were also performed on the analogous dimethylsilyl **3** as well as diphenyl- and dimethylsilyl **4** and **5** containing a methyl substituent at the 4-position in anticipation that the substituents about the oxasilinane ring would affect the energy difference between the different diastereomers (Table 2). Compounds **2** and **4** containing larger phenyl substituents on silicon resulted in a larger preference for the *cis*-diastereomer, with both groups at the 4- and 6-position in an equatorial orientation compared to their dimethyl analogs (ΔΔ*G* for **2** vs **3** = 1.8 kJ/mol and **4** vs **5** = 1.6 kJ/mol). Whether the substituent at the 4-position is iodomethyl or methyl had little affect on the energetic preference (ΔΔ*G* within 0.1 kJ/mol).

**Table 2:**
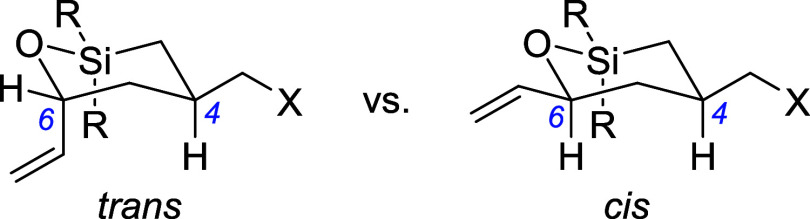
DFT Conformational Analysis of 4,6-Substituted Oxasilinanes

compound	R	X	Δ_*trans–cis*_ (kJ·mol^–1^)
**2**	Ph	I	7.94
**3**	Me	I	6.14
**4**	Ph	H	7.86
**5**	Me	H	6.22

To further investigate the results from our DFT calculations, we attempted to synthesize compounds **3**-**5** via Re-catalyzed allylic transposition. To begin, the iodine-promoted rearrangement of diallyldimethylsilane was performed followed by etherification with allyl alcohol, affording compound **6** in 69% yield (Scheme 4). RCM using **Ru–I** proceeded smoothly to deliver the somewhat unstable 8-membered oxasilacyle **7** en route to compound **3**. Acid-promoted rearrangement of diallyldiphenylsilane according to Suslova’s conditions afforded fluorosilane **8**.^28,29^ In line with Suslova’s report,^29^ attempted rearrangement of diallyldimethylsilane under these conditions failed, which would have led to oxasilacycle **5**. Nonetheless, we were able to further pursue the synthesis of another target oxasilacycle **4** by substitution of the fluorine substituent in **8** with the potassium alkoxide of allyl alcohol followed by RCM with **Ru–I** which gave the 8-membered oxasilacyle **10**.

**Scheme 4:**
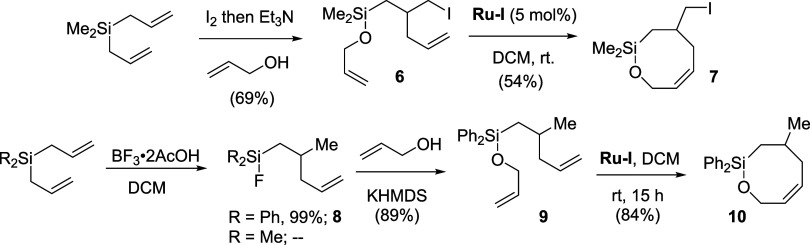
Synthesis of 8-Membered Ring Oxasilacycles **7** and **10** from Diallyldimethyl- and Diallyldiphenylsilane

Treatment of **7** and **10** with Re_2_O_7_ produced the expected oxasilinanes **3** and **4** respectively in comparably good yield (Table 3). In all cases, the *cis*-diastereomer was the major stereoisomer based on ^1^H NMR analysis.^26^ Consistent with our DFT results, a higher d.r. was observed for the diphenylsilyl compounds **2** and **4**. Also consistent was the similar d.r. measured for **2** bearing an iodomethyl substituent compared to **4** with a smaller methyl group at this position (ΔΔ*G* = 0.08 kJ/mol by DFT). The lower yield of dimethylsilyl **3** likely reflects its lower stability than diphenylsilyl compounds **2** and **4**, in line with what was observed when synthesizing the 8-membered ring oxasilacycle precursor **7**. This might also explain why Volchkov and co-workers only included diphenylsilyl ethers in their Re-catalyzed allylic transpositions.^19^ Conversion in all cases was 100%. Decomposition and/or byproduct pathways may also therefore explain the lower d.r. values observed compared to what would be expected from DFT calculations (*ref*. Table 2).

**Table 3:**
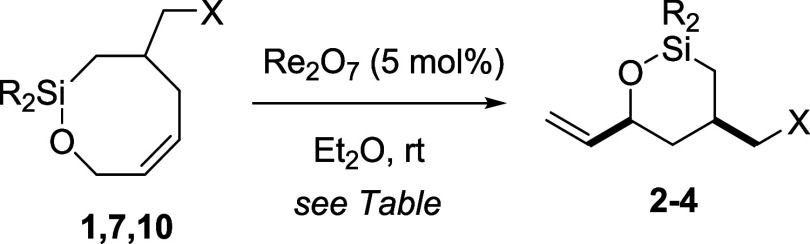
Results from Re-Catalyzed Allylic Tranposition of 8-Membered Ring Silyl Ethers^d^

compound	yield^a^	d.r.^b^	DFT^c^
R = Ph, X = I; **2**	82%	10:1	96:4
R = Me, X = I; **3**	52%	6:1	92:8
R = Ph, X = H; **4**	67%	9:1	96:4

a Isolated Yield.

b From ^1^H NMR analysis.

c Based on computed energy differences.

d Reactions were performed by adding Re_2_O_7_ (5 mol %) to an ether solution of the 8-membered oxasilolane at room temperature and stirring for 3 h.

Higher yields were reported by Volchkov et al. for transposition reactions resulting in oxasilinane products containing disubstituted alkenes (as opposed to terminal double bonds).^19^ To see if that same trend also applied to our system, 8-membered oxasilacycles **13** and **14** were prepared from diallyldiphenylsilane via intermediates **11** and **12** respectively as outlined in Scheme 5. Treatment of **13** and **14** with catalytic Re_2_O_7_ generated silinanes **15** and **16** in comparable yield and diastereoselectivity, not only to each other but also to oxasilinane **2** suggesting that double bond substitution in in this case does not have a major influence on the reaction outcome. Since **13** and **14** were used as a ∼1:1 mixture of diastereomers and produced oxasilinane products **15** and **16** selectively as the thermodynamically preferred *cis,E*-stereoisomer, this further supports a mechanism involving equilibration and cationic intermediate **Re–II**.

**Scheme 5:**
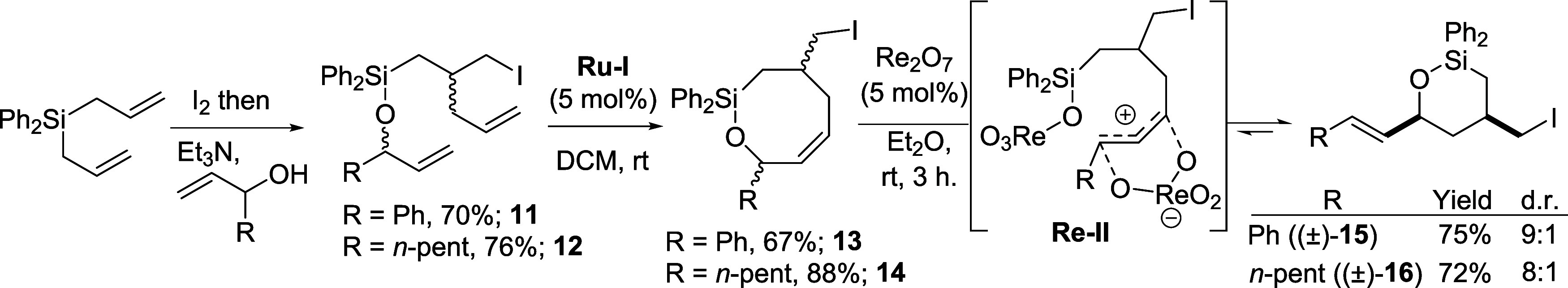
Synthesis of Double Bond-Substitued Oxasilinanes **15** and **16**

To demonstrate the sythetic utility of this transformation, a three-step sequence was developed starting from oxasilinane **2** taking advantage of the different functional groups present (Scheme 6). More specifically, substitution of the alkyl iodide in **2** with sodium benzenesulfinic acid produced phenyl sulfone **17** in 83% yield, setting the stage for further functionalizations such as a Julia olefination.^30,31^ Tamao-Fleming oxidation^32^ of **17** proceeded smoothly to deliver the desilylated diol **18** in 78% yield. Finally, cross-metathesis^33^ with diacetoxy-2-butene gave allylic acetate **19**, introducing another functional group capable of further C–C bond forming reactions.^34–36^

**Scheme 6:**
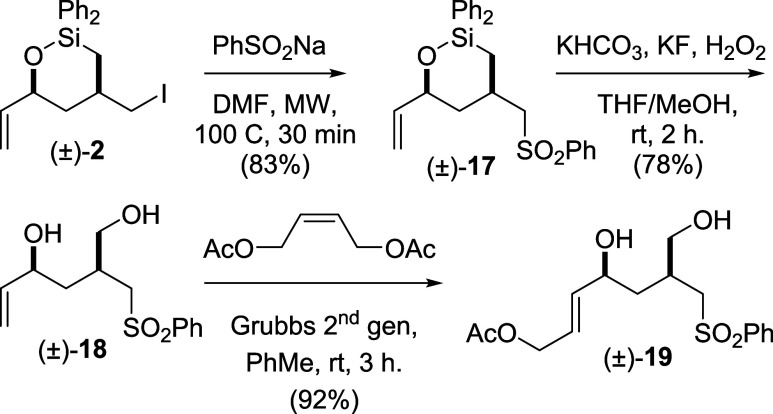
Further Functionalization of Re-Catalyzed Allylic Transposition Product **2**

## Conclusion

In summary, a series of substituted oxasilinanes were stereoselectively synthesized from diallylsilanes beginning with iodine- or proton-mediated rearrangement, followed by RCM with Grubbs’ first-generation catalyst, and finally Re-catalyzed allylic transposition. Monitoring reaction progress by ^1^H NMR revealed that diastereoselectivity steadily increased to a maximum after approximately 3 h. Combined with formation of the more thermodynamically favorable oxasilinane, supported by DFT calculations, this was interpreted as evidence for the allylic transposition being reversible at room temperature. Further evidence was obtained by resubjecting a 1.8:1 mixture of oxasilinane diastereomers to Re_2_O_7_, which allowed for recovery of the oxasilinane in 83% yield now as an 11:1 diasteromeric mixture.

Using this sequence several *cis*-substituted oxasilinanes were prepared representing potentially valuable synthetic intermediates. For instance, oxasilinanes **2**, **3**, **15**, and **16** feature C–C double bonds, a primary alkyl iodide, and O–Si/C-Si bonds, each representing sites for further functionalization. As a demonstration of their synthetic utility, oxasilinane **2** was taken through a high-yielding three-step sequence involving substitution of the alkyl iodide, Tamao oxidation, and cross-metathesis. In this way, a highly functionalized diol was produced featuring an allylic acetate and phenyl sulfone for C–C bond formation. Efforts are ongoing to further investigate this series of reactions in the context of complex target-oriented synthesis.

## Experimental Section

### General Information

All reactions were carried out in vessels under an argon atmosphere unless otherwise specified. Reactions that required heating were conducted on a heating stir plate in heating blocks unless otherwise specified. Dry solvents were prepared by passing the solvent through a column of activated alumina under nitrogen immediately prior to use. All reagents were purchased and used as received unless mentioned otherwise. TLC analysis used 0.25 mm silica layer fluorescence UV_254_ plates. Column chromatography: silica gel (230–400 mesh). IR: FT-IR with single-bounce diamond ATR. NMR: Spectra were recorded on a 500 MHz spectrometer in CDCl_3_ or C_6_D_6_; chemical shifts (δ) are given in ppm, coupling constants (*J*) in Hz. Solvent signals were used as references (CDCl_3_: δ 77.0 ppm; residual CHCl_3_ in CDCl_3_: δ 7.26 ppm, C_6_D_6_: δ 128.06 ppm; residual C_6_H_6_ in C_6_D_6_ δ 7.16 ppm). HRMS: quadrupole time-of-flight LC-MS with electrospray ionization (ESI positive and negative). MW: CEM Discover 2.0 Microwave Synthesizer. Structural assignments were made with additional information from gCOSY, gHSQC, and gHMBC experiments.

### General Procedure A: Re-catalyzed Allylic Transposition

With slight modification to the procedure reported by Volchkov,^20^ to a solution of the 8-membered ring silyl ether (1.0 mmol, 1.0 equiv) in dry diethyl ether (to make a 0.3 M solution) was added Re_2_O_7_ (0.05 mmol, 0.05 equiv) and the mixture was stirred for 3 h. The mixture was then filtered through a plug of silica with MTBE and concentrated on a rotary evaporator. Purification by flash chromatography on silica (10:1 hexanes:methyl *tert*-butyl ether) afforded the oxasilinane as an oil.

### General Procedure B: Iodine-Mediated Rearrangement/Etherification of Diallylsilanes

To a solution of the diallylsilane (1.5 mmol, 1.0 equiv) in DCM (to make a 0.1 M solution) at rt was added I_2_ (1.5 mmol, 1.0 equiv) and the mixture was stirred for 3 h. The reaction was cooled to 0 °C before adding triethylamine (4.5 mmol, 3.0 equiv) followed by the alcohol (2.25 mmol, 1.5 equiv). The solution was allowed to slowly warm to rt over 3 h and stirred at this temperature for another 1–2 h (until the reaction was complete by TLC). Upon completion, water was added and the mixture was extracted with DCM. The combined organic extracts were dried over MgSO_4_, filtered, and concentrated *in vacuo*. Purification by flash chromatography on silica (10:1 hexanes:methyl *tert*-butyl ether) gave the corresponding silyl ether as a colorless oil.

### General Procedure C: Oxasilacycle Formation by Ring-Closing Metathesis

To a solution of the dienylsilyl ether (1.0 mmol, 1.0 equiv) in degassed DCM (to make a 0.05 M solution) at rt was added Grubbs first- (**Ru–I**) or second-generation (**Ru–II**) catalyst (0.05 mmol, 0.05 equiv). The mixture was stirred for 15 h before concentrating *in vacuo* and purifying by flash chromatography on silica (2:1 hexanes:dichloromethane).

#### (*Z*)-4-(Iodomethyl)-2,2-diphenyl-3,4,5,6-tetrahydro-1,2-oxasilocine (**1**)

Following general procedure C using (allyloxy)­(2-(iodomethyl)­pent-4-en-1-yl)­diphenylsilane^3^ (3.0 g, 6.7 mmol) and **Ru–I** (0.275 g, 0.33 mmol). Purification by flash chromatography on silica (2:1 hexanes:dichloromethane) gave **1** (2.1 g, 74%) as a colorless oil. FTIR (ATR): 2955, 2928, 2865, 1450, 1370, 1310, 1235, 1121, 1065, 978, 884, 755, 699 cm^–1^. ^1^H NMR (500 MHz, CDCl_3_) δ 7.62–7.59 (m, 2H), 7.56–7.53 (m, 2H), 7.43–7.38 (m, 6H), 5.79 (dt, *J* = 11.2, 4.7 Hz, 1H), 5.60 (dddt, *J* = 11.3, 9.8, 8.2, 1.6 Hz, 1H), 4.55 (dd, *J* = 15.5, 4.5 Hz, 1H), 4.48 (dd, *J* = 15.6, 5.2 Hz, 1H), 3.18 (d, *J* = 7.7 Hz, 2H), 2.84 (ddd, *J* = 13.7, 9.6, 4.3 Hz, 1H), 2.39 (ddd, *J* = 13.5, 8.3, 5.5 Hz, 1H), 2.17 (ddtd, *J* = 11.3, 9.7, 7.1, 4.3 Hz, 1H), 1.58 (ddd, *J* = 14.9, 4.3, 1.0 Hz, 1H), 1.23 (dd, *J* = 14.9, 10.9 Hz, 1H). ^13^C­{^1^H} NMR (126 MHz, CDCl_3_) δ 135.6, 134.8, 134.11, 134.06, 131.8, 129.93, 129.87, 127.97, 127.95, 127.3, 62.2, 37.4, 31.8, 19.4, 16.9. HRMS-TOF (ESI/QTOF) *m*/*z*: [M + Na]^+^ Calcd for C_19_H_21_INaOSi^+^ 443.0299. Found 443.0300.

#### 4-(Iodomethyl)-2,2-diphenyl-6-vinyl-1,2-oxasilinane (**2**)

Prepared according to the general procedure A using **1** (0.5 g, 1.2 mmol). Purification by flash chromatography on silica (10:1 hexanes:methyl *tert*-butyl ether) gave **2** (0.40 g, 80%) as a colorless oil and a 11:1 mixture of diastereomers. FTIR (ATR): 2955, 2928, 2865, 1450, 1370, 1310, 1235, 1121, 1065, 978, 884, 755, 699 cm^–1^. *Spectral data for the major diastereomer:* ^1^H NMR (500 MHz, CDCl_3_) δ 7.67 (dd, *J* = 7.7, 1.7 Hz, 2H), 7.57 (dd, *J* = 8.0, 1.5 Hz, 2H), 7.48–7.43 (m, 2H), 7.42–7.38 (m, 2H), 7.37–7.32 (m, 2H), 5.97 (ddd, *J* = 17.1, 10.4, 5.0 Hz, 1H), 5.39 (d, *J* = 17.1 Hz, 1H), 5.13 (d, *J* = 10.4 Hz, 1H), 4.54–4.34 (m, 1H), 3.27 (dd, *J* = 9.6, 5.4 Hz, 1H), 3.17 (dd, *J* = 9.7, 6.9 Hz, 1H), 2.08–1.94 (m, 2H), 1.62–1.45 (m, 1H), 1.27 (dt, *J* = 13.3, 11.3 Hz, 1H), 0.81 (dd, *J* = 14.4, 13.0 Hz, 1H). ^13^C­{^1^H} NMR (126 MHz, CDCl_3_) δ 140.4, 134.9, 134.30, 134.27, 134.2, 130.23, 130.15, 128.2, 127.9, 113.8, 74.6, 42.3, 36.9, 18.8, 18.4. HRMS (ESI/QTOF) *m*/*z*: [M + H]^+^ Calcd for C_19_H_22_IOSi^+^ 421.0479. Found 421.0475.

#### 4-(Iodomethyl)-2,2-dimethyl-6-vinyl-1,2-oxasilinane (**3**)

Prepared according to the general procedure A using **7** (0.2 g, 0.84 mmol). Purification by flash chromatography on silica (10:1 hexanes:methyl *tert*-butyl ether) gave **3** (0.13 g, 52%) as a colorless oil and a 6:1 mixture of diastereomers. FTIR (ATR): 3069, 2968, 1448, 1374, 1188, 1157, 1072, 1050, 972, 913, 858, 770 cm^–1^. *Spectral data for the major diastereomer:* ^1^H NMR (500 MHz, CDCl_3_) δ 5.87 (ddd, *J* = 17.1, 10.4, 5.4 Hz, 1H), 5.27 (dt, *J* = 17.1, 1.6 Hz, 1H), 5.08 (dt, *J* = 10.4, 1.5 Hz, 1H), 4.26 (ddd, *J* = 11.2, 5.4, 1.7 Hz, 1H), 3.21 (dd, *J* = 9.6, 5.2 Hz, 1H), 3.09 (dd, *J* = 9.6, 7.2 Hz, 1H), 1.99–1.91 (m, 1H), 1.91–1.85 (m, 1H), 1.05 (dt, *J* = 13.3, 11.4 Hz, 1H), 0.97–0.81 (m, 1H), 0.35 (t, *J* = 13.4 Hz, 1H), 0.22 (s, 3H), 0.18 (s, 3H). ^13^C­{^1^H} NMR (126 MHz, CDCl_3_) δ 140.7, 113.8, 77.3, 76.7, 74.2, 42.1, 37.0, 20.9, 19.2, −0.5, −2.2. HRMS (ESI/QTOF) *m*/*z*: [M + H]^+^ Calcd for C_9_H_18_IOSi^+^ 297.0166. Found 297.0159.

#### 4-Methyl-2,2-diphenyl-6-vinyl-1,2-oxasilinane (**4**)

Prepared according to the general procedure A using **10** (0.18 g, 0.61 mmol). Purification by flash chromatography on silica (10:1 hexanes:methyl *tert*-butyl ether) gave **4** (0.12 g, 67%) as a colorless oil and a 9:1 mixture of diastereomers. FTIR (ATR): 2964, 2923, 2856, 1494, 1457, 1429, 1375, 1116, 1095, 1069, 1028, 946, 865 cm^–1^. *Spectral data for the major diastereomer:* ^1^H NMR (500 MHz, CDCl_3_) δ 7.71–7.63 (m, 2H), 7.59–7.52 (m, 2H), 7.45–7.40 (m, 2H), 7.40–7.31 (m, 4H), 5.95 (ddd, *J* = 17.1, 10.4, 5.1 Hz, 1H), 5.34 (dt, *J* = 17.1, 1.7 Hz, 1H), 5.08 (dt, *J* = 10.5, 1.7 Hz, 1H), 4.43 (ddq, *J* = 11.2, 5.2, 1.7 Hz, 1H), 1.94 (dddq, *J* = 15.9, 9.7, 6.5, 3.2 Hz, 1H), 1.70 (dq, *J* = 13.5, 2.3 Hz, 1H), 1.33 (ddd, *J* = 14.6, 3.4, 2.1 Hz, 1H), 1.32–1.20 (m, 1H), 1.08 (d, *J* = 6.5 Hz, 3H), 0.75 (dd, *J* = 14.7, 12.9 Hz, 1H). ^13^C­{^1^H} NMR (126 MHz, CDCl_3_) δ 141.2, 134.34, 134.33, 134.25, 134.0, 129.9, 129.8, 128.0, 127.83, 127.77, 114.1, 113.2, 75.2, 73.1, 44.3, 41.8, 29.5, 27.1, 19.9, 0.0. HRMS (ESI/QTOF) *m*/*z*: [M + H]^+^ Calcd for C_19_H_23_OSi 295.1513. Found 295.1509.

#### (Allyloxy)-(2-(iodomethyl)­pent-4-en-1-yl)­dimethylsilane (**6**)

Prepared according to general procedure B using diallylmethylsilane (0.28 mL, 1.54 mmol). Purification by flash chromatography on silica (10:1 hexanes:methyl *tert*-butyl ether) gave **6** (0.35 g, 69%) as a colorless oil. FTIR (ATR): 3068, 2972, 2951, 1410, 1353,, 1109, 1021, 997, 914, 877, 732 cm^–1^; ^1^H NMR (500 MHz, CDCl_3_) δ 5.92 (ddt, *J* = 17.1, 10.5, 4.8 Hz, 1H), 5.70 (ddt, *J* = 17.2, 10.1, 7.2 Hz, 1H), 5.26 (dd, *J* = 17.1, 1.8 Hz, 1H), 5.17–5.04 (m, 3H), 4.15 (dq, *J* = 4.7, 1.6 Hz, 2H), 3.33 (dd, *J* = 9.6, 4.4 Hz, 1H), 3.28 (dd, *J* = 9.6, 5.2 Hz, 1H), 2.20 (dddt, *J* = 13.7, 6.9, 5.5, 1.4 Hz, 1H), 2.14–2.06 (m, 2H), 0.72 (dd, *J* = 6.8, 1.0 Hz, 2H), 0.16 (s, 3H), 0.16 (s, 3H); ^13^C­{^1^H} NMR (126 MHz, CDCl_3_) δ 137.0, 135.5, 117.4, 114.6, 63.8, 41.1, 34.9, 22.3, 19.2, −1.2, −1.3. HRMS (ESI/QTOF) *m*/*z*: [M + H]^+^ Calcd for C_11_H_22_IOSi^+^ 325.0479. Found 325.0482.

#### (Z)-4-(Iodomethyl)-2,2-dimethyl-3,4,5,8-tetrahydro-2H-1,2-oxasilocine (**7**)

Prepared according to general procedure C using **6** (0.35 g, 1.08 mmol) and **Ru–I** (44 mg, 0.054 mmol). Purification by flash chromatography on silica (2:1 hexanes:dichloromethane) gave **7** as a colorless oil (0.17 g, 54%) as an oil. FTIR (ATR): 2954, 2915, 2848, 1640, 1472, 1462, 1252, 1218, 1070, 962, 917 cm^–1^; ^1^H NMR (500 MHz, CDCl_3_) δ 5.87–5.71 (m, 1H), 5.62 (dddt, *J* = 11.0, 9.8, 8.7, 1.4 Hz, 1H), 4.30 (dd, *J* = 5.2, 1.4 Hz, 2H), 3.25 – 3.12 (m, 2H), 2.74 (ddd, *J* = 13.2, 9.2, 4.0 Hz, 1H), 2.32 (ddd, *J* = 13.7, 8.4, 5.9 Hz, 1H), 2.05 (ddddd, *J* = 11.9, 10.1, 8.0, 6.0, 4.1 Hz, 1H), 0.86 (ddd, *J* = 14.7, 4.2, 0.9 Hz, 1H), 0.71 (dd, *J* = 14.7, 11.2 Hz, 1H), 0.15 (s, 3H), 0.10 (s, 3H). ^13^C­{^1^H} NMR (126 MHz, CDCl_3_) δ 131.6, 127.9, 61.0, 37.9, 31.7, 22.7, 17.5, −1.0, −1.2. HRMS (ESI/QTOF) *m*/*z*: [M + Na]^+^ Calcd for C_9_H_17_INaOSi^+^ 318.9986. Found. 318.9984.

#### Fluoro­(2-methylpent-4-en-1-yl)­diphenylsilane (**8**)

To a solution of diallyldiphenylsilane (0.21 g, 0.78 mmol) in DCM (0.50 mL) was added a solution of BF_3_·2AcOH (0.15 g, 0.78 mmol) in DCM (0.76 mL) slowly over 10 min. The resulting mixture was then stirred at room temperature for 4.5 h before quenching with water (10 mL) and extracting with DCM (3 × 10 mL). The combined organic extracts were dried over MgSO_4_, filtered, and concentrated by rotary evaporation. The resulting product **8** (0.22 g, 99%) was obtained as a pale yellow oil and used directly without further purification. FTIR (ATR): 2955, 2930, 2875, 1460, 1352, 1254, 1143, 1004, 969, 835, 774, 741, 724, cm^–1^; ^1^H NMR (500 MHz, CDCl_3_) δ 7.74–7.70 (m, 1H), 7.63–7.58 (m, 3H), 7.48–7.43 (m, 3H), 7.43–7.37 (m, 3H), 5.73 (ddtd, *J* = 17.2, 10.4, 7.0, 2.6 Hz, 1H), 5.02 – 4.92 (m, 2H), 2.12–2.05 (m, 1H), 2.05–1.97 (m, 1H), 1.89 (tdd, *J* = 9.0, 5.0, 4.0, 2.1 Hz, 1H), 1.37 (dddd, *J* = 18.5, 11.0, 5.6, 3.1 Hz, 1H), 1.11 (dddd, *J* = 15.6, 9.1, 6.6, 2.9 Hz, 1H), 0.96 (dd, *J* = 6.8, 2.7 Hz, 3H); ^13^C­{^1^H} NMR (126 MHz, CDCl_3_) δ 137.1, 134.5, 134.0, 132.1, 130.5, 128.3, 128.0, 116.2, 44.5, 28.5, 22.5, 21.9, 21.8; HRMS (ESI/QTOF) *m*/*z*: [M + Na]^+^ Calcd for C_18_H_21_FNaSi^+^ 307.1289. Found 307.1289.

#### (Allyloxy)­(2-methylpent-4-en-1-yl)­diphenylsilane (**9**)

To a solution of allyl alcohol (0.02 mL, 0.25 mmol) in THF (0.88 mL) at 0 °C was added a solution of potassium bis­(trimethylsilyl)­amide (0.50 mL, 0.5 M solution in toluene) and the mixture was stirred for 30 min. Fluorosilane **8** (0.22 g, 0.17 mmol) was then added and the solution was allowed to warm to room temperature and stirred for 15 h. The reaction was quenched with water (15 mL) and extracted with methyl *tert*-butyl ether (3 × 15 mL). The combined organic extracts were dried over MgSO_4_ and concentrated by rotary evaporation. The crude product was purified by flash chromatography on silica (10:1 hexanes:EtOAc, *R*_f_ = 0.3) to yield **9** (0.056 g, 89%) as a colorless oil. FTIR (ATR): 3068, 2920, 1589, 1427, 1400, 1203, 1110, 1059, 943, 909, 729 cm^–1^; ^1^H NMR (500 MHz, CDCl_3_) δ 7.62–7.57 (m, 4H), 7.42–7.34 (m, 6H), 5.94 (ddt, *J* = 17.1, 10.4, 4.6 Hz, 1H), 5.73 (ddt, *J* = 17.2, 10.2, 7.1 Hz, 1H), 5.35 (dq, *J* = 17.0, 1.9 Hz, 1H), 5.13 (dq, *J* = 10.4, 1.7 Hz, 1H), 4.97–4.89 (m, 2H), 4.23 (dt, *J* = 4.6, 1.7 Hz, 2H), 2.09–2.03 (m, 1H), 1.97 (dtt, *J* = 13.6, 6.9, 1.1 Hz, 1H), 1.85–1.75 (m, 1H), 1.34 (dd, *J* = 15.2, 5.0 Hz, 1H), 1.08 (dd, *J* = 15.2, 8.7 Hz, 1H), 0.93 (d, *J* = 6.7 Hz, 3H); ^13^C­{^1^H} NMR (126 MHz, CDCl_3_) δ 137.5, 136.8, 135.4, 135.3, 134.7, 129.8, 127.8, 115.9, 114.4, 64.3, 44.7, 28.7, 22.6, 21.5; HRMS (ESI/QTOF) *m*/*z*: [M + H]^+^ Calcd for C_21_H_27_OSi^+^ 323.1826. Found 323.1815.

#### (*Z*)-4-Methyl-2,2-diphenyl-3,4,5,8-tetrahydro-1,2-oxasilocine (**10**)

Prepared according to general procedure C using **9** (0.052 g, 0.16 mmol) and **Ru–I** (7 mg, 0.008 mmol). Purification by flash chromatography on silica (2:1 hexanes:dichloromethane) gave **10** (0.040 g, 84%) as a colorless oil. FTIR (ATR) 3078, 2917, 1591, 1420, 1389, 1211, 1109, 972, 910, 729 cm^–1^; ^1^H NMR (500 MHz, CDCl_3_) δ 7.65 (dd, *J* = 7.4, 1.8 Hz, 2H), 7.57 (dd, *J* = 7.6, 1.5 Hz, 2H), 7.39 (dddd, *J* = 18.6, 9.4, 4.4, 2.8 Hz, 6H), 5.79 (dt, *J* = 10.7, 5.2 Hz, 1H), 5.71 (dt, *J* = 11.1, 8.6 Hz, 1H), 4.52 (qd, *J* = 14.8, 5.1 Hz, 2H), 2.68–2.61 (m, 1H), 2.14–2.07 (m, 2H), 1.41 (dd, *J* = 15.2, 4.0 Hz, 1H), 1.20 (dd, *J* = 15.2, 10.1 Hz, 1H), 1.07 (d, *J* = 6.3 Hz, 3H); ^13^C­{^1^H} NMR (126 MHz, CDCl_3_) δ 134.4, 134.2, 134.0, 130.6, 130.5, 130.0, 129.6, 129.6, 128.1, 128.1, 127.9, 127.8, 116.3, 61.6, 34.6, 29.5, 25.2, 21.6. HRMS (ESI/QTOF) *m*/*z*: [M + H]^+^ Calcd for C_19_H_23_OSi^+^ 295.1513. Found 295.1498.

#### (2-(Iodomethyl)­pent-4-en-1-yl)­diphenyl­((1-phenylallyl)­oxy)­silane (**11**)

Prepared according to general procedure B using diallyldiphenylsilane (0.25 g, 0.94 mmol) and 1-phenylprop-2-en-1-ol^37^ (0.20 g, 1.48 mmol). Purification by flash chromatography on silica (10:1 hexanes:methyl *tert*-butyl ether) gave **11** (0.35 g, 70%) as a colorless oil and a 1:1 mixture of diastereomers; *Spectral data for the mixture of diastereomers:* FTIR (ATR) 3054, 2917, 1636, 1589, 1447, 1428, 1328, 1155, 1111, 951, 781, 696 cm^–1^; ^1^H NMR (500 MHz, CDCl_3_) δ 7.61–7.56 (m, 8H), 7.51–7.47 (m, 8H), 7.44–7.22 (m, 14H), 5.97–5.87 (m, 2H), 5.62–5.40 (m, 2H), 5.22–5.12 (m, 4H), 5.05 (d, *J* = 10.7 Hz, 2H), 4.96 (d, *J* = 9.4 Hz, 2H), 3.24–3.12 (m, 2H), 3.06–3.01 (m, 2H), 2.15–1.87 (m, 6H), 1.30–1.10 (m, 4H), 0.85 – 0.75 (m, 2H); ^13^C­{^1^H} NMR (126 MHz, CDCl_3_) δ 135.6, 135.5, 134.8, 130.0, 128.3, 127.9, 127.9, 127.8, 127.4, 126.4, 126.4, 117.3, 117.2, 114.2, 114.2, 41.3, 41.0, 34.3, 34.2, 20.2, 20.1, 19.9 19.8. HRMS (ESI/QTOF) *m*/*z*: [M + Na]^+^ Calcd for C_27_H_29_INaOSi^+^ 547.0925. Found 547.0931.

#### (2-(Iodomethyl)­pent-4-en-1-yl)­(non-1-en-3-yloxy)­diphenylsilane (**12**)

Prepared according to general procedure B using diallyldiphenylsilane (0.25 g, 0.95 mmol) and 1-octene-3-ol^38^ (0.2426 g, 1.8921 mmol). Purification by flash chromatography on silica (10:1 hexanes:methyl *tert*-butyl ether) gave **12** (0.38 g, 76%) as a colorless oil and a 1:1 mixture of diastereomers. *Spectral data for the mixture of diastereomers:* FTIR (ATR): 3069, 2918, 1636, 1590, 1427, 1401, 1389, 1155, 1110, 977, 832, 729 cm^–1^; ^1^H NMR (500 MHz, CDCl_3_) δ 7.62–7.54 (m, 8H), 7.43–7.34 (m, 12H), 5.82 (dddd, *J* = 17.2, 10.4, 6.8, 3.6 Hz, 2H), 5.61 (ddt, *J* = 17.2, 10.2, 7.2 Hz, 2H), 5.05–4.97 (m, 8H), 4.14–4.09 (m, 2H), 3.28 (ddd, *J* = 9.5, 7.0, 4.1 Hz, 2H), 3.20 (dt, *J* = 9.5, 5.5 Hz, 2H), 2.15–2.07 (m, 2H), 2.06–1.99 (m, 2H), 1.57–1.47 (m, 6H), 1.46–1.38 (m, 2H), 1.28 – 1.10 (m, 14H), 0.85 (td, *J* = 7.3, 1.5 Hz, 6H); ^13^C­{^1^H} NMR (126 MHz, CDCl_3_) δ 140.9, 140.9, 135.7, 135.6, 135.2, 135.0, 134.9, 134.5, 129.9, 128.0, 127.8, 127.8, 127.6, 117.3, 114.7, 75.1, 41.4, 41.1, 37.7, 34.4, 31.7, 24.5, 22.6, 20.1, 19.9, 14.0. HRMS (ESI/QTOF) *m*/*z*: [M + Na]^+^ Calcd for C_26_H_35_INaOSi^+^ 541.1394. Found 541.1415.

#### (*Z*)-4-(Iodomethyl)-2,2,8-triphenyl-3,4,5,8-tetrahydro-2H-1,2-oxasilocine (**13**)

Prepared according to general procedure C using **11** (0.08 g, 0.15 mmol) and **Ru–II** (6 mg, 0.008 mmol). Purification by flash chromatography on silica (2:1 hexanes:dichloromethane) gave **13** (0.05 g, 67%) as a colorless oil and a 1:1 mixture of diastereomers; *Spectral data for the mixture of diastereomers:* FTIR (ATR): 2954, 1731, 1697, 1602, 1436, 1213, 1053, 974, 812, 701 cm^–1^; ^1^H NMR (500 MHz, CDCl_3_) δ 7.68–7.56 (m, 8H), 7.52–7.46 (m, 4H), 7.45–7.28 (m, 18H), 6.00 (dd, *J* = 10.8, 6.8 Hz, 1H), 5.81–5.70 (m, 4H), 5.56 (ddd, *J* = 11.2, 9.6, 8.3 Hz, 1H), 3.32 (dd, *J* = 9.6, 6.1 Hz, 1H), 3.29 (dd, *J* = 9.6, 7.2 Hz, 1H), 3.20–3.14 (m, 2H), 2.91 (ddd, *J* = 12.8, 9.0, 3.4 Hz, 1H), 2.67 (dt, *J* = 13.3, 6.4 Hz, 1H), 2.62–2.56 (m, 1H), 2.55–2.48 (m, 1H), 2.40 (ddd, *J* = 13.2, 9.0, 6.8 Hz, 1H), 2.07 (dtp, *J* = 16.5, 6.7, 3.8, 3.4 Hz, 1H), 1.74 (dd, *J* = 15.1, 5.3 Hz, 1H), 1.65 (dd, *J* = 15.1, 3.4 Hz, 1H), 0.90 (t, *J* = 6.8 Hz, 2H); ^13^C­{^1^H} NMR (126 MHz, CDCl_3_) δ 143.8, 143.3, 136.5, 135.7, 134.7, 134.2, 134.1, 134.0, 133.9, 130.2, 129.9, 129.8, 129.7, 128.8, 128.4, 128.0, 127.9, 127.9, 127.2, 127.1, 125.9, 125.8, 72.6, 70.6, 38.5, 37.1, 32.9, 32.2, 20.9, 19.9, 18.5, 15.4; HRMS (ESI/QTOF) *m*/*z*: [M + H]^+^ Calcd for C_25_H_26_IOSi^+^ 497.0792. Found 497.0794.

#### (*Z*)-8-Hexyl-4-(iodomethyl)-2,2-diphenyl-3,4,5,8-tetrahydro-1,2-oxasilocine (**14**)

Prepared according to general procedure C using **12** (0.14 g, 0.26 mmol) and **Ru–II** (11 mg, 0.013 mmol). Purification by flash chromatography on silica (2:1 hexanes:dichloromethane) gave **14** (0.124 g, 88%) as a colorless oil and a 1:1 mixture of diastereomers; *Spectral data for the mixture of diastereomers:* FTIR (ATR) 2955, 1732, 1699, 1606, 1436, 1265, 1170, 830, 734 cm^–1^; ^1^H NMR (500 MHz, CDCl_3_) δ 7.58 (tt, *J* = 7.9, 1.9 Hz, 6H), 7.44–7.30 (m, 14H), 5.78 (dd, *J* = 11.0, 6.2 Hz, 1H), 5.66–5.56 (m, 2H), 5.45 (dt, *J* = 11.2, 8.9 Hz, 1H), 4.57 (dq, *J* = 12.8, 6.2 Hz, 2H), 3.26 (dd, *J* = 9.6, 6.0 Hz, 1H), 3.22 (dd, *J* = 9.6, 7.4 Hz, 1H), 3.12 (dd, *J* = 8.2, 6.3 Hz, 1H), 3.09 (dd, *J* = 8.2, 5.4 Hz, 1H), 2.79 (ddd, *J* = 12.8, 8.9, 3.5 Hz, 1H), 2.54 (dt, *J* = 13.1, 6.6 Hz, 1H), 2.49–2.45 (m, 1H), 2.44–2.38 (m, 1H), 2.24 (ddd, *J* = 13.2, 8.8, 6.4 Hz, 1H), 2.02–1.95 (m, 1H), 1.78–1.71 (m, 2H), 1.66–1.59 (m, 3H), 1.56–1.50 (m, 1H), 1.44–1.39 (m, 1H), 1.36–1.16 (m, 13H), 0.89 (td, *J* = 7.0, 2.9 Hz, 6H); ^13^C­{^1^H} NMR (126 MHz, CDCl_3_) δ 136.7, 136.3, 134.2, 134.0, 133.9, 129.9, 129.7, 129.6, 129.5, 128.2, 127.8, 127.8, 127.6, 71.2, 69.5, 38.4, 38.0, 37.7, 37.1, 32.6, 32.3, 31.8, 25.3, 25.2, 22.7, 20.6, 19.5, 18.5, 15.6, 14.1. HRMS (ESI/QTOF) *m*/*z*: [M + H]^+^ Calcd for C_24_H_32_IOSi^+^ 491.1267. Found 491.1275.

#### 4-(Iodomethyl)-2,2-diphenyl-6-((*E*)-styryl)-1,2-oxasilinane (**15**)

Prepared according to the general procedure A using **13** (0.2 g, 0.4 mmol). Purification by flash chromatography on silica (10:1 hexanes:methyl *tert*-butyl ether) gave **15** (0.15 g, 75%) as a colorless oil and a 9:1 mixture of diastereomers. FTIR (ATR): 2930, 2855, 1598, 1428, 1116, 1092, 1061, 1027, 995,, 908, 801, 758, 740, 713, 695 cm^–1^. ^1^H NMR (500 MHz, CDCl_3_) δ 7.71 (dd, *J* = 7.6, 1.8 Hz, 2H), 7.61 (dd, *J* = 8.0, 1.5 Hz, 2H), 7.55–7.45 (m, 3H), 7.39 (dd, *J* = 7.0, 1.2 Hz, 2H), 7.35–7.29 (m, 4H), 7.25–7.22 (m, 2H), 6.73 (dd, *J* = 15.9, 1.4 Hz, 1H), 6.33 (dd, *J* = 15.8, 5.3 Hz, 1H), 4.65 (ddt, *J* = 11.2, 5.4, 1.8 Hz, 1H), 3.30 (dd, *J* = 9.6, 5.4 Hz, 1H), 3.20 (dd, *J* = 9.7, 7.0 Hz, 1H), 2.10 (dq, *J* = 13.4, 2.3 Hz, 1H), 2.08–1.97 (m, 1H), 1.57 (ddd, *J* = 14.3, 3.1, 2.3 Hz, 1H), 1.38 (dt, *J* = 13.5, 11.3 Hz, 1H), 0.87 (dd, *J* = 14.4, 12.9 Hz, 1H). ^13^C­{^1^H} NMR (126 MHz, CDCl_3_) δ 136.9, 134.3, 134.3, 131.9, 130.3, 130.2, 129.0, 128.5, 128.3, 127.9, 127.4, 126.5, 74.5, 42.6, 36.9, 18.8, 18.4. HRMS (ESI/QTOF) *m*/*z*: [M + H]^+^ Calcd for C_25_H_26_IOSi^+^ 497.0792. Found 497.0792.

#### 6-((*E*)-Hept-1-en-1-yl)-4-(iodomethyl)-2,2-diphenyl-1,2-oxasilinane (**16**)

Prepared according to the general procedure A using **14** (0.08 g, 0.4 mmol). Purification by flash chromatography on silica (10:1 hexanes:methyl *tert*-butyl ether) gave **16** (0.057 g, 72%) as a colorless oil and a 8:1 mixture of diastereomers. FTIR (ATR): 2961, 2929, 2851, 1598, 1428, 1365, 1157, 1121, 963, 950, 840,, 758, 738, 714, 696 cm^–1^. ^1^H NMR (500 MHz, CDCl_3_) δ 7.72–7.67 (m, 1H), 7.59–7.56 (m, 2H), 7.47 (dd, *J* = 8.2, 6.5 Hz, 2H), 7.44–7.38 (m, 3H), 7.36 (dd, *J* = 7.8, 6.4 Hz, 2H), 5.86–5.72 (m, 1H), 5.67–5.57 (m, 1H), 4.43 (dd, *J* = 11.0, 6.0 Hz, 1H), 3.28 (dd, *J* = 9.6, 5.3 Hz, 1H), 3.19 (dd, *J* = 9.6, 6.9 Hz, 1H), 2.07 (q, *J* = 7.2 Hz, 2H), 2.02–1.91 (m, 1H), 1.53 (dt, *J* = 14.3, 2.8 Hz, 1H), 1.43 (p, *J* = 7.3 Hz, 2H), 1.32 (tddd, *J* = 11.3, 8.4, 5.9, 3.6 Hz, 6H), 0.92 (t, *J* = 6.9 Hz, 3H), 0.82 (dd, *J* = 14.3, 12.8 Hz, 1H). ^13^C­{^1^H} NMR (126 MHz, CDCl_3_) δ 134.30, 134.28, 132.2, 131.0, 130.2, 130.1, 128.2, 127.8, 74.8, 42.7, 36.8, 32.1, 31.5, 28.8, 22.5, 19.1, 18.4, 14.0. HRMS (ESI/QTOF) *m*/*z*: [M + H]^+^ Calcd for C_24_H_32_IOSi^+^ 491.1262. Found 491.1260.

#### 2,2-Diphenyl-4-((phenylsulfonyl)­methyl)-6-vinyl-1,2-oxasilinane (**17**)

To a solution of *cis-* **2** (100 mg, 0.24 mmol) in DMF (2.4 mL) was added NaHCO_3_ (22 mg, 0.26 mmol) and sodium benzenesulfinate (60 mg, 0.36 mmol) and the mixture was heated by MW to 100 °C with stirring for 0.5 h. After cooling to room temperature, the reaction mixture was diluted with water (10 mL) and ethyl acetate (10 mL). The layers were separated and the aqueous phase was extracted with ethyl acetate (2 × 10 mL). The combined organic layers were washed with saturated aqueous sodium chloride solution (10 mL), dried over MgSO_4_, filtered, and concentrated on a rotary evaporator. The crude product was purified by flash chromatography on silica (10:1 hexanes:EtOAc) to yield **17** (87 mg, 83%) as a colorless oil. FTIR (ATR): cm^–1^. ^1^H NMR (500 MHz, CDCl_3_) δ 7.95–7.86 (m, 2H), 7.67–7.60 (m, 3H), 7.56–7.51 (m, 4H), 7.49–7.44 (m, 1H), 7.43–7.40 (m, 2H), 7.39–7.35 (m, 1H), 7.34–7.31 (m, 2H), 5.90 (ddd, *J* = 17.1, 10.5, 4.8 Hz, 1H), 5.36 (dt, *J* = 17.1, 1.7 Hz, 1H), 5.10 (dt, *J* = 10.5, 1.6 Hz, 1H), 4.47–4.39 (m, 1H), 3.12 (d, *J* = 6.2 Hz, 2H), 2.60–2.48 (m, 1H), 2.04–1.95 (m, 1H), 1.59 (ddd, *J* = 14.5, 3.4, 2.2 Hz, 1H), 1.39 (dt, *J* = 13.6, 11.5 Hz, 1H), 0.94–0.83 (m, 1H). ^13^C NMR (126 MHz, CDCl_3_) δ 140.1, 140.0, 134.3, 134.2, 133.7, 130.3, 130.2, 129.3, 128.2, 127.9, 127.8, 113.9, 74.3, 65.3, 42.1, 30.1, 18.4. HRMS (ESI/QTOF) *m*/*z*: [M + H]^+^ Calcd for C_25_H_26_O_3_SSi^+^ 435.1445. Found 435.1442.

#### 2-((Phenylsulfonyl)­methyl)­hex-5-ene-1,4-diol (**18**)

To a solution of **17** (80 mg, 0.18 mmol) in THF (1 mL) and MeOH (1 mL) at room temperature was added KHCO_3_ (36 mg, 0.36 mmol) and KF•2H_2_O (25 mg, 0.27 mmol) followed by the addition of 30% H_2_O_2_ (60 μL, 0.54 mmol). The resulting mixture was stirred for 2 h and the remaining hydrogen peroxide was decomposed by the careful dropwise addition of aqueuous saturated sodium thiosulfate (10 mL). The mixture was extracted with ethyl acetate (3 × 10 mL) and the combined extracts were dried over MgSO, filtered, and concentrated by a rotary evaporator. The crude product was purified by flash chromatography on silica (1:1 to 1:2 hexanes:EtOAc) to yield **18** (38 mg, 78%) as a colorless oil. ^1^H NMR (500 MHz, CDCl_3_) δ 7.99–7.91 (m, 2H), 7.71–7.65 (m, 1H), 7.63–7.55 (m, 2H), 5.85 (ddd, *J* = 17.2, 10.4, 5.9 Hz, 1H), 5.24 (d, *J* = 17.2 Hz, 1H), 5.12 (d, *J* = 10.4 Hz, 1H), 4.24 (dddt, *J* = 9.0, 6.1, 3.4, 1.3 Hz, 1H), 3.77 (dd, *J* = 11.3, 4.9 Hz, 1H), 3.70 (dd, *J* = 11.3, 5.1 Hz, 1H), 3.34 (dd, *J* = 14.3, 6.4 Hz, 1H), 3.15 (dd, *J* = 14.3, 5.4 Hz, 1H), 2.45 (dddd, *J* = 11.9, 6.8, 5.1, 1.7 Hz, 1H), 1.79 (ddd, *J* = 14.5, 7.0, 3.5 Hz, 1H), 1.66 (ddd, *J* = 14.5, 9.0, 6.8 Hz, 1H). ^13^C­{^1^H} NMR (126 MHz, CDCl_3_) δ 140.4, 139.7, 133.8, 129.4, 127.9, 115.0, 71.1, 64.8, 57.9, 38.7, 33.8. HRMS (ESI/QTOF) *m*/*z*: [M + H]^+^ Calcd for C_13_H_19_O_4_S^+^ 271.0999. Found 271.0999.

#### (*E*)-4,7-Dihydroxy-6-((phenylsulfonyl)­methyl)­hept-2-en-1-yl Acetate (**19**)

To a solution of **18** (30 mg, 0.1 mmol) in toluene (1.0 mL) was added *cis*-1,4-diacetoxy-2-butene (50 μL, 0.3 mmol) followed by **Ru–II** (4 mg, 0.005 mmol) and the mixture was stirred for 8 h before concentrating on a rotary evaporator. Purification of the crude product by flash chromatography on silica (1:1 to 2:1 hexanes:ethyl acetate) gave **19** (31 mg, 92%) as a colorless oil. ^1^H NMR (500 MHz, CDCl_3_) δ 7.96–7.90 (m, 2H), 7.71–7.63 (m, 1H), 7.62–7.55 (m, 2H), 5.84–5.72 (m, 2H), 4.59–4.52 (m, 2H), 4.29 (t, *J* = 4.9 Hz, 1H), 3.74 (dd, *J* = 11.1, 5.0 Hz, 1H), 3.68 (dd, *J* = 11.0, 5.2 Hz, 1H), 3.33 (dd, *J* = 14.3, 6.1 Hz, 1H), 3.13 (dd, *J* = 14.3, 5.6 Hz, 1H), 2.44 (p, *J* = 5.9 Hz, 1H), 2.38 (s, 2H), 2.07 (s, 3H), 1.80 (ddd, *J* = 14.5, 7.0, 3.3 Hz, 1H), 1.67 (ddd, *J* = 14.9, 9.0, 6.6 Hz, 1H). ^13^C­{^1^H} NMR (126 MHz, CDCl_3_) δ 170.8, 139.7, 136.4, 133.9, 129.4, 127.9, 124.7, 69.9, 64.8, 64.1, 57.8, 38.7, 33.7, 20.9. HRMS (ESI/QTOF) *m*/*z*: [M + H]^+^ Calcd for C_16_H_23_O_6_S^+^ 343.1210. Found 343.1219.

### Conformational Analysis by DFT

Geometries of gas-phase molecules were optimized within density functional theory (DFT) using the ORCA 4.1 software package, PBE functional (a generalized gradient approximation), and def2-TZVP basis set. For calculations of the dimethyl-substituted molecules, PBE results were compared to calculations using the B3LYP hybrid functional and def2-TZVP basis set, and we observed no significant differences in optimized geometry or relative structural energy. To ensure that minimum-energy structures were identified, the diphenyl-substituted molecules were optimized using multiple initial geometries that differed in the way the phenyl groups were oriented.

## Supplementary Material



## Data Availability

The data underlying this study are available in the published article and its Supporting Information.

## Notes

Date from time-course and equilibration studies, stereochemical assignment of oxasilinanes by NMR, summary of computational results, and copies of ^1^H NMR and ^13^C­{^1^H} spectra (PDF)
