# Supplementary material for: Stereoselective Substituted Oxasilinane Synthesis from Diallylsilanes via Electrophile-Promoted Rearrangement, RCM, and Re-Catalyzed Allylic Transposition
Source: J Org Chem. 2026 Jul 24;91(31):10761–8. doi: 10.1021/acs.joc.6c00566 (PMC13459414; doi:10.1021/acs.joc.6c00566)
Supplement: Supplementary file 1 [file jo6c00566_si_001.pdf]

Supporting Information for:

Stereoselective Substituted Oxasilinane Synthesis from Diallylsilanes via Electrophile-Promoted Rearrangement, RCM, and Re-Catalyzed Allylic Transposition

Gregory W. O'Neil*, Inna A. Fomina, Christopher R. Myers, Zachary T. Rademacher, Rawley M. Spencer, and Robert F. Berger

Department of Chemistry, Western Washington University, Bellingham, WA 98229 (USA)
*Corresponding author. Email: oneilg@wwu.edu

Contents:

	Page
General Experimental Procedures	S2
**Figure S1.** ^{1}H NMR spectra for reaction of **1** with Re_2O_7 at different time increments.	S2
**Figure S2.** Monitoring reaction progress of **1** with Re_2O_7 by ^{1}H NMR	S3
**Figure S3.** Testing for equilibration of **2** with Re_2O_7 by ^{1}H NMR.	S3
**Figure S4.** COSY NMR and signal assignments for compound **2**.	S4
**Figure S5.** Stereochemical assignment of **2.**	S5
**Figure S6.** Summary of computational results.	S24
**NMR Spectra for Compound:**	**Page**
(*Z*)-4-(iodomethyl)-2,2-diphenyl-3,4,5,8-tetrahydro-2H-1,2-oxasilocine (**1**)	S6
4-(iodomethyl)-2,2-diphenyl-6-vinyl-1,2-oxasilinane (**2**)	S7
4-(iodomethyl)-2,2-dimethyl-6-vinyl-1,2-oxasilinane (**3**)	S8
4-methyl-2,2-diphenyl-6-vinyl-1,2-oxasilinane (**4**)	S9
(Allyloxy)-(2-(iodomethyl)pent-4-en-1-yl)dimethylsilane (**6**)	S10
(*Z*)-4-(iodomethyl)-2,2-dimethyl-3,4,5,8-tetrahydro-2H-1,2-oxasilocine (**7**)	S11
Fluoro(2-methylpent-4-en-1-yl)diphenylsilane (**8**)	S12
(Allyloxy)(2-methylpent-4-en-1-yl)diphenylsilane (**9**)	S13
(*Z*)-4-Methyl-2,2-diphenyl-3,4,5,8-tetrahydro-1,2-oxasilocine (**10**)	S14
(2-(Iodomethyl)pent-4-en-1-yl)diphenyl((1-phenylallyl)oxy)silane (**11**)	S15
(2-(Iodomethyl)pent-4-en-1-yl)(non-1-en-3-yloxy)diphenylsilane (**12**)	S16
(*Z*)-4-(Iodomethyl)-2,2,8-triphenyl-3,4,5,8-tetrahydro-2H-1,2-oxasilocine (**13**)	S17
(*Z*)-8-Hexyl-4-(iodomethyl)-2,2-diphenyl-3,4,5,8-tetrahydro-1,2-oxasilocine (**14**)	S18
4-(iodomethyl)-2,2-diphenyl-6-((*E*)-styryl)-1,2-oxasilinane (**15**)	S19
6-((*E*)-hept-1-en-1-yl)-4-(iodomethyl)-2,2-diphenyl-1,2-oxasilinane (**16**)	S20
2,2-diphenyl-4-((phenylsulfonyl)methyl)-6-vinyl-1,2-oxasilinane (**17**)	S21
2-((phenylsulfonyl)methyl)hex-5-ene-1,4-diol (**18**)	S22
(E)-4,7-dihydroxy-6-((phenylsulfonyl)methyl)hept-2-en-1-yl acetate (**19**)	S23

General Experimental Information. All reactions were carried out in vessels under an argon atmosphere unless otherwise specified. Dry solvents were prepared by passing the solvent through a column of activated alumina under nitrogen immediately prior to use. All reagents were purchased and used as received unless mentioned otherwise. TLC analysis used 0.25 mm silica layer fluorescence UV_{254} plates. Column chromatography: silica gel (230-400 mesh). IR: FT-IR with single-bounce diamond ATR. NMR: Spectra were recorded on a 500 MHz spectrometer in $CDCl_3$ or C_6D_6; chemical shifts (d) are given in ppm, coupling constants (*J*) in Hz. Solvent signals were used as references ($CDCl_3$: δ 77.0 ppm; residual $CHCl_3$ in $CDCl_3$: δ 7.26 ppm, C_6D_6: δ 128.06 ppm; residual C_6H_6 in C_6D_6 δ 7.16 ppm).

Figure S1. ^{1}H NMR spectra ($CDCl_3$, 500 MHz) obtained for reaction of **1** with Re_2O_7 at different durations.

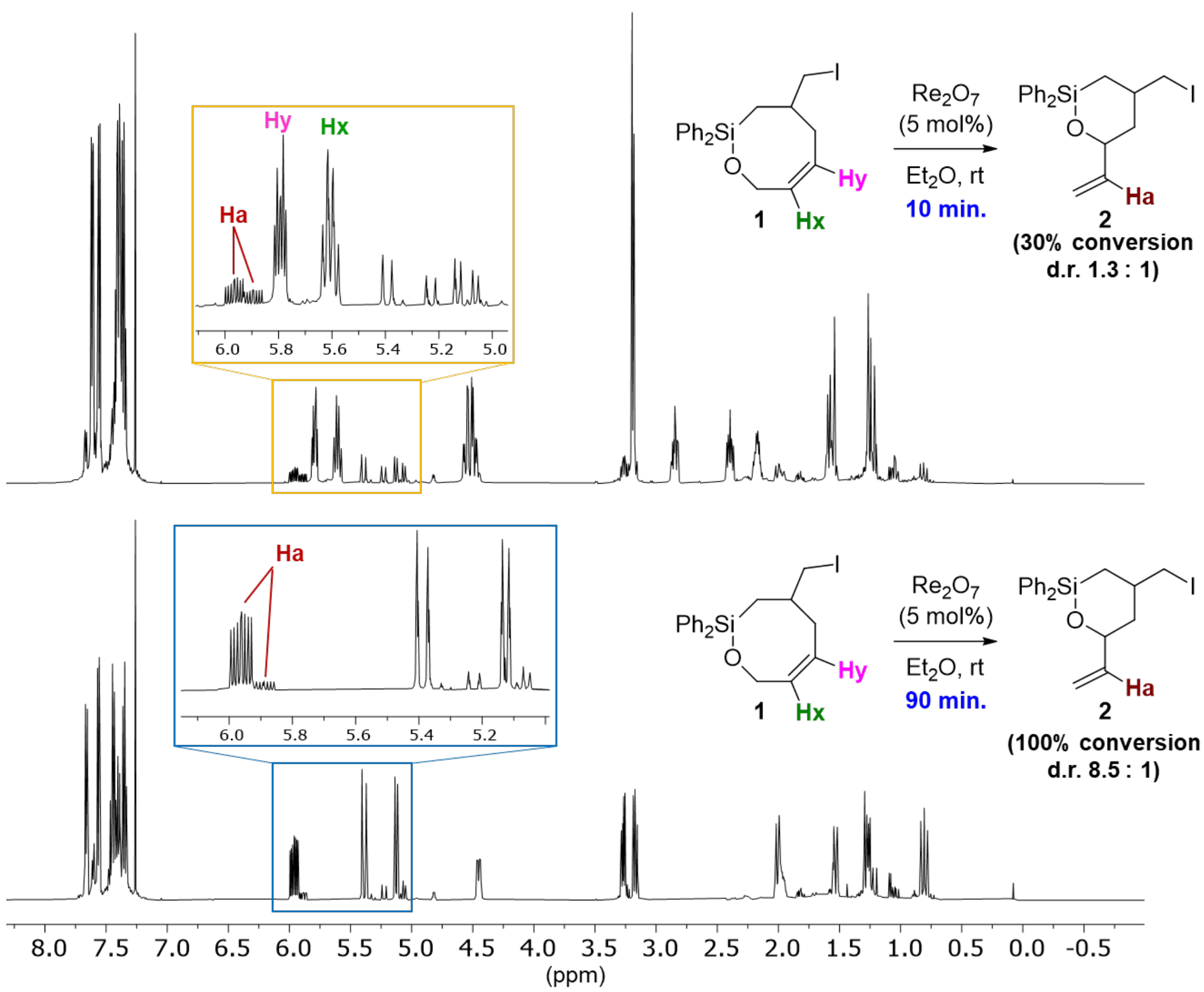

Figure S2. Monitoring reaction progress of **1** with Re_2O_7 by ^{1}H NMR ($CDCl_3$, 500 MHz).

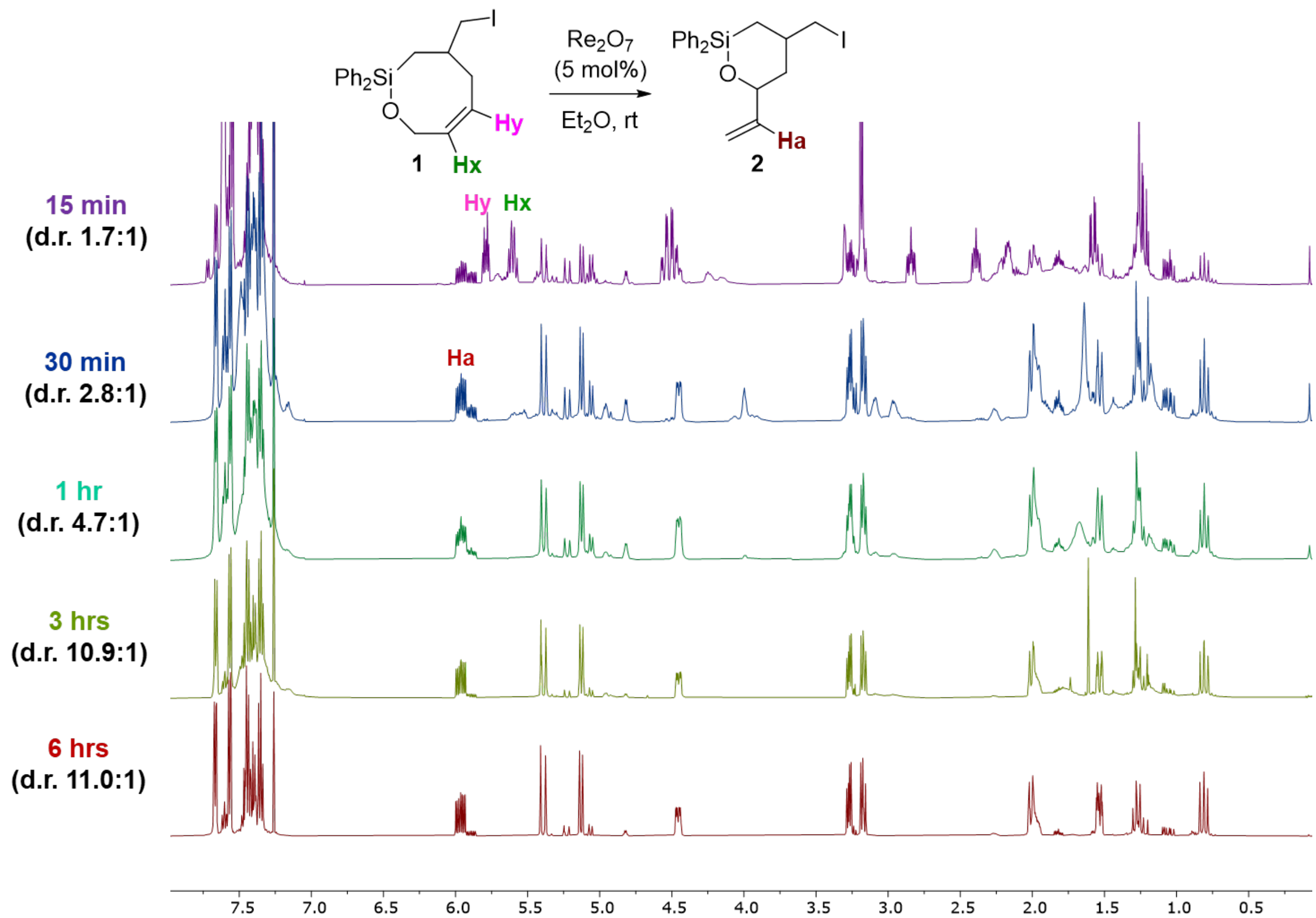

Figure S3. Testing for equilibration of **2** with Re_2O_7 by ^{1}H NMR ($CDCl_3$, 500 MHz).

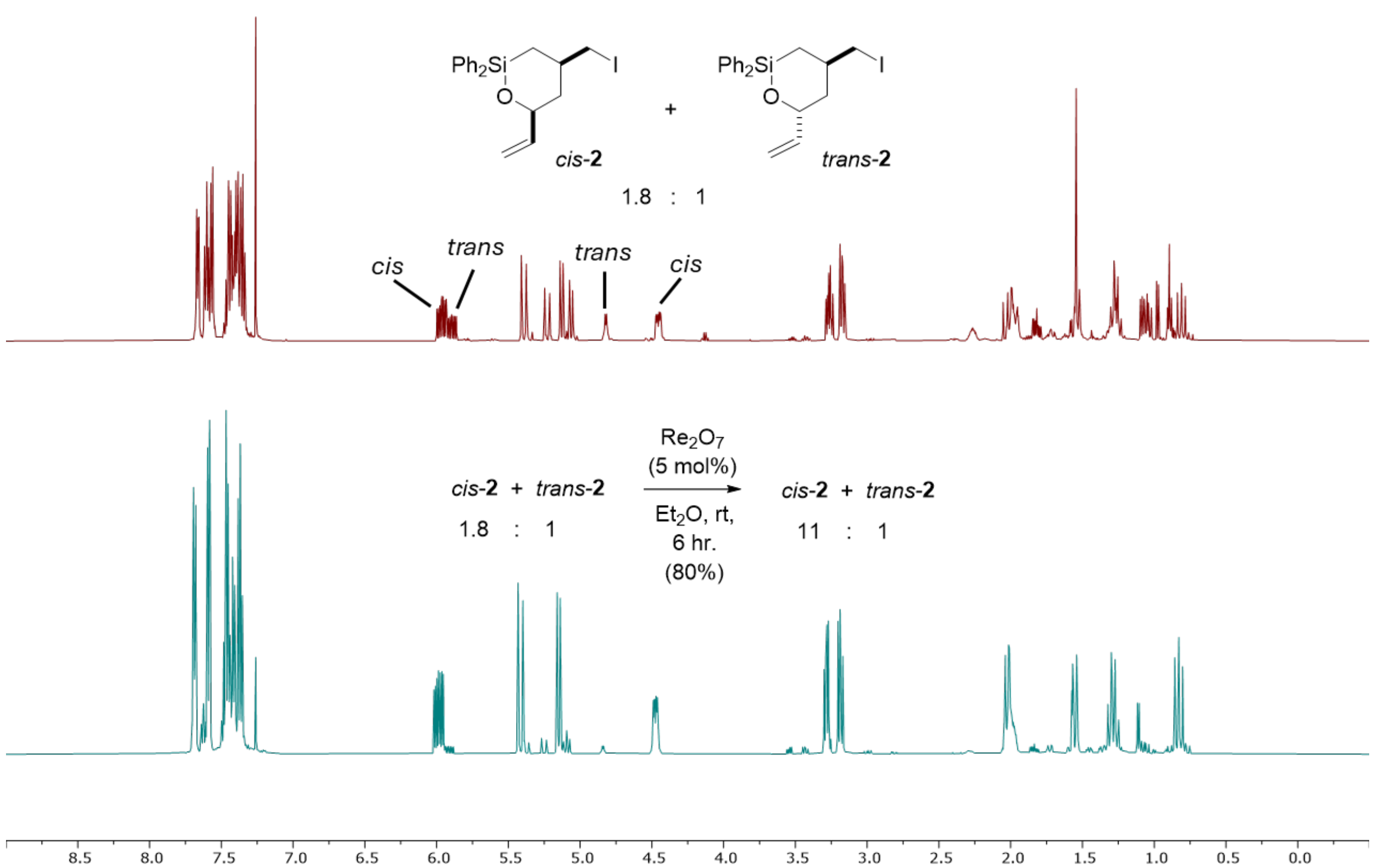

Figure S4. COSY NMR ($CDCl_3$, 500 MHz) and signal assignments for compound **2**.

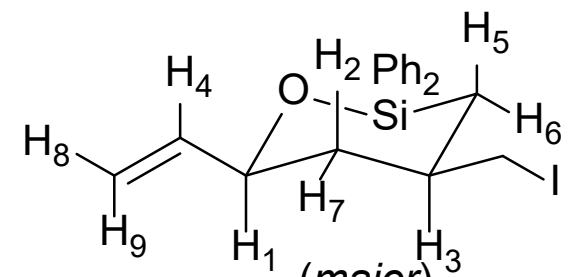

2 (*major*)

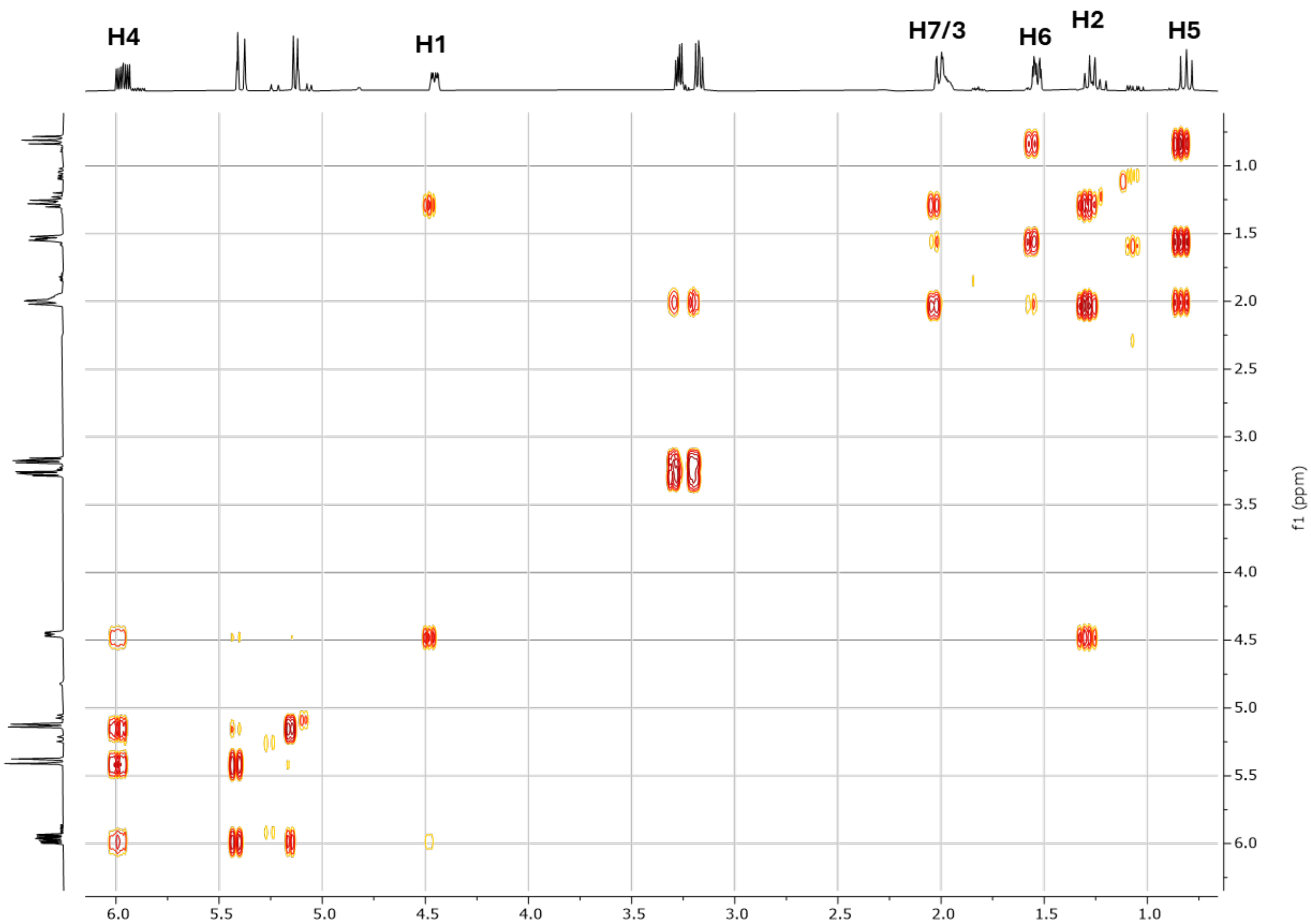

Signal	Chemical Shift (ppm)	Coupling Constants (Hz)
H1	4.46	11.3 ($J_{1,2}$), 5.1 ($J_{1,4}$), 1.7 ($J_{1,7}$), 1.7 ($J_{1,8}$), 1.7 ($J_{1,9}$)
H2	1.27	13.3 ($J_{2,7}$), 11.3 ($J_{2,1}$), 11.3 ($J_{2,3}$)
H3/7	2.12-1.93	m
H4	5.97	17.1 ($J_{4,9}$), 10.4 ($J_{4,8}$), 5.0 ($J_{4,1}$)
H5	0.81	14.4 ($J_{5,6}$), 13.0 ($J_{5,3}$)
H6	1.54	14.3 ($J_{6,5}$), 2.9 ($J_{6,3}$)
H8	5.13	1.6 ($J_{9,1}$), 1.6 ($J_{8,1}$)
H9	5.39	1.6 ($J_{9,1}$)

Figure S5. Stereochemical assignments of major and minor oxasilinane diastereomers.

^{1}H NMR (500 MHz, $CDCl_3$)

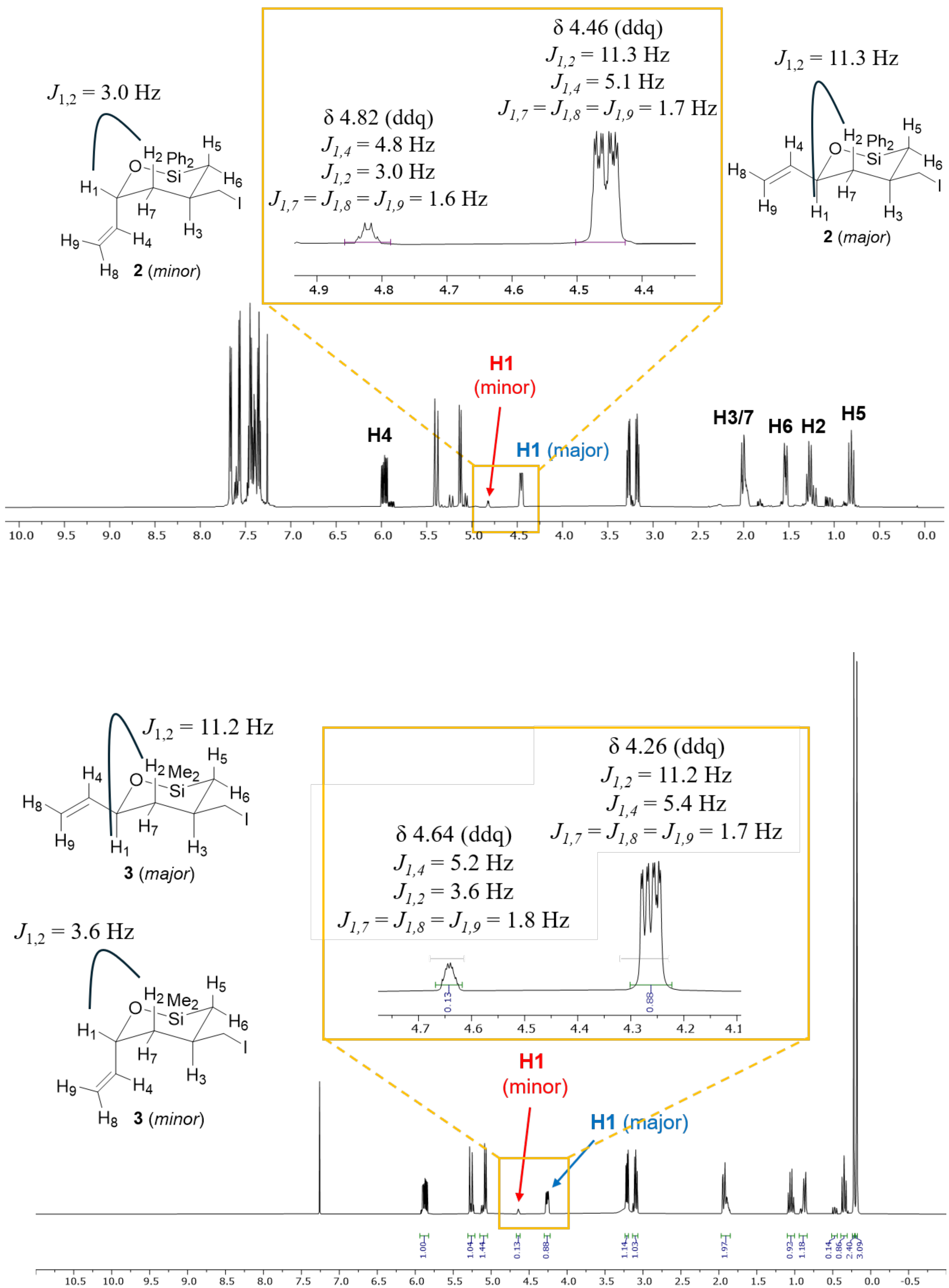

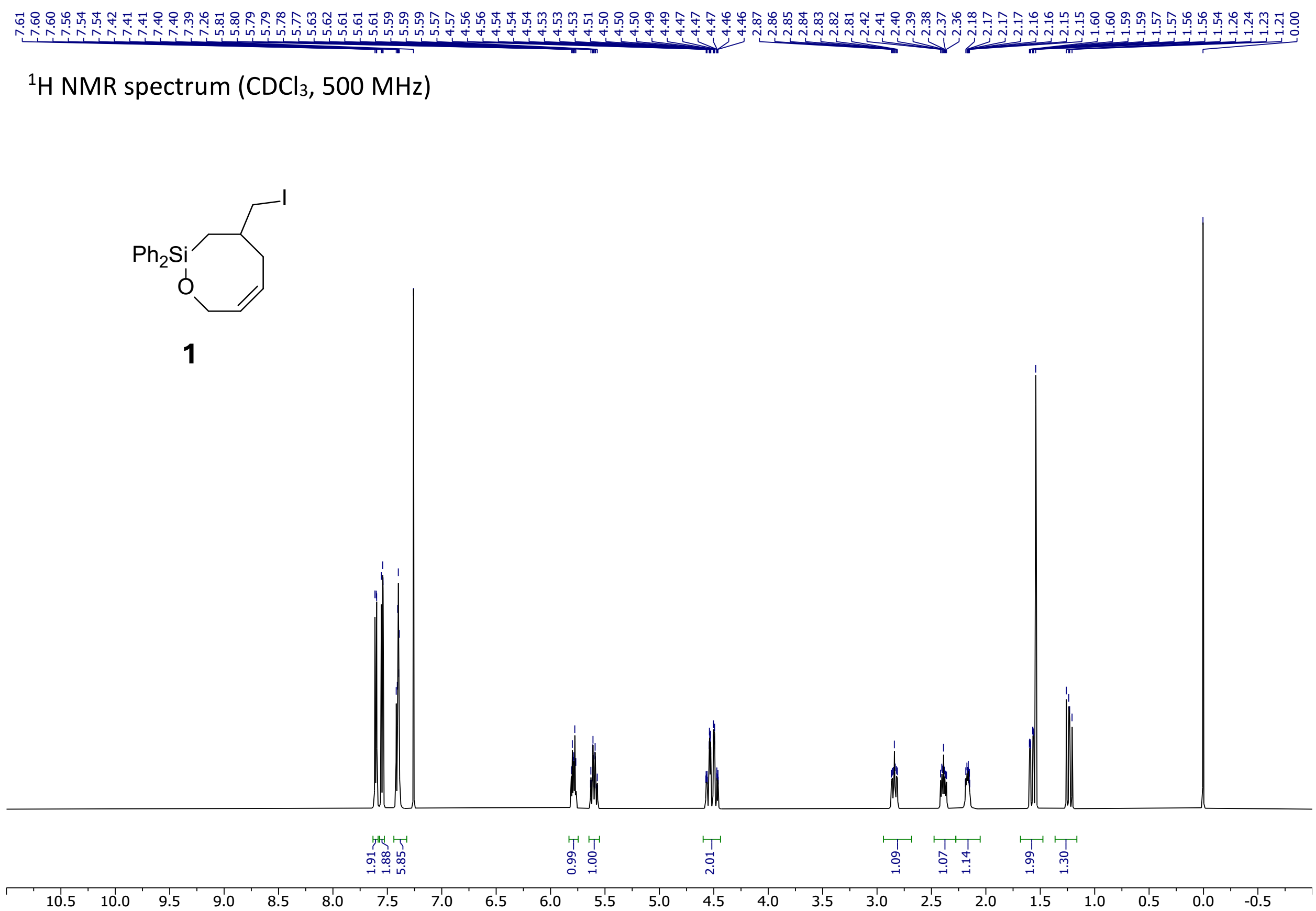
7.61 7.60 7.56 7.54 7.54 7.42 7.41 7.41 7.40 7.40 7.39 7.26 5.81 5.80 5.79 5.79 5.78 5.77 5.63 5.62 5.61 5.61 5.61 5.59 5.59 5.59 5.57 4.57 4.56 4.56 4.54 4.54 4.54 4.53 4.53 4.53 4.51 4.50 4.50 4.50 4.49 4.49 4.47 4.47 4.46 4.46 2.87 2.86 2.85 2.84 2.83 2.82 2.81 2.42 2.41 2.40 2.39 2.38 2.37 2.36 2.18 2.17 2.17 2.17 2.16 2.16 2.15 2.15 1.60 1.60 1.59 1.59 1.57 1.57 1.56 1.56 1.54 1.26 1.24 1.23 1.21 0.00
^{1}H NMR spectrum (CDCl₃, 500 MHz)
I
Ph$_2$Si
O
1
1.91 1.88 5.85 0.99 1.00 2.01 1.09 1.07 1.14 1.99 1.30
10.5 10.0 9.5 9.0 8.5 8.0 7.5 7.0 6.5 6.0 5.5 5.0 4.5 4.0 3.5 3.0 2.5 2.0 1.5 1.0 0.5 0.0 -0.5

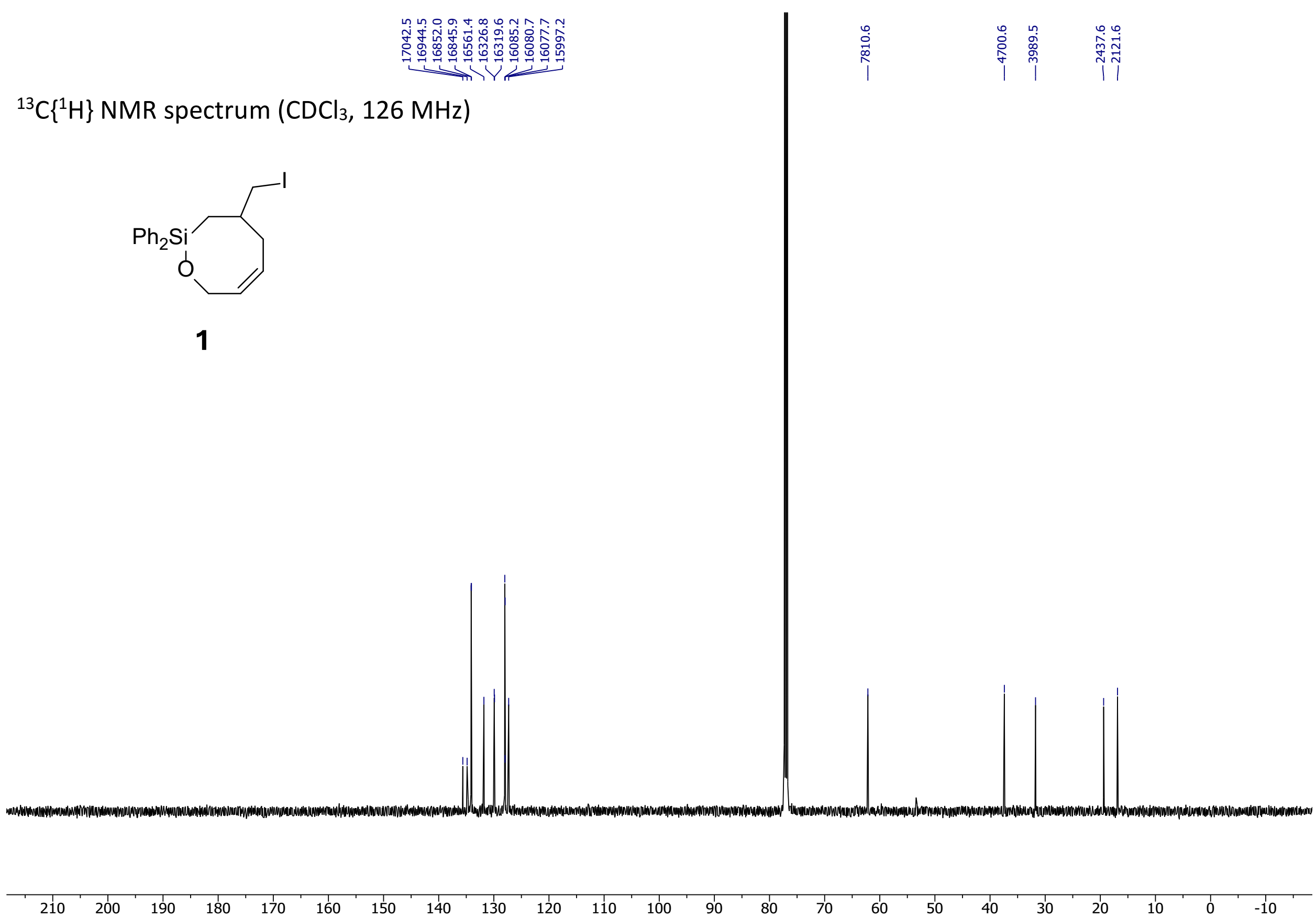
17042.5 16944.5 16852.0 16845.9 16561.4 16326.8 16319.6 16085.2 16080.7 16077.7 15997.2 7810.6 4700.6 3989.5 2437.6 2121.6
^{13}C{^{1}H} NMR spectrum (CDCl₃, 126 MHz)
I
Ph$_2$Si
O
1
210 200 190 180 170 160 150 140 130 120 110 100 90 80 70 60 50 40 30 20 10 0 -10

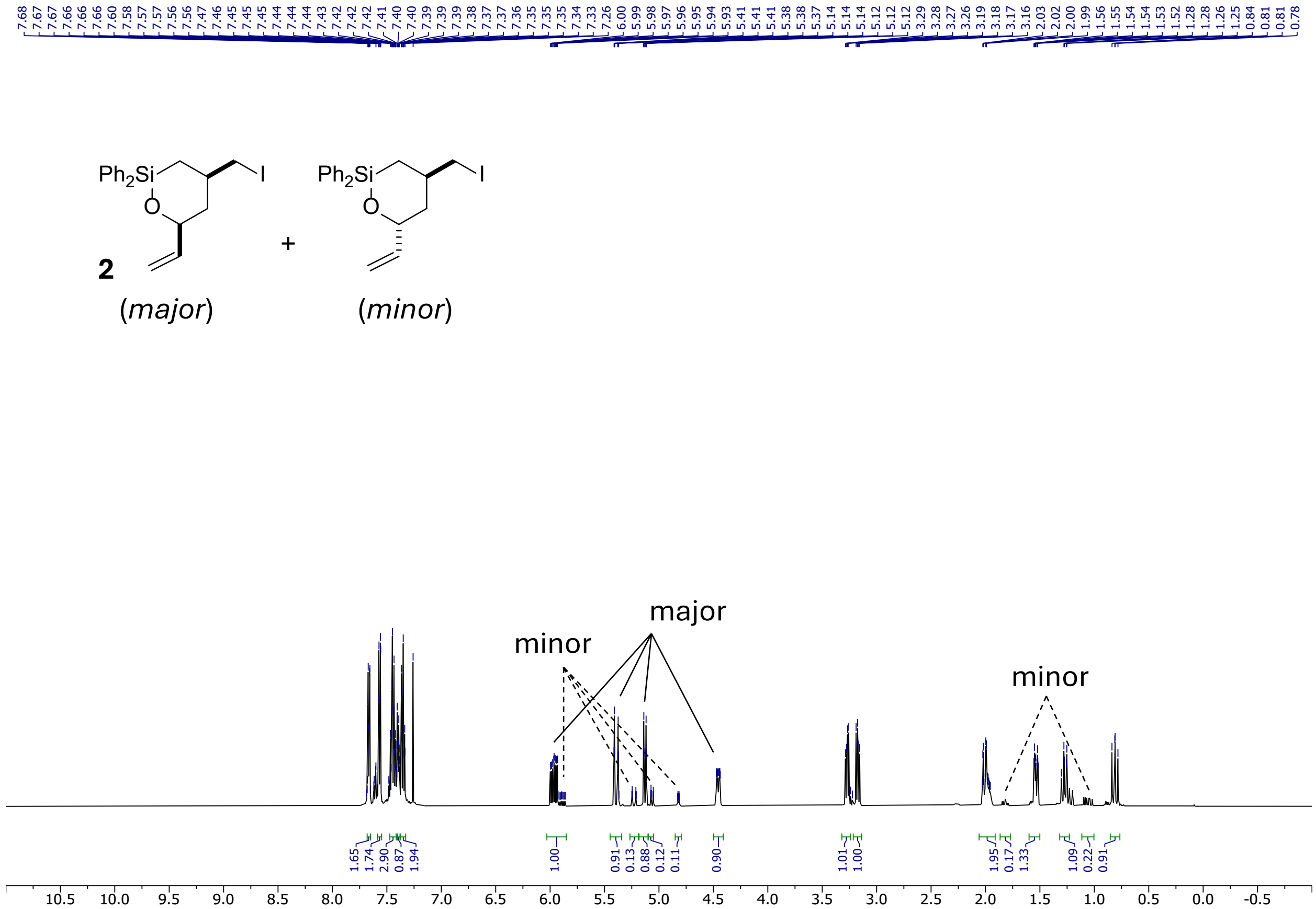
Ph2Si
O
I
2
(major)
+
(minor)
minor
major
minor

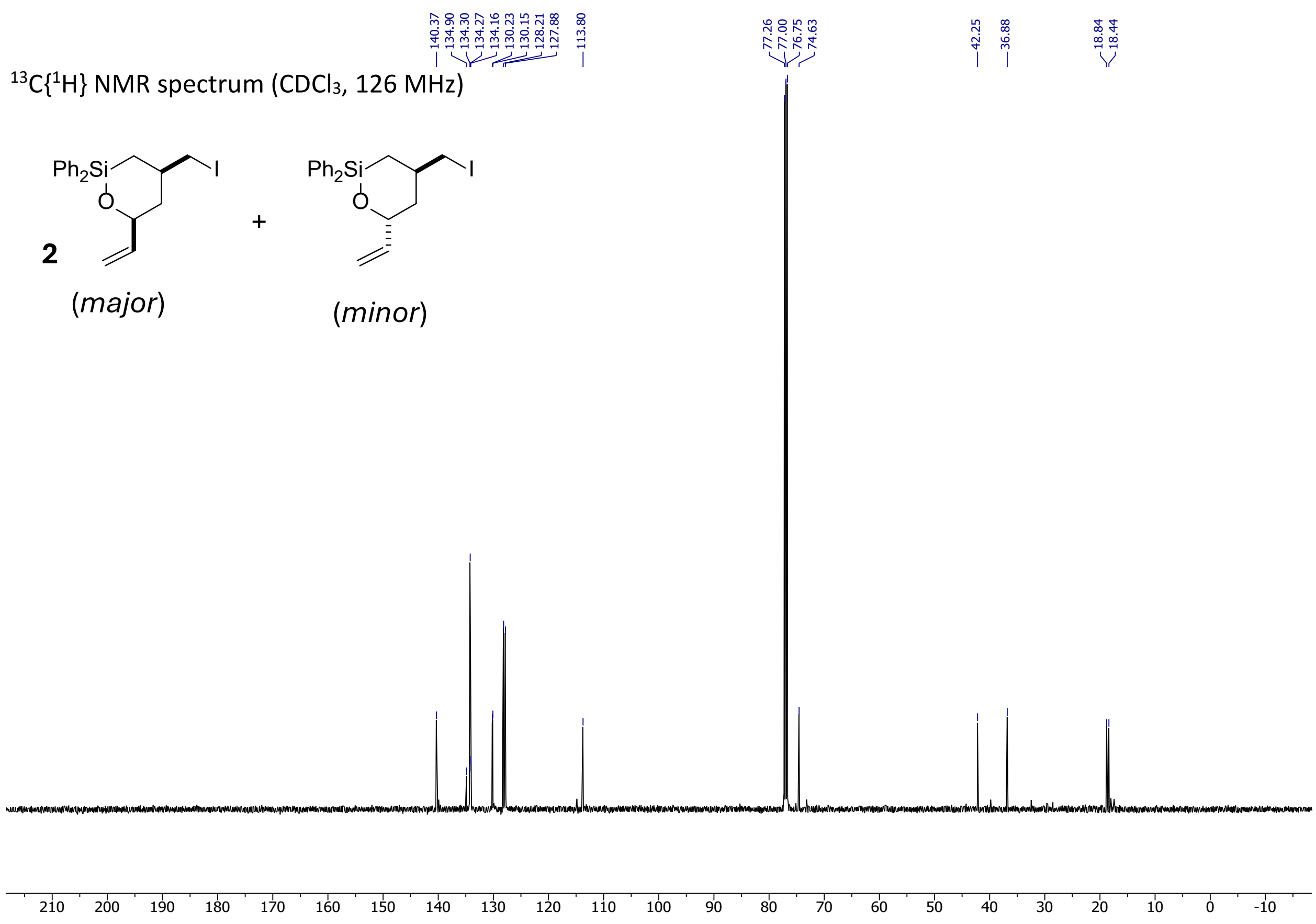
140.37
134.90
134.30
134.27
134.16
130.23
130.15
128.21
127.88
113.80
77.26
77.00
76.75
74.63
42.25
36.88
18.84
18.44
13C{1H} NMR spectrum (CDCl3, 126 MHz)
Ph2Si
O
I
2
(major)
+
(minor)

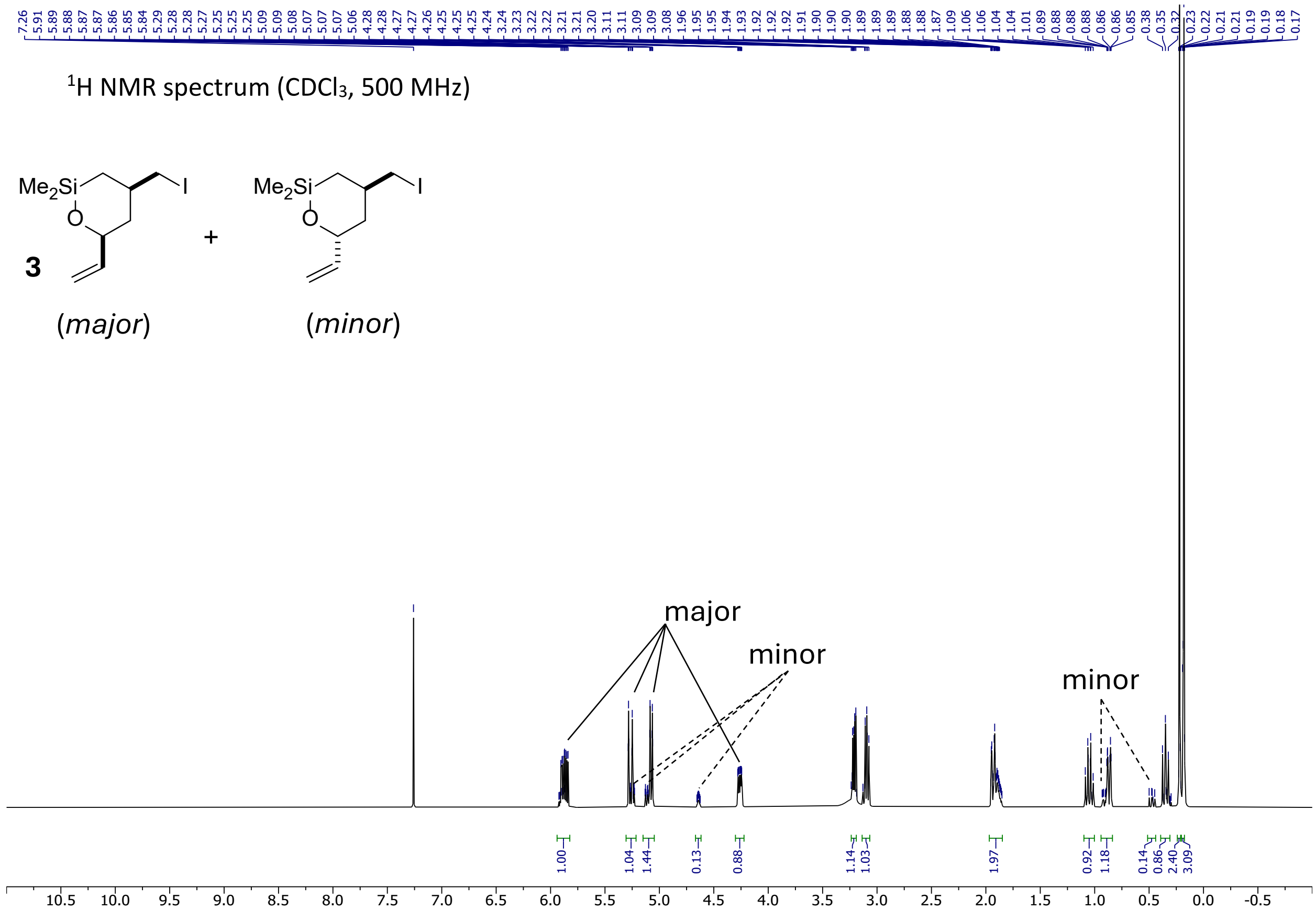
7.26 5.91 5.89 5.88 5.87 5.86 5.85 5.84 5.29 5.28 5.28 5.27 5.25 5.25 5.09 5.08 5.07 5.07 5.06 4.28 4.27 4.26 4.25 4.25 4.25 4.24 3.24 3.23 3.22 3.22 3.21 3.21 3.20 3.11 3.11 3.09 3.09 3.08 1.96 1.95 1.95 1.94 1.93 1.92 1.92 1.92 1.92 1.91 1.90 1.90 1.90 1.89 1.89 1.89 1.88 1.88 1.87 1.09 1.06 1.06 1.04 1.04 1.01 0.89 0.88 0.88 0.88 0.88 0.86 0.86 0.85 0.38 0.35 0.32 0.23 0.22 0.21 0.21 0.19 0.18 0.17
^{1}H NMR spectrum (CDCl$_3$, 500 MHz)
Me$_2$Si
O
I
3
+
(*major*)
(*minor*)
major
minor
minor
1.00 1.04 1.44 0.13 0.88 1.14 1.03 1.97 0.92 1.18 0.14 0.86 2.40 3.09
10.5 10.0 9.5 9.0 8.5 8.0 7.5 7.0 6.5 6.0 5.5 5.0 4.5 4.0 3.5 3.0 2.5 2.0 1.5 1.0 0.5 0.0 -0.5

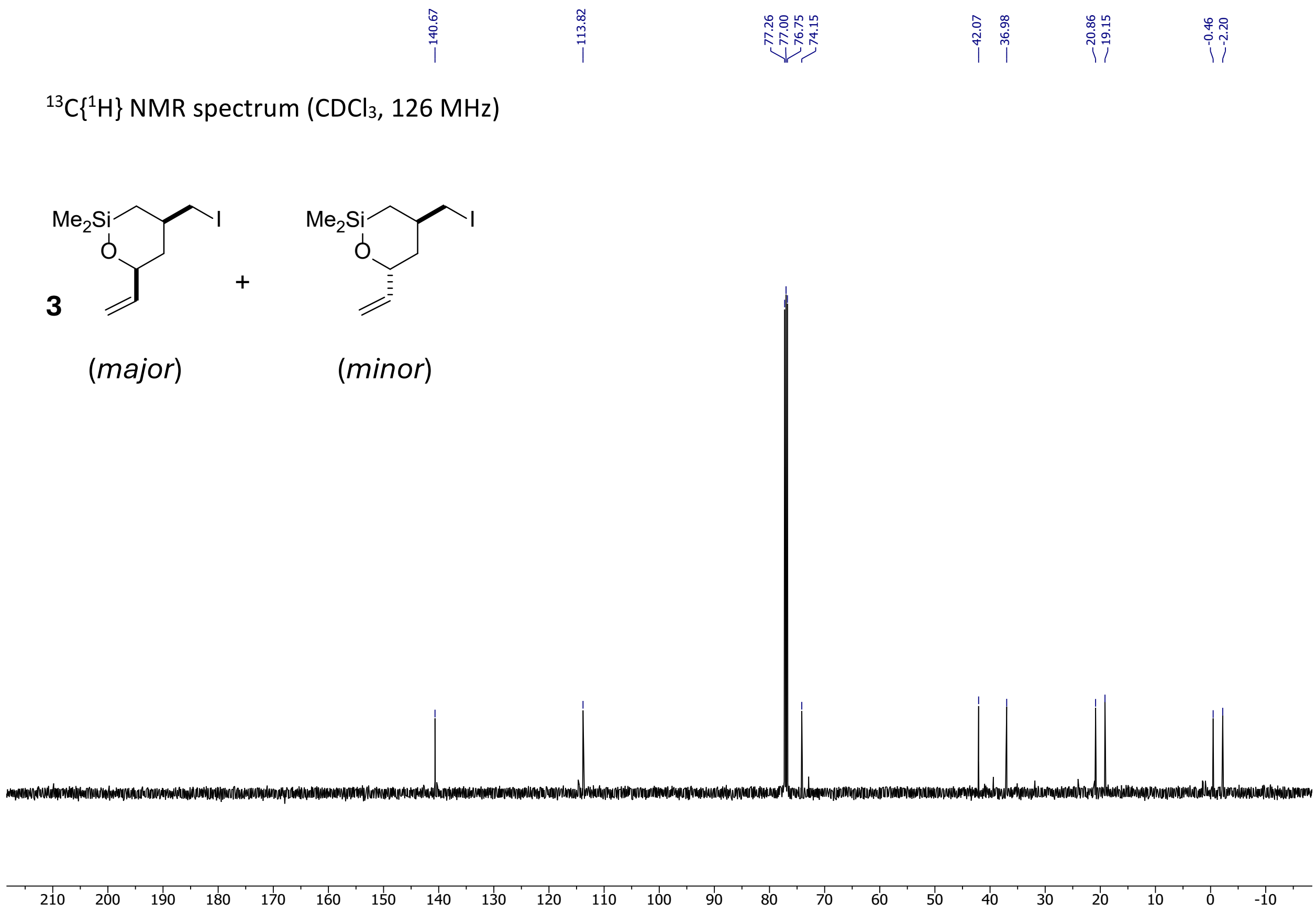
140.67
113.82
77.26 77.00 76.75 74.15
42.07
36.98
20.86 19.15
-0.46 -2.20
^{13}C{^{1}H} NMR spectrum (CDCl$_3$, 126 MHz)
Me$_2$Si
O
I
3
+
(*major*)
(*minor*)
210 200 190 180 170 160 150 140 130 120 110 100 90 80 70 60 50 40 30 20 10 0 -10

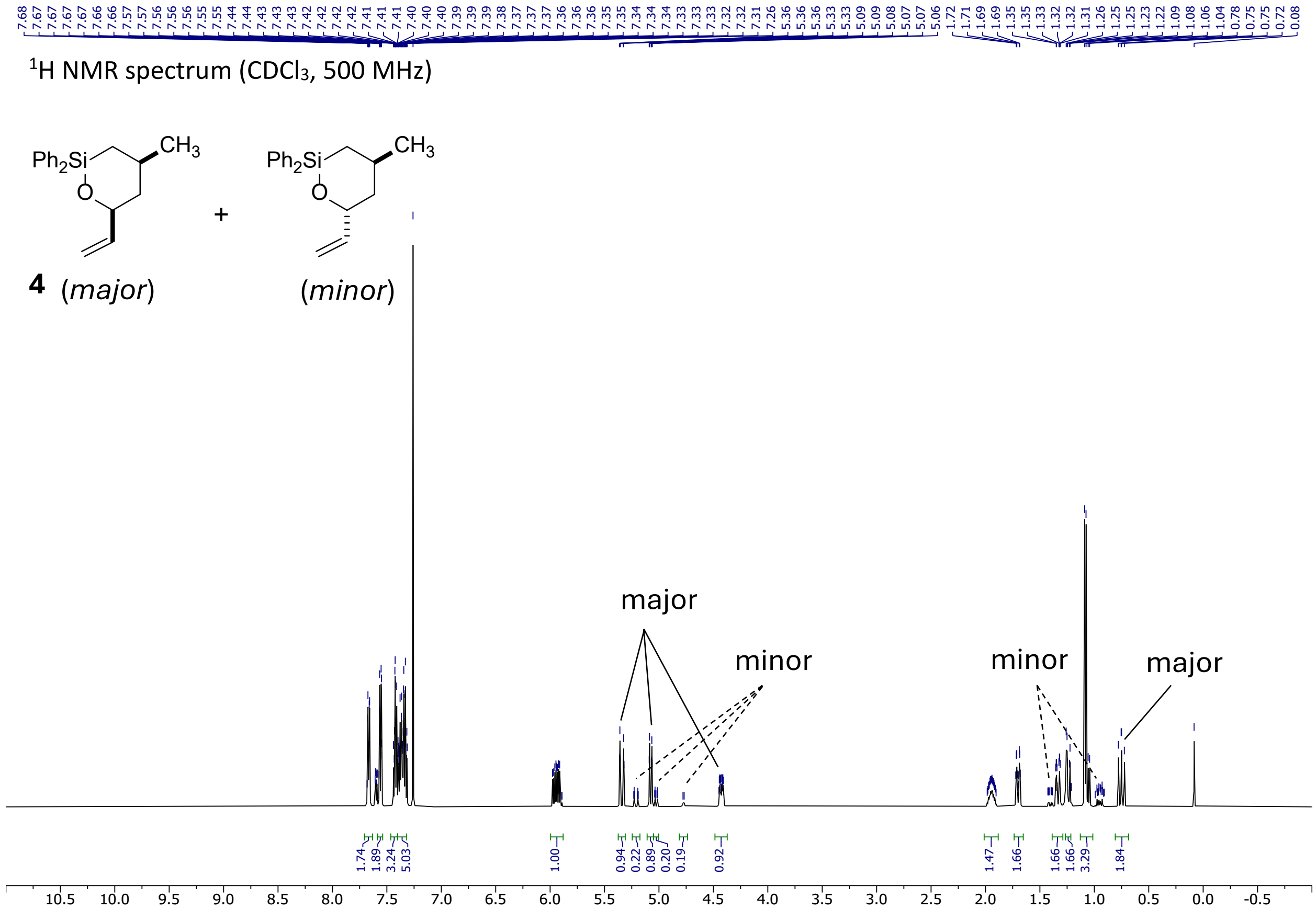

^{1}H NMR spectrum (CDCl$_3$, 500 MHz)
Ph$_2$Si
CH$_3$
O
4 (*major*)
(*minor*)
major
minor
minor
major

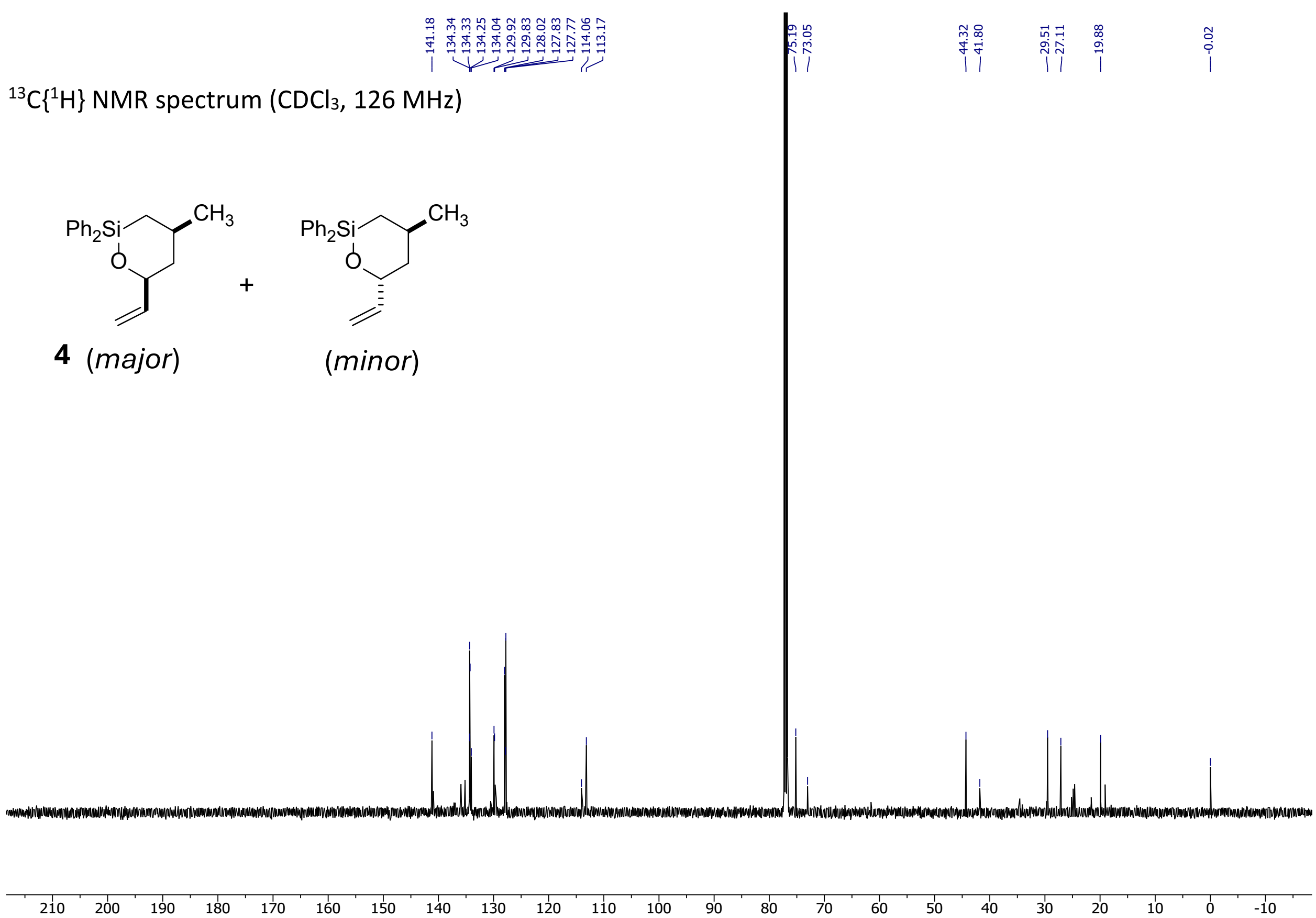

^{13}C{^{1}H} NMR spectrum (CDCl$_3$, 126 MHz)
Ph$_2$Si
CH$_3$
O
4 (*major*)
(*minor*)

^{1}H NMR spectrum ($CDCl_3$, 500 MHz)

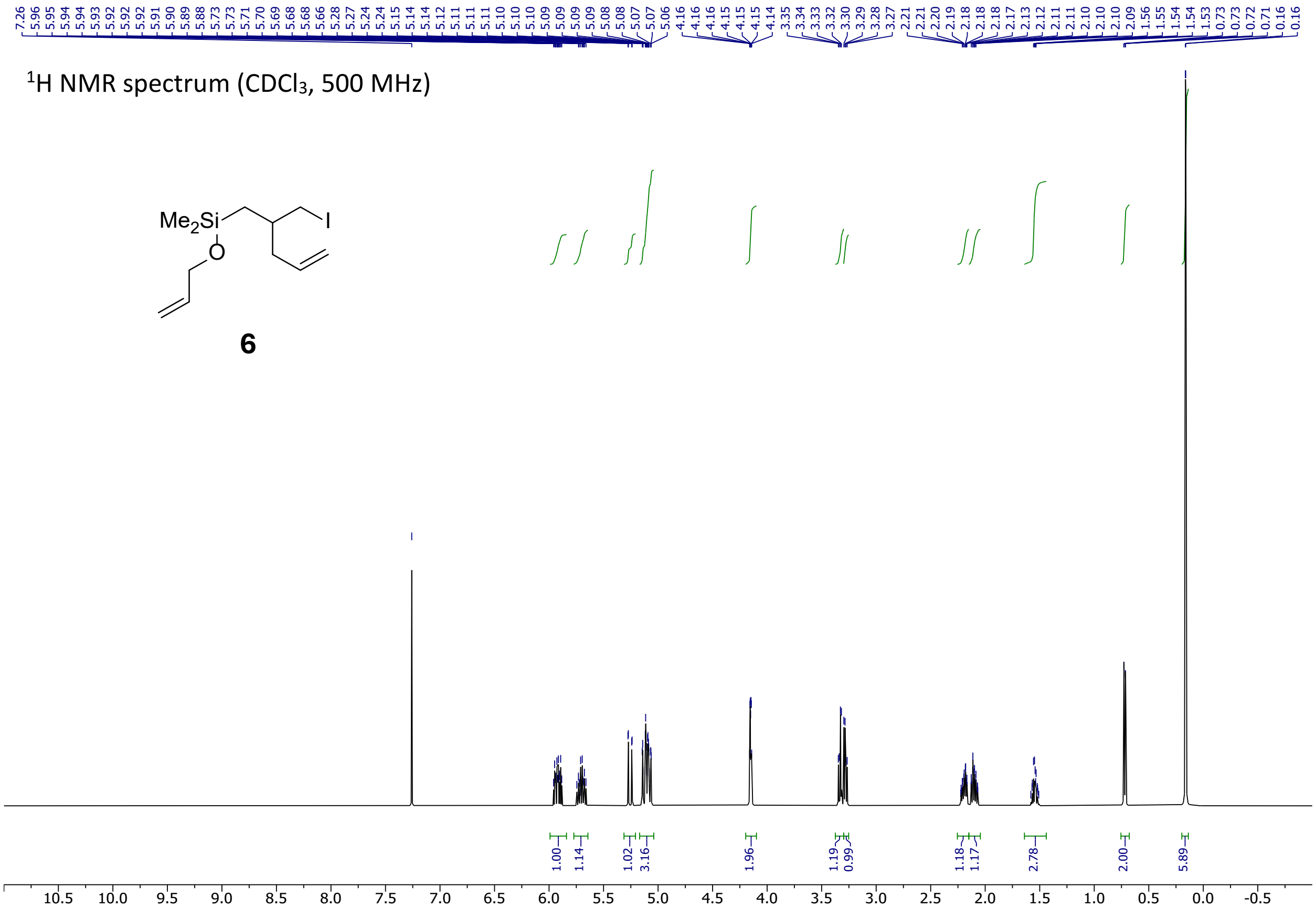

^{13}C{^{1}H} NMR spectrum ($CDCl_3$, 126 MHz)

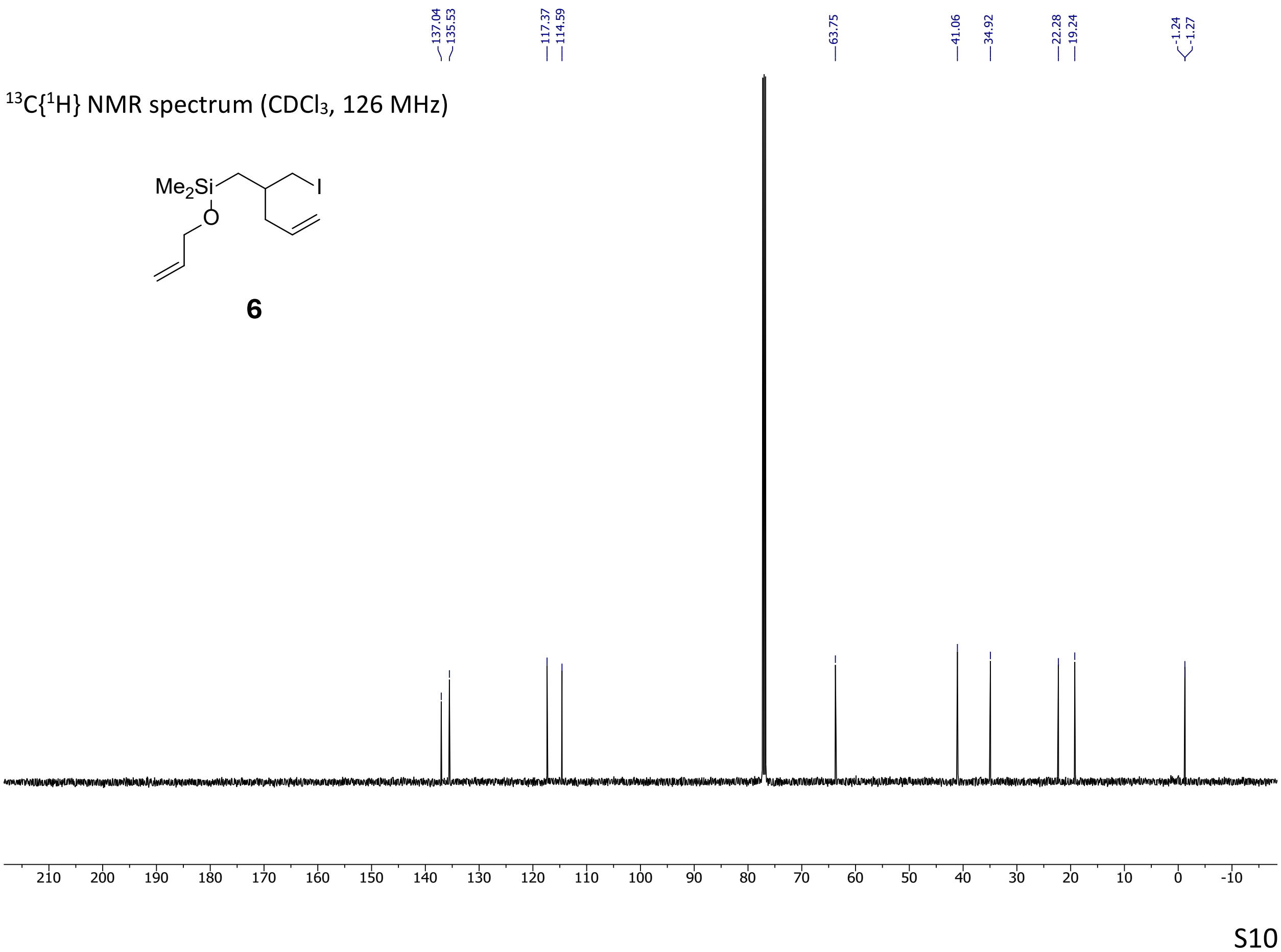

^{1}H NMR spectrum ($CDCl_3$, 500 MHz)

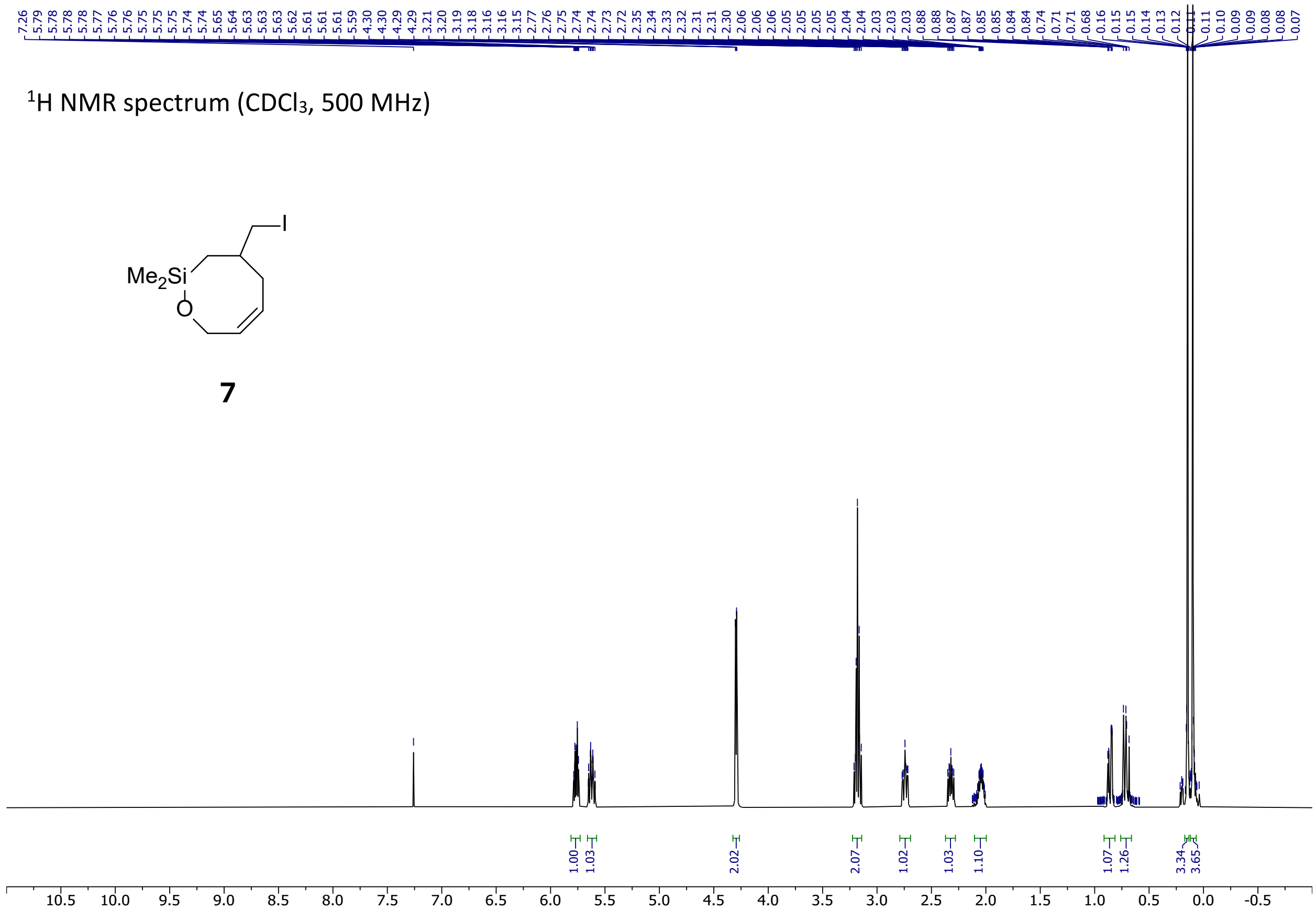

^{13}C{^{1}H} NMR spectrum ($CDCl_3$, 126 MHz)

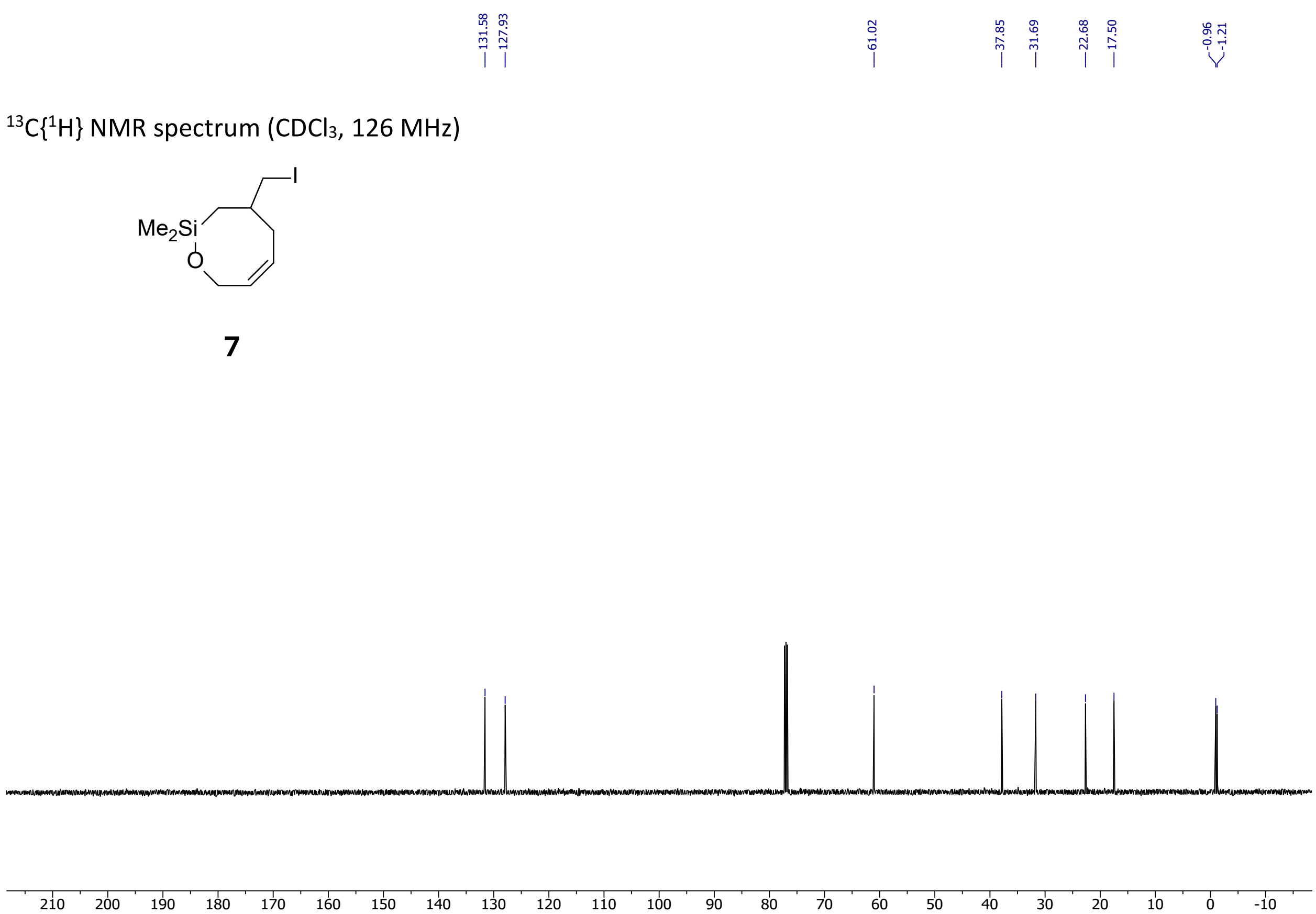

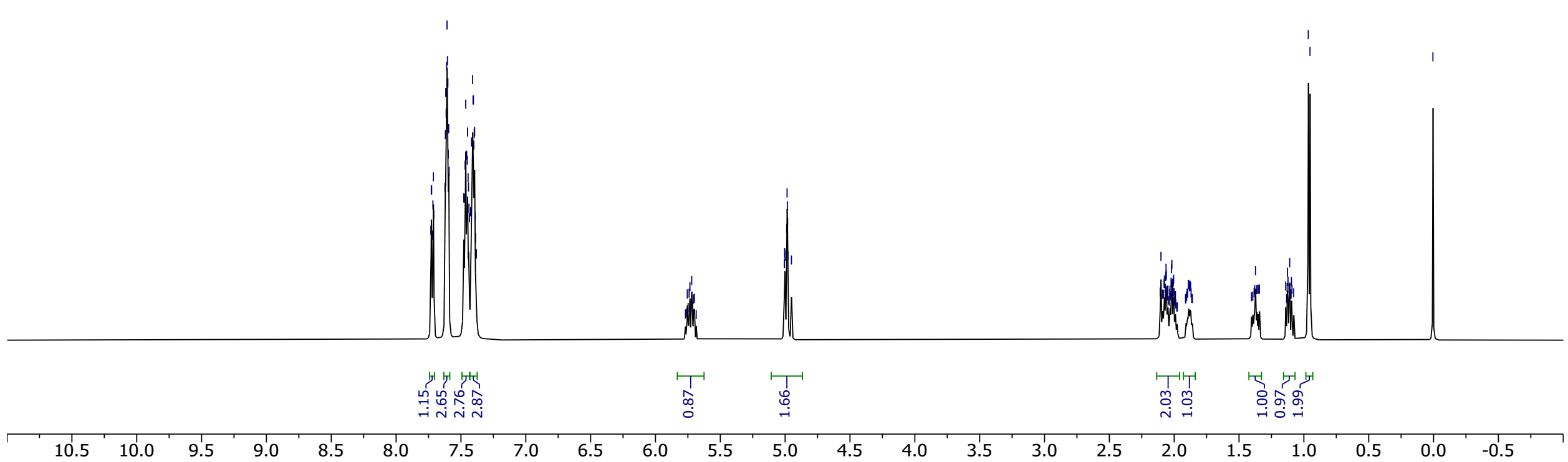

^{1}H NMR spectrum ($CDCl_3$, 500 MHz)

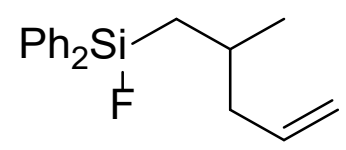

8

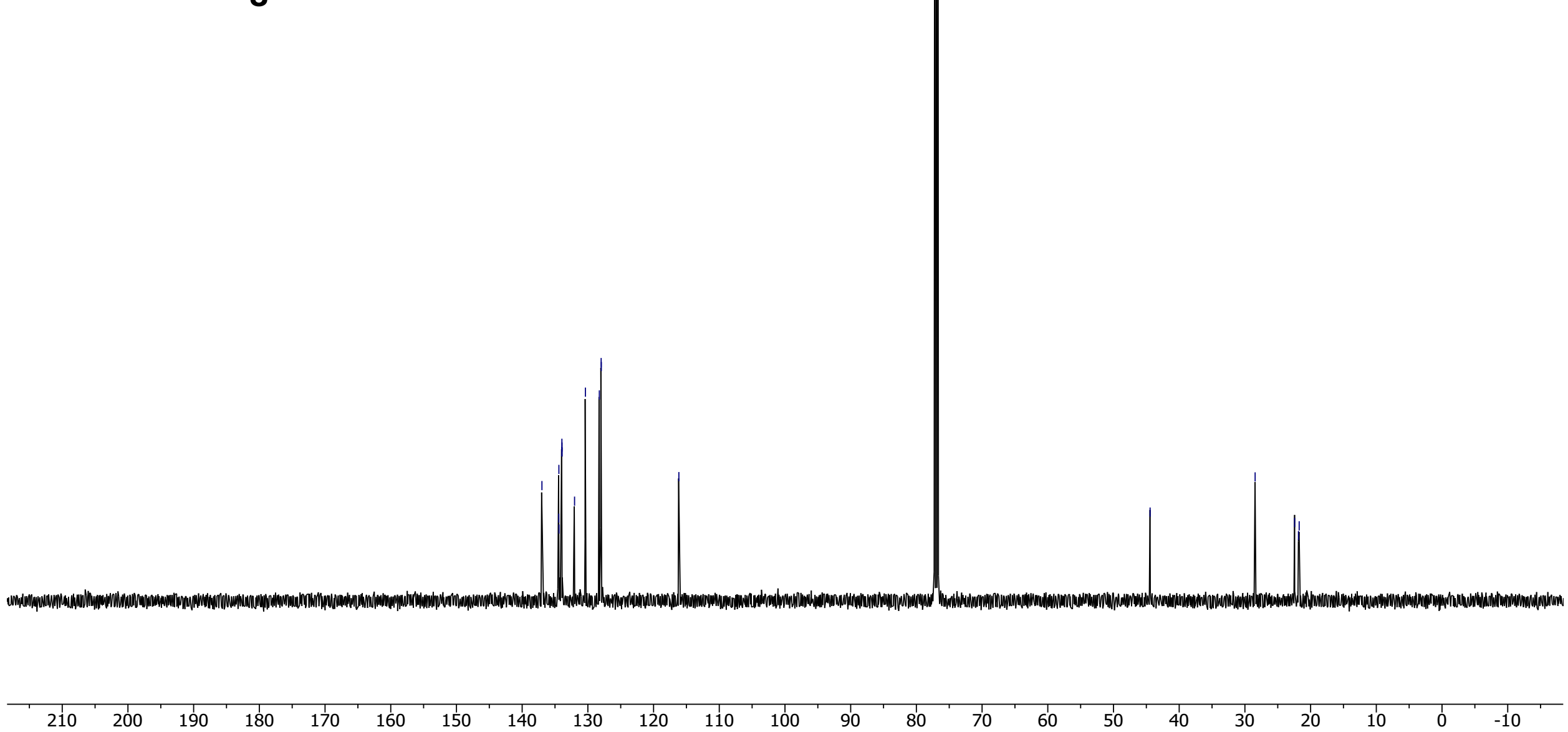

$^{13}C\{^1H\}$ NMR spectrum ($CDCl_3$, 126 MHz)

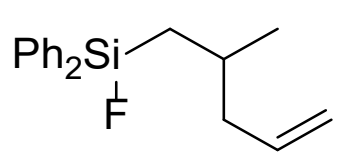

8

^{1}H NMR spectrum ($CDCl_3$, 500 MHz)

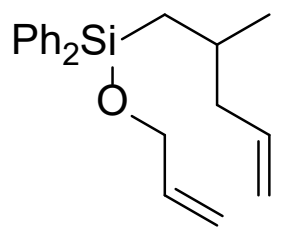

9

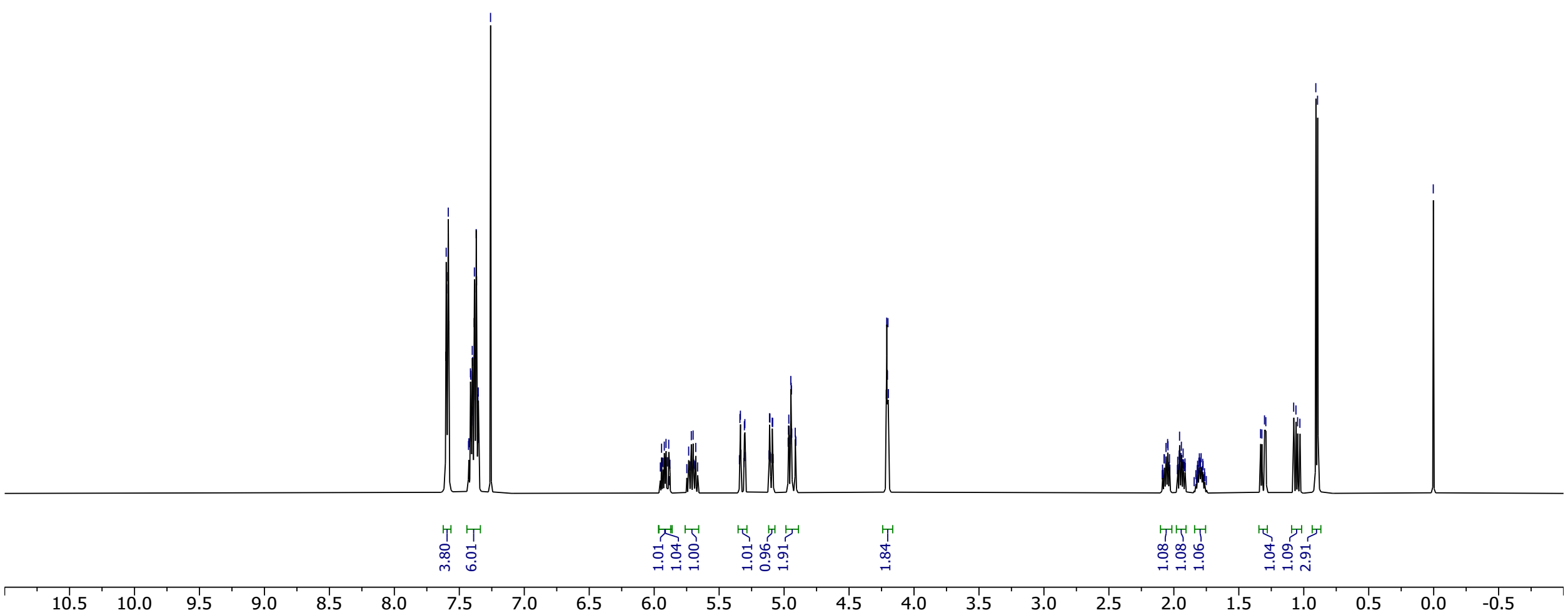

^{13}C{^{1}H} NMR spectrum ($CDCl_3$, 126 MHz)

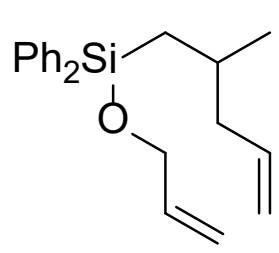

9

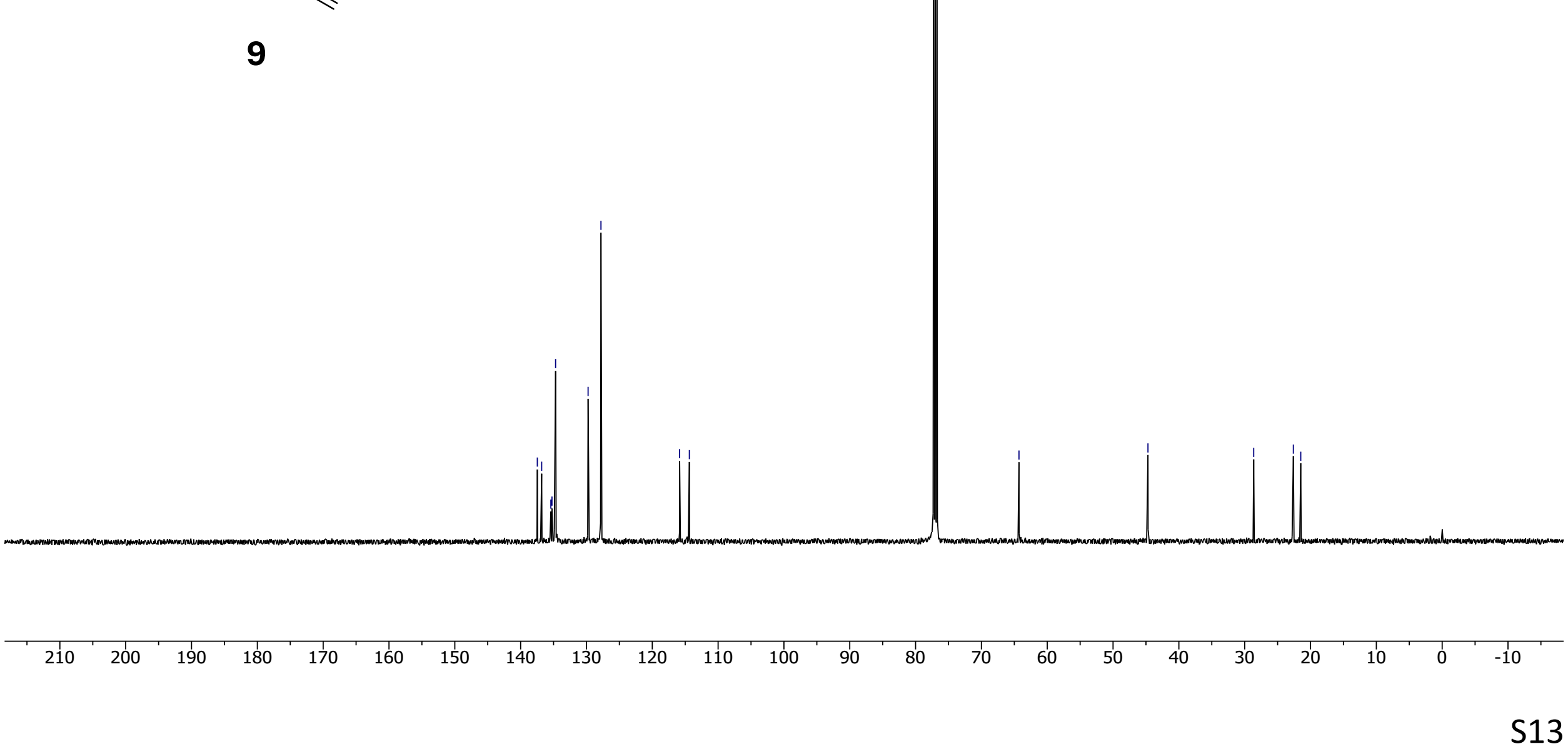

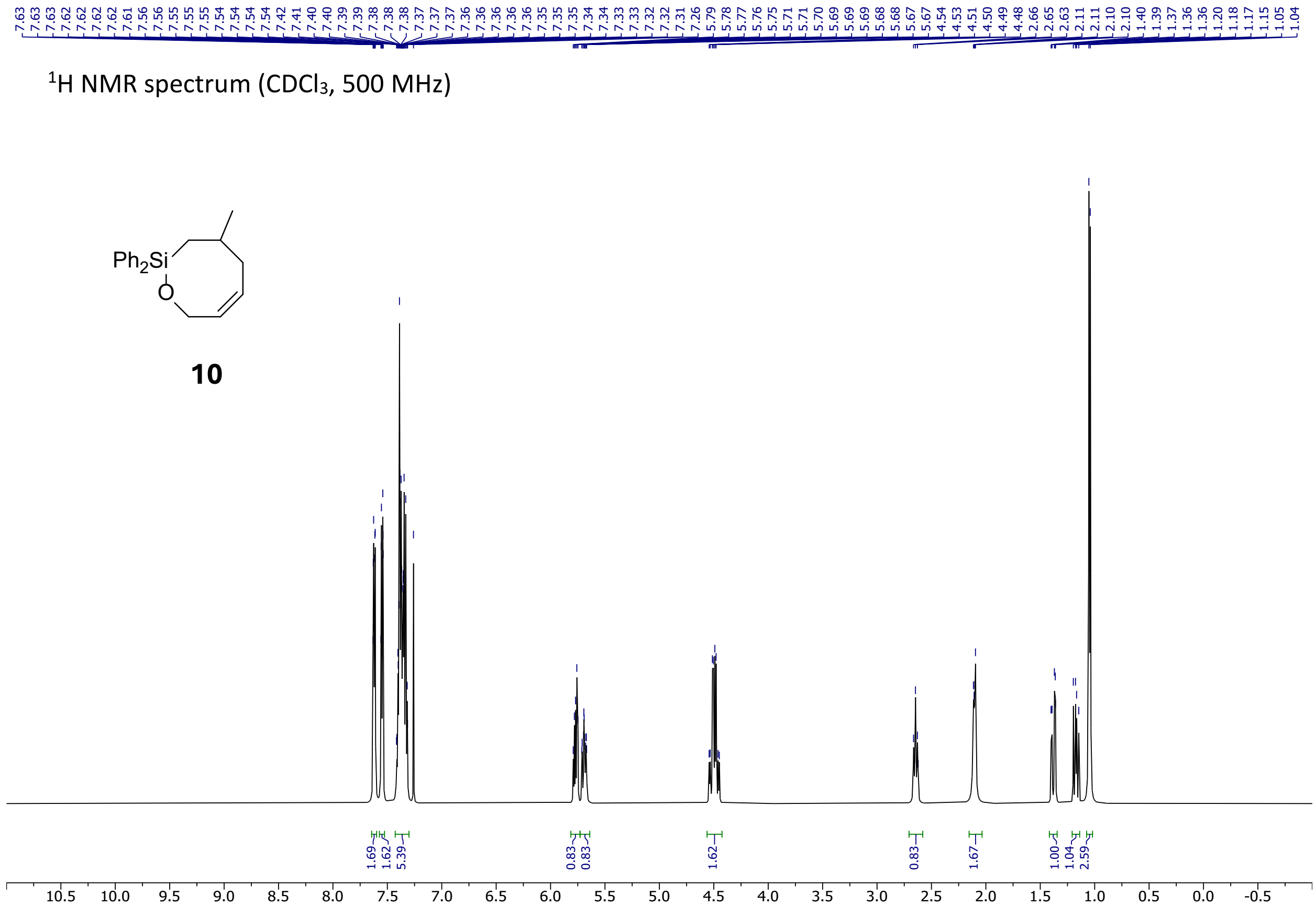

¹H NMR spectrum (CDCl₃, 500 MHz)
Ph2Si
O
10

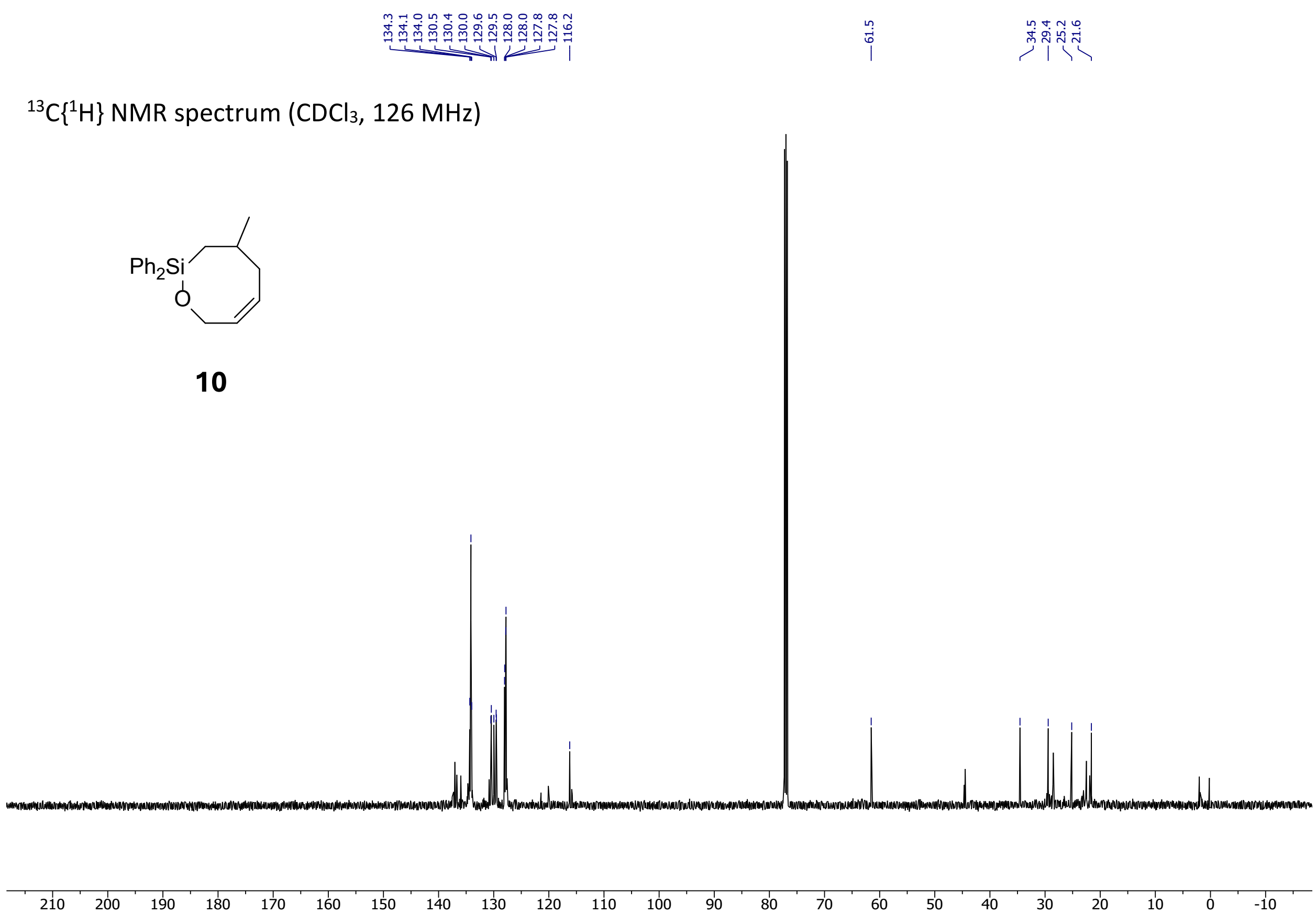

¹³C{¹H} NMR spectrum (CDCl₃, 126 MHz)
Ph2Si
O
10

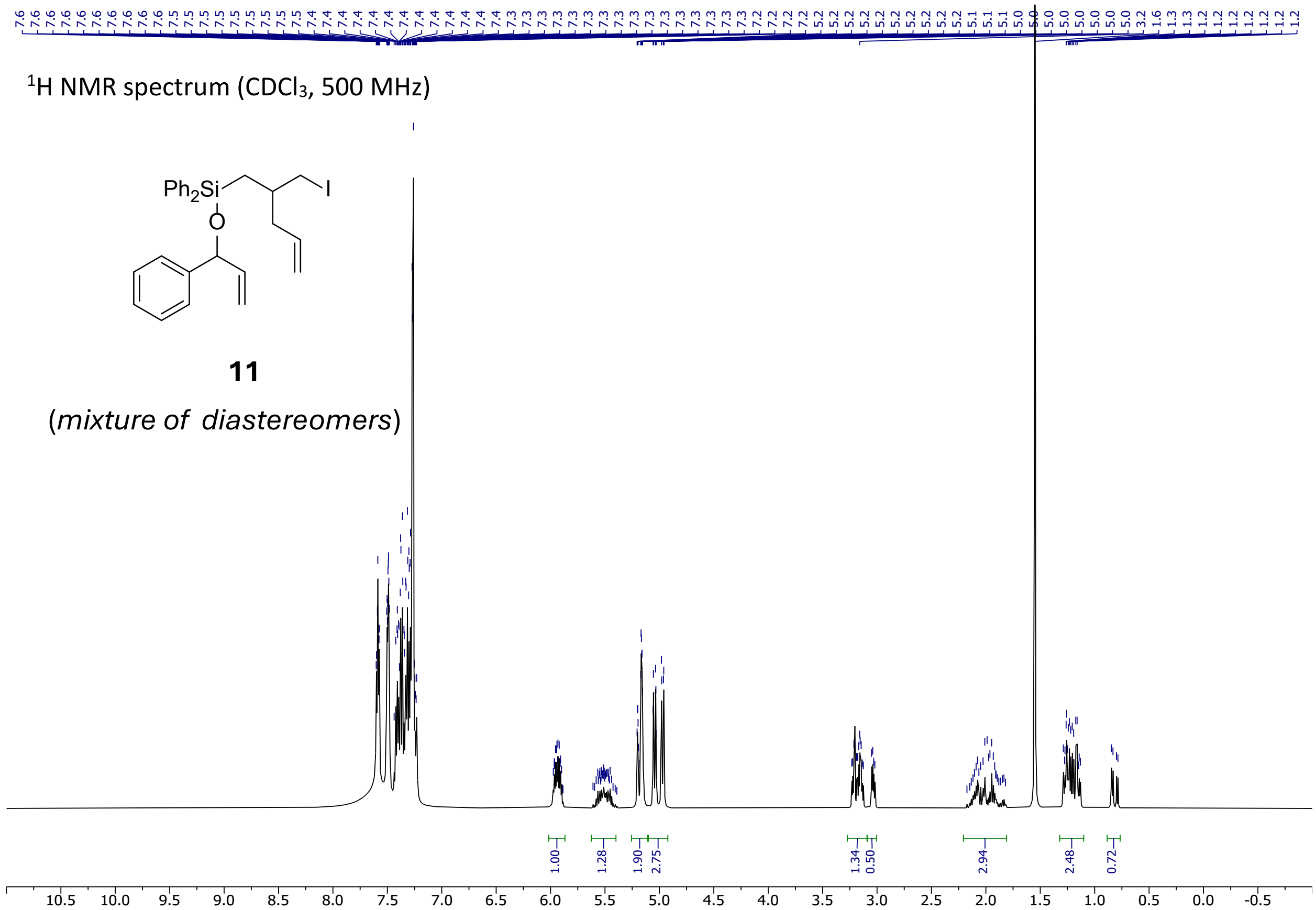

1H NMR spectrum (CDCl3, 500 MHz)
Ph2Si
O
I
11
(mixture of diastereomers)

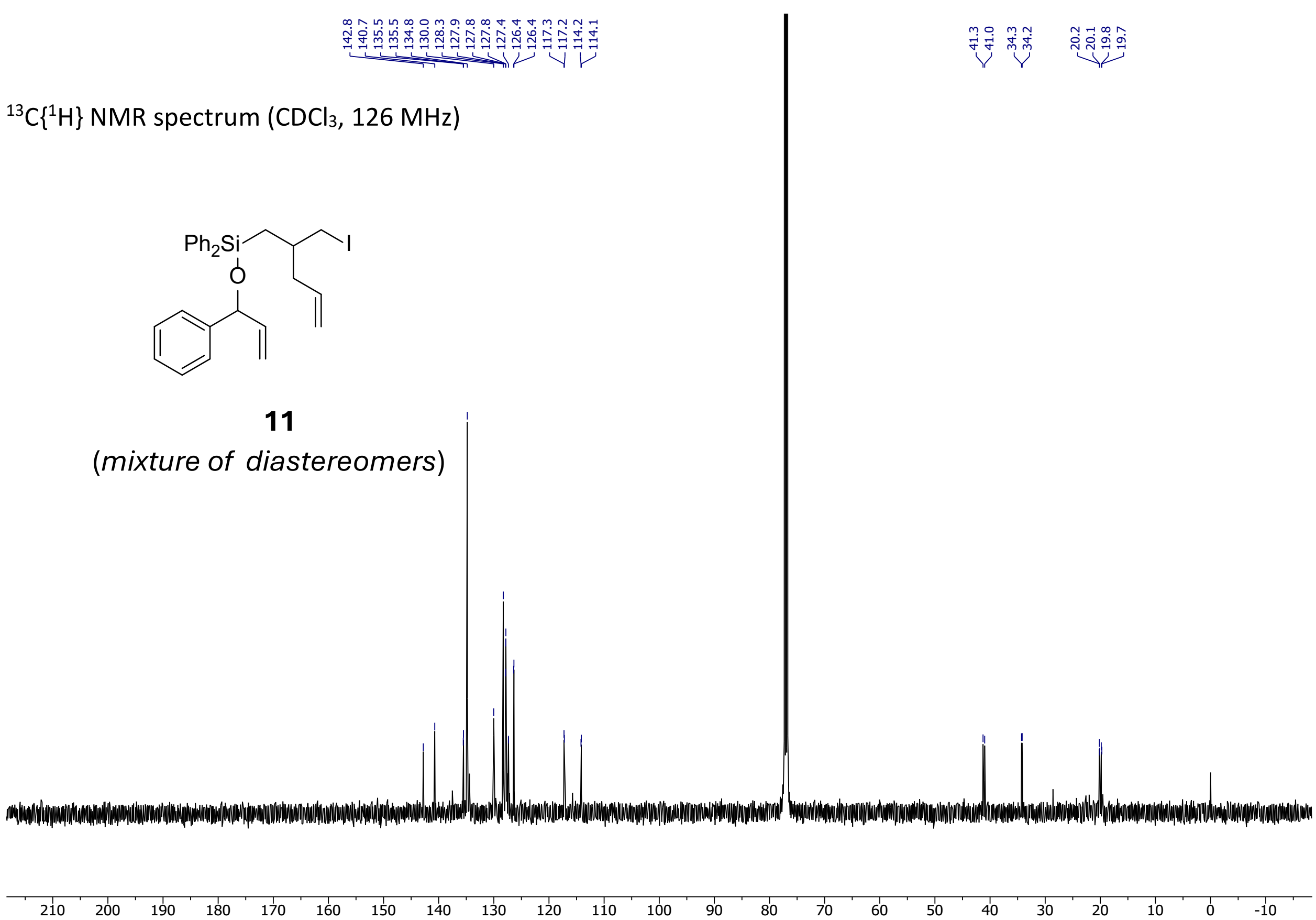

13C{1H} NMR spectrum (CDCl3, 126 MHz)
Ph2Si
O
I
11
(mixture of diastereomers)

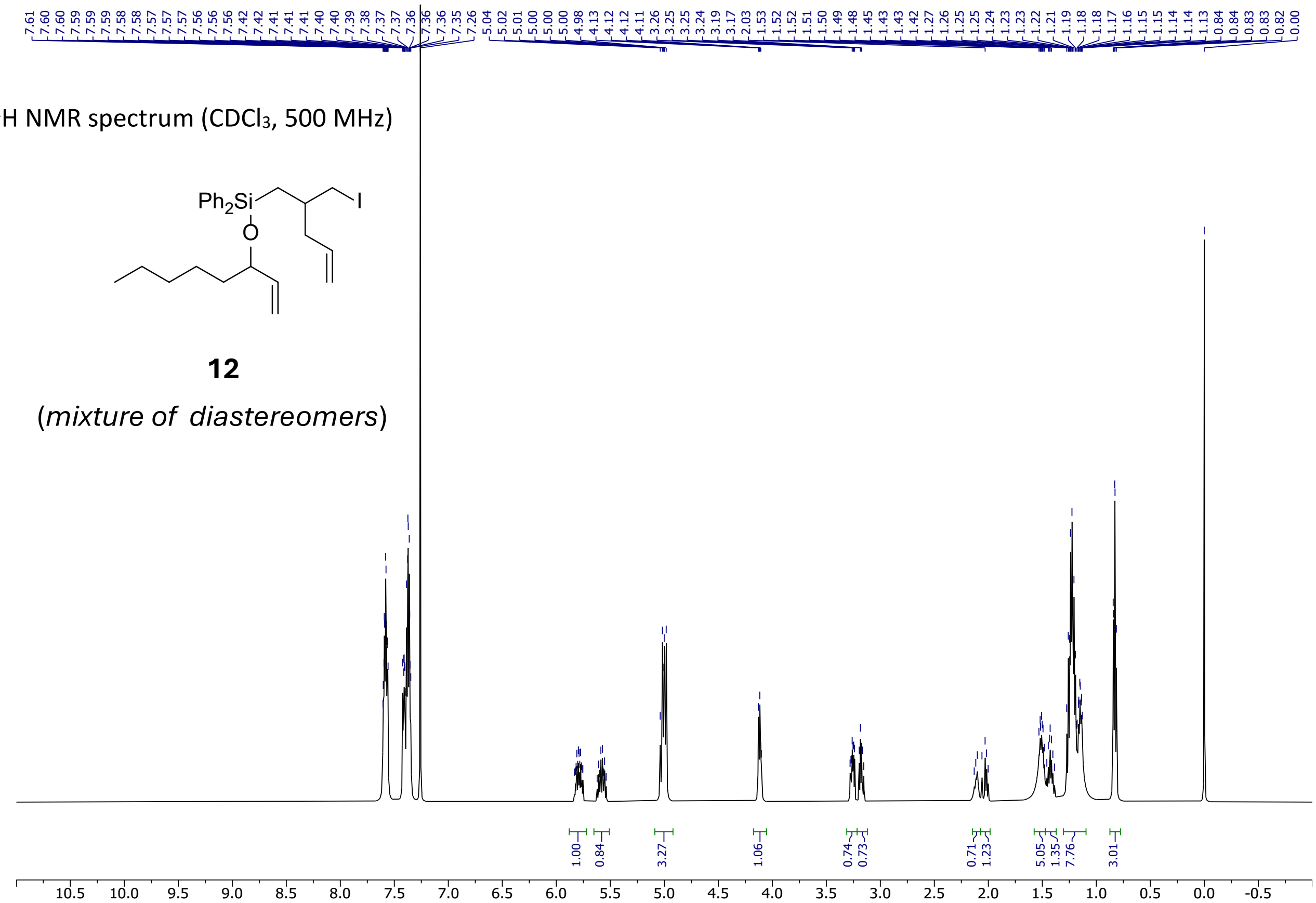

^{1}H NMR spectrum ($CDCl_3$, 500 MHz)

12

(*mixture of diastereomers*)

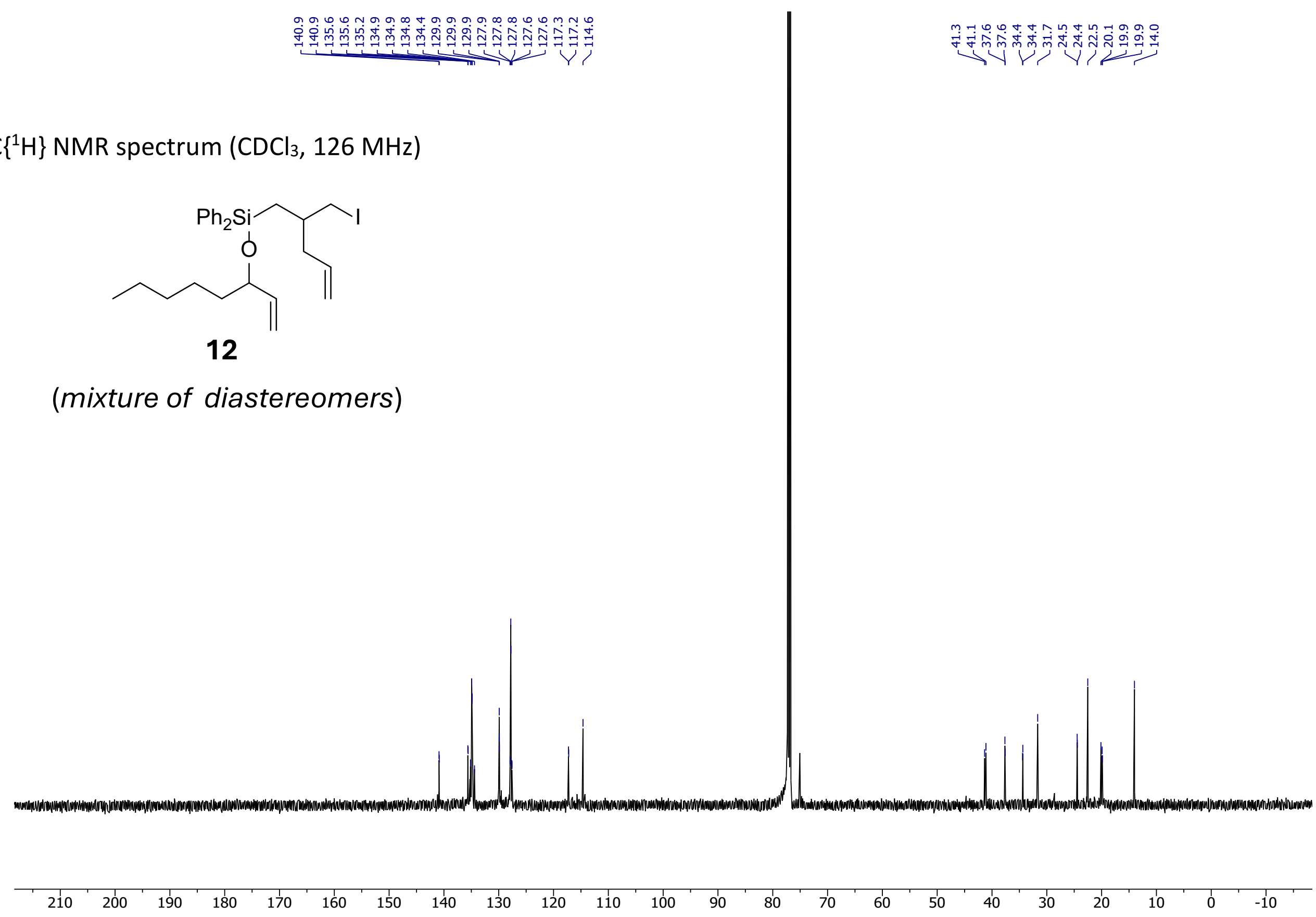

^{13}C{^{1}H} NMR spectrum ($CDCl_3$, 126 MHz)

12

(*mixture of diastereomers*)

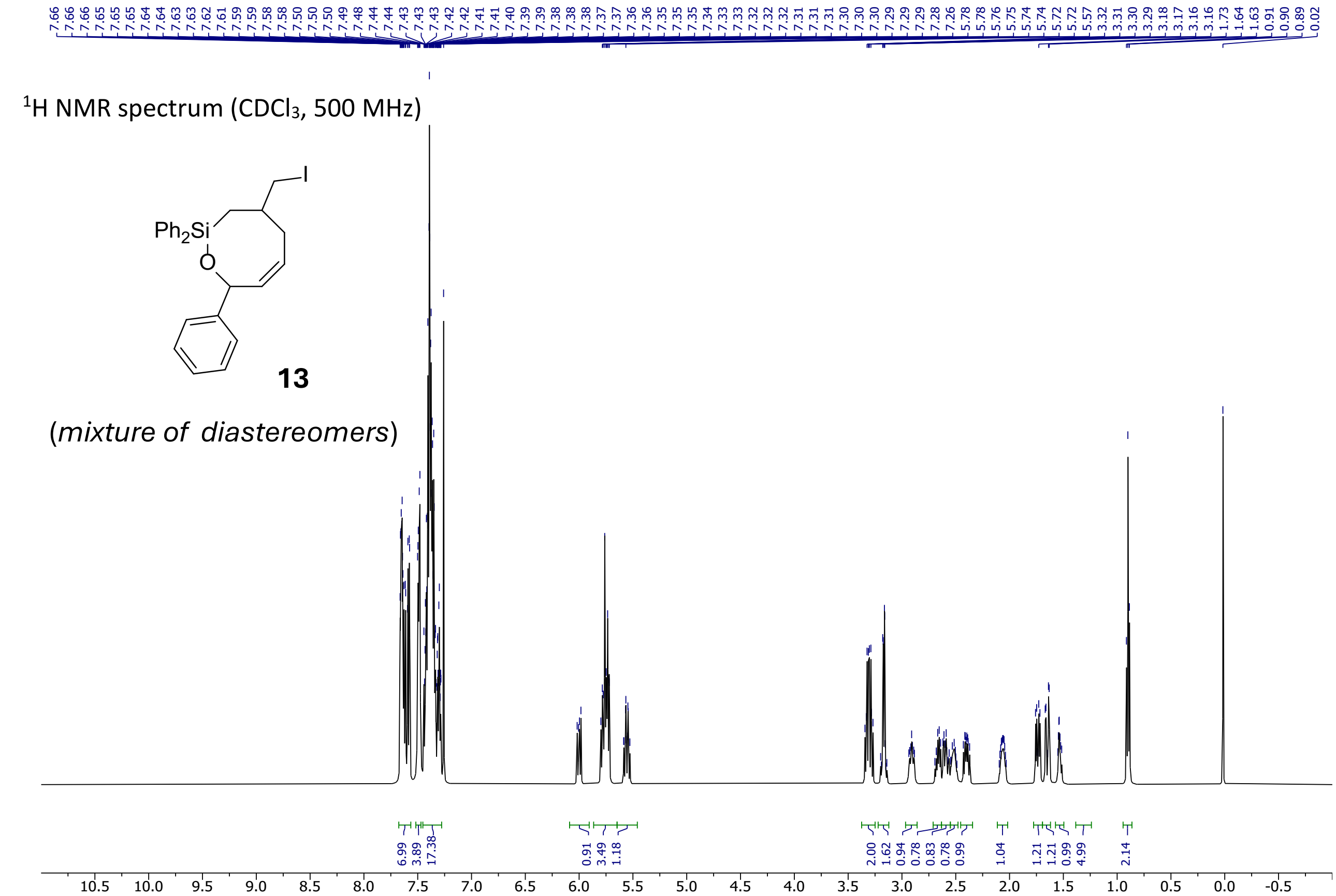

^{1}H NMR spectrum (CDCl$_3$, 500 MHz)
Ph$_2$Si
O
I
13
(mixture of diastereomers)

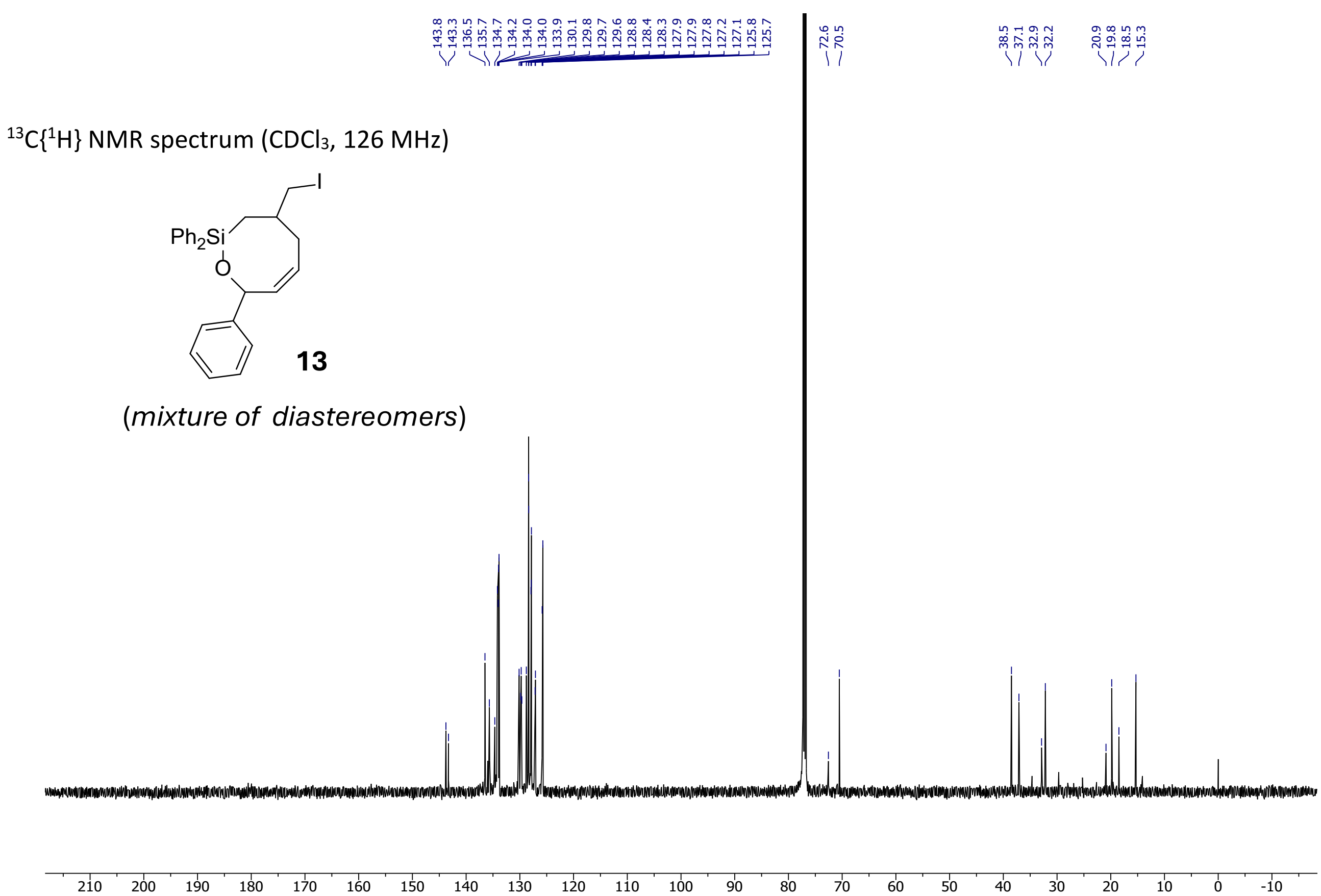

^{13}C{^{1}H} NMR spectrum (CDCl$_3$, 126 MHz)
Ph$_2$Si
O
I
13
(mixture of diastereomers)

^{1}H NMR spectrum ($CDCl_3$, 500 MHz)

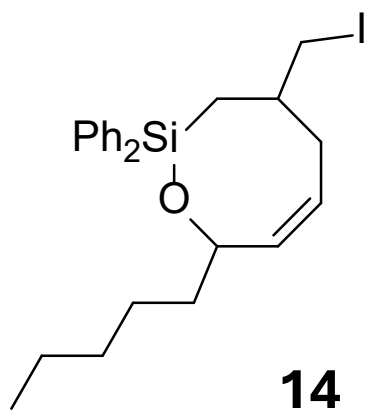

14

(*mixture of diastereomers*)

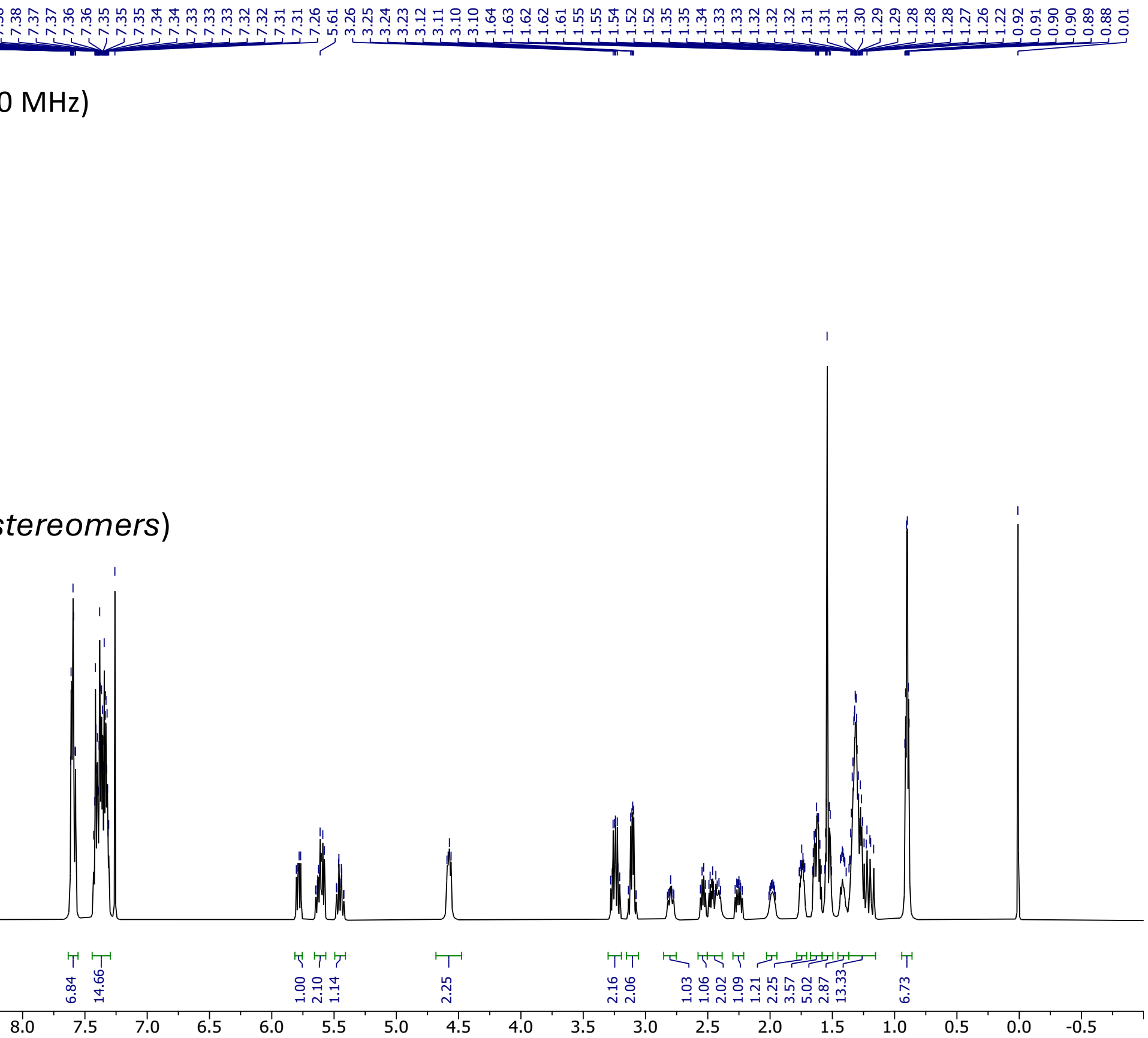

$^{13}C\{^{1}H\}$ NMR spectrum ($CDCl_3$, 126 MHz)

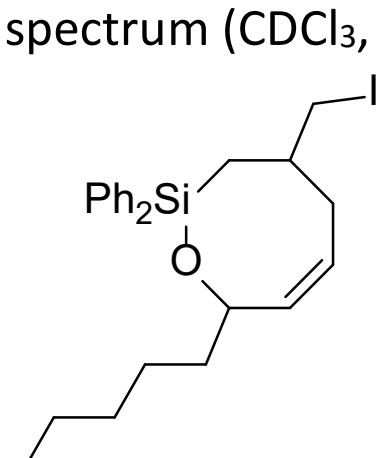

14

(*mixture of diastereomers*)

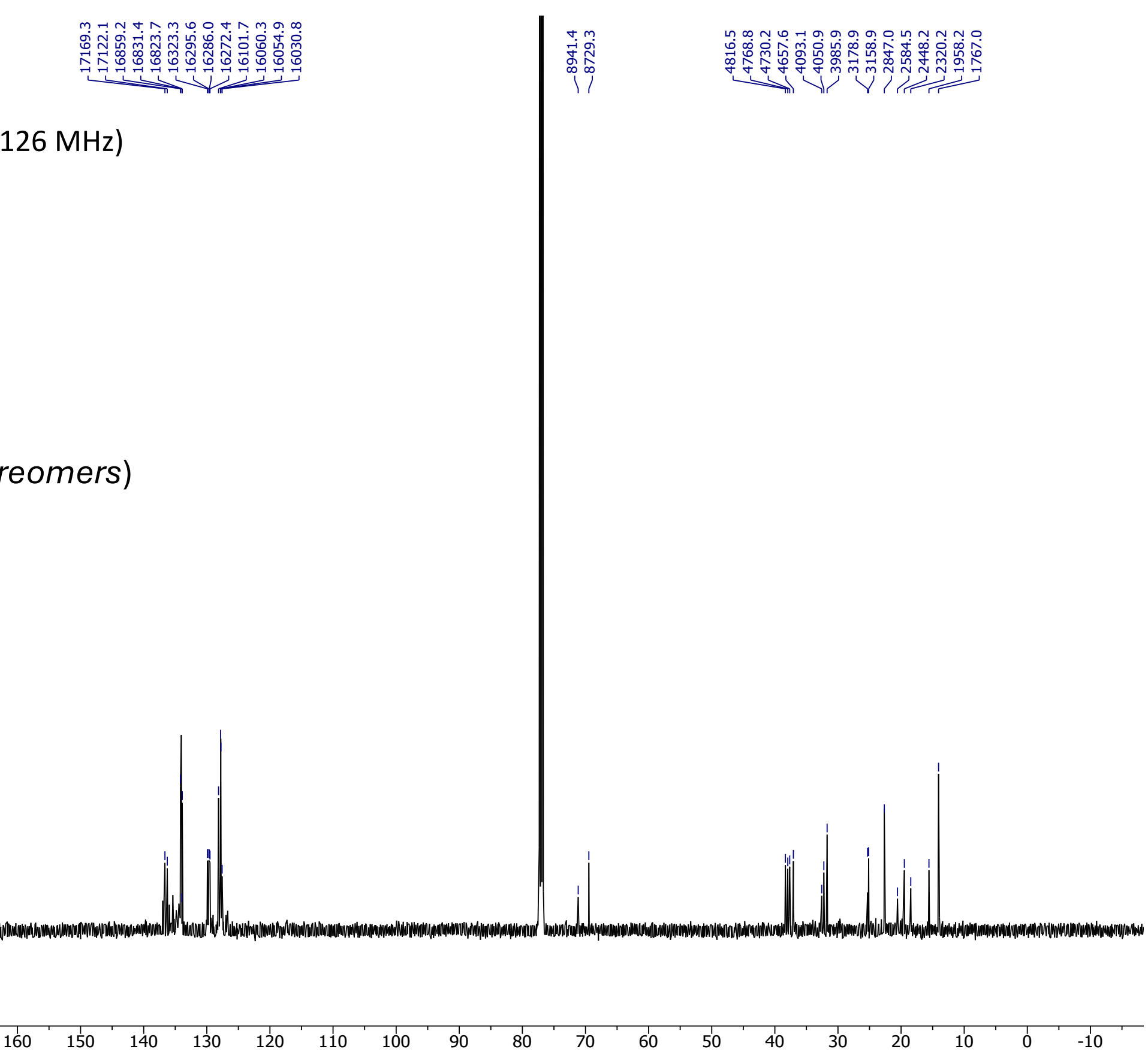

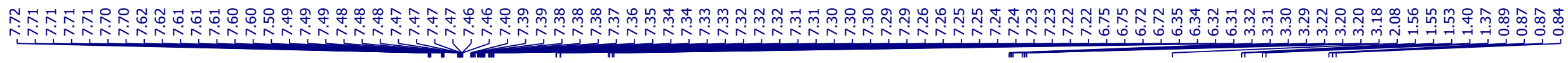

^{1}H NMR spectrum ($CDCl_3$, 500 MHz)

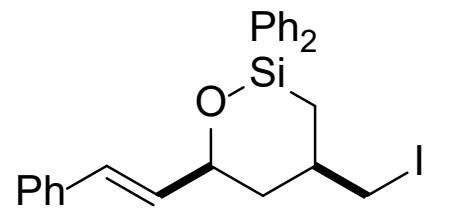

15

(*major diastereomer*)

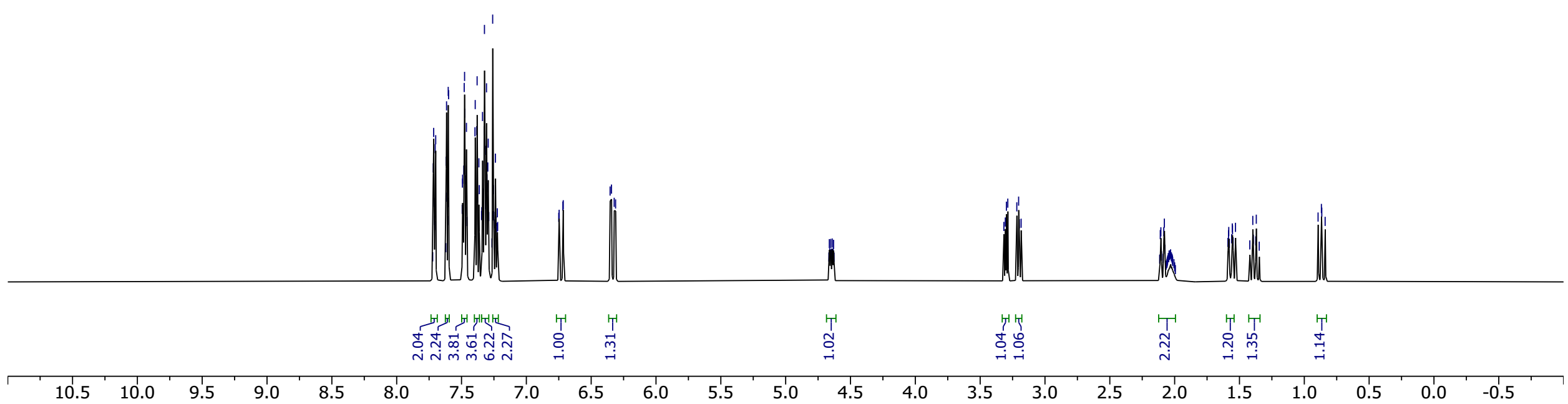

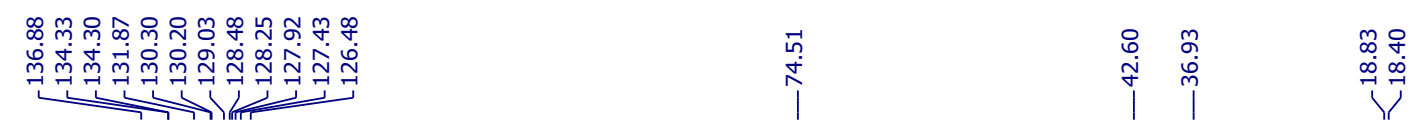

$^{13}C\{^{1}H\}$ NMR spectrum ($CDCl_3$, 126 MHz)

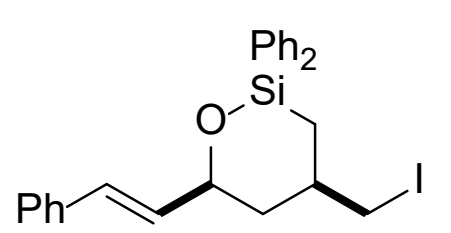

15

(*major diastereomer*)

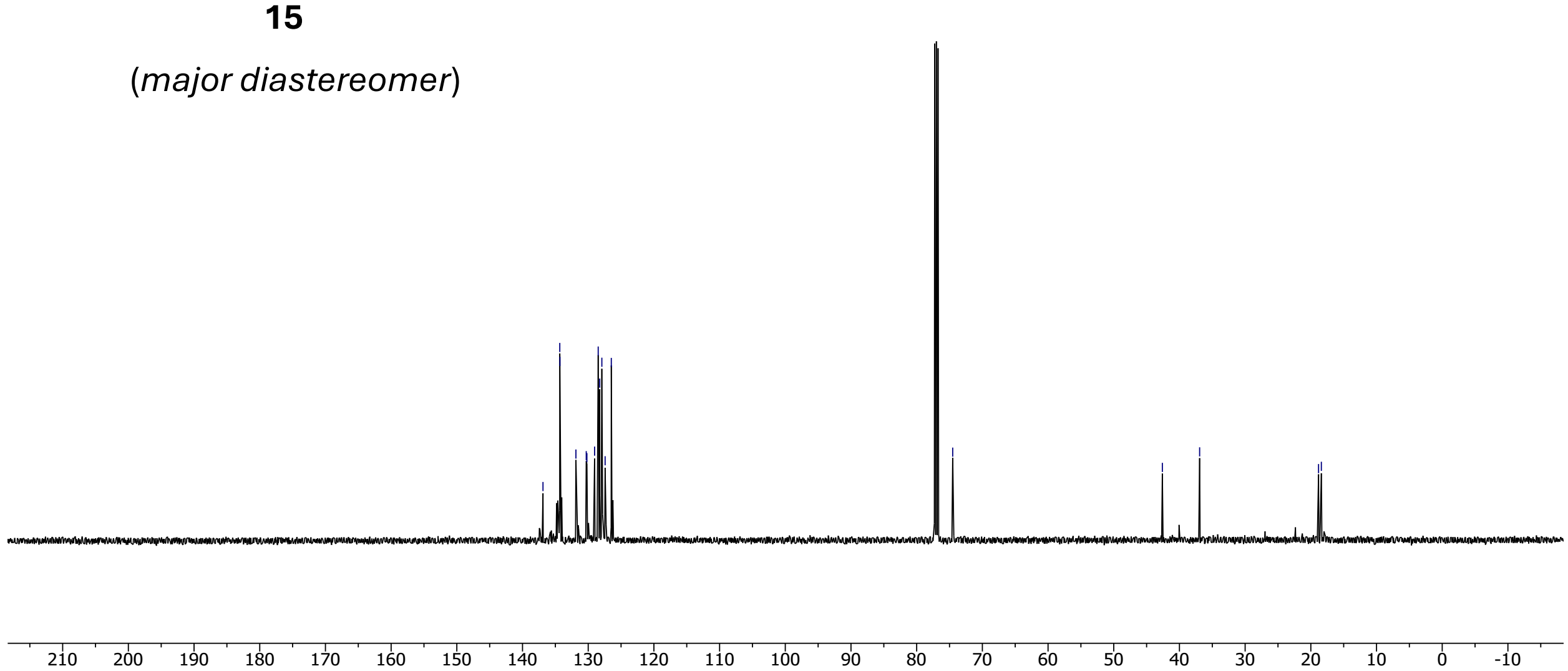

^{1}H NMR spectrum ($CDCl_3$, 500 MHz)

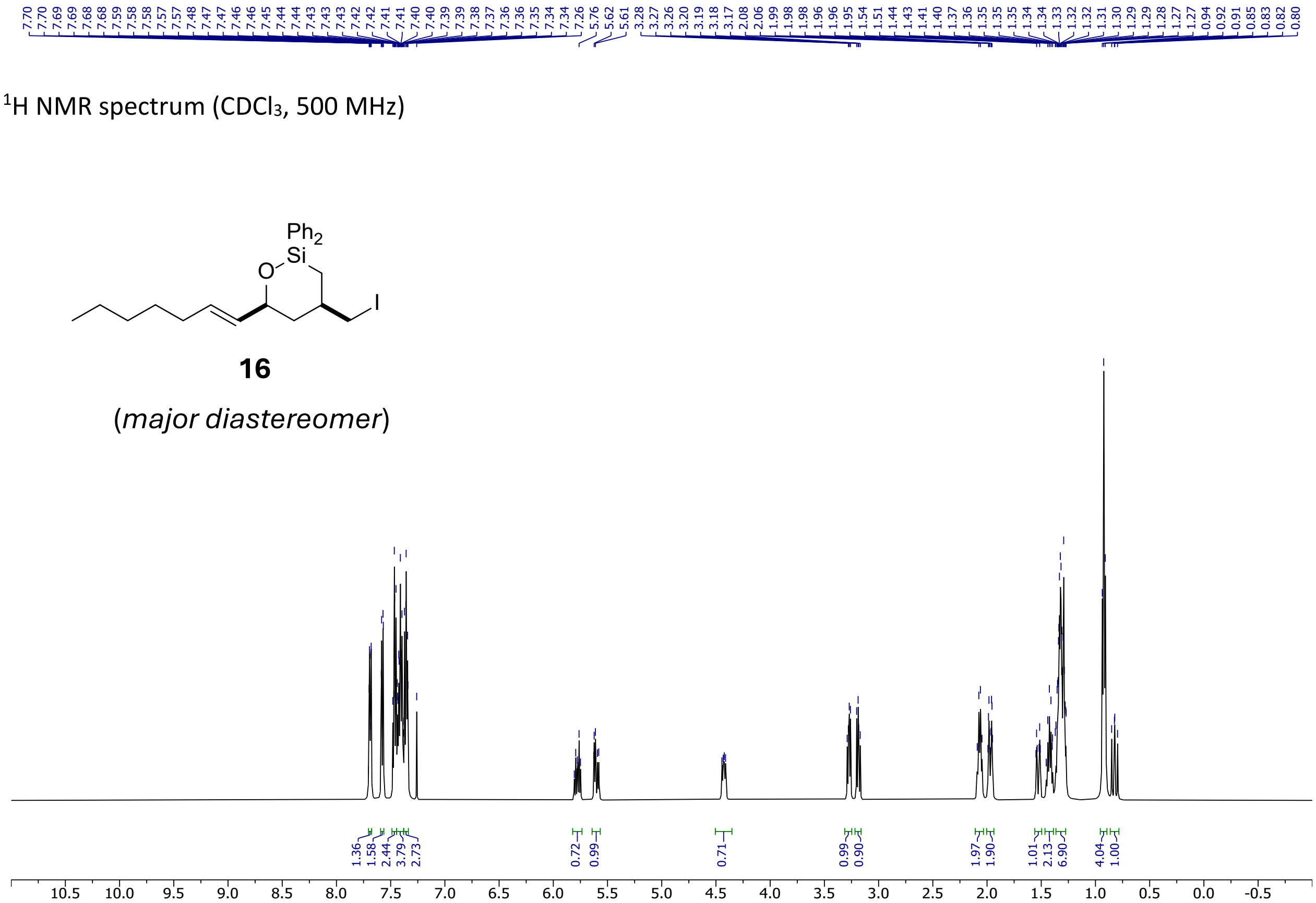

16

(*major diastereomer*)

$^{13}C\{^1H\}$ NMR spectrum ($CDCl_3$, 126 MHz)

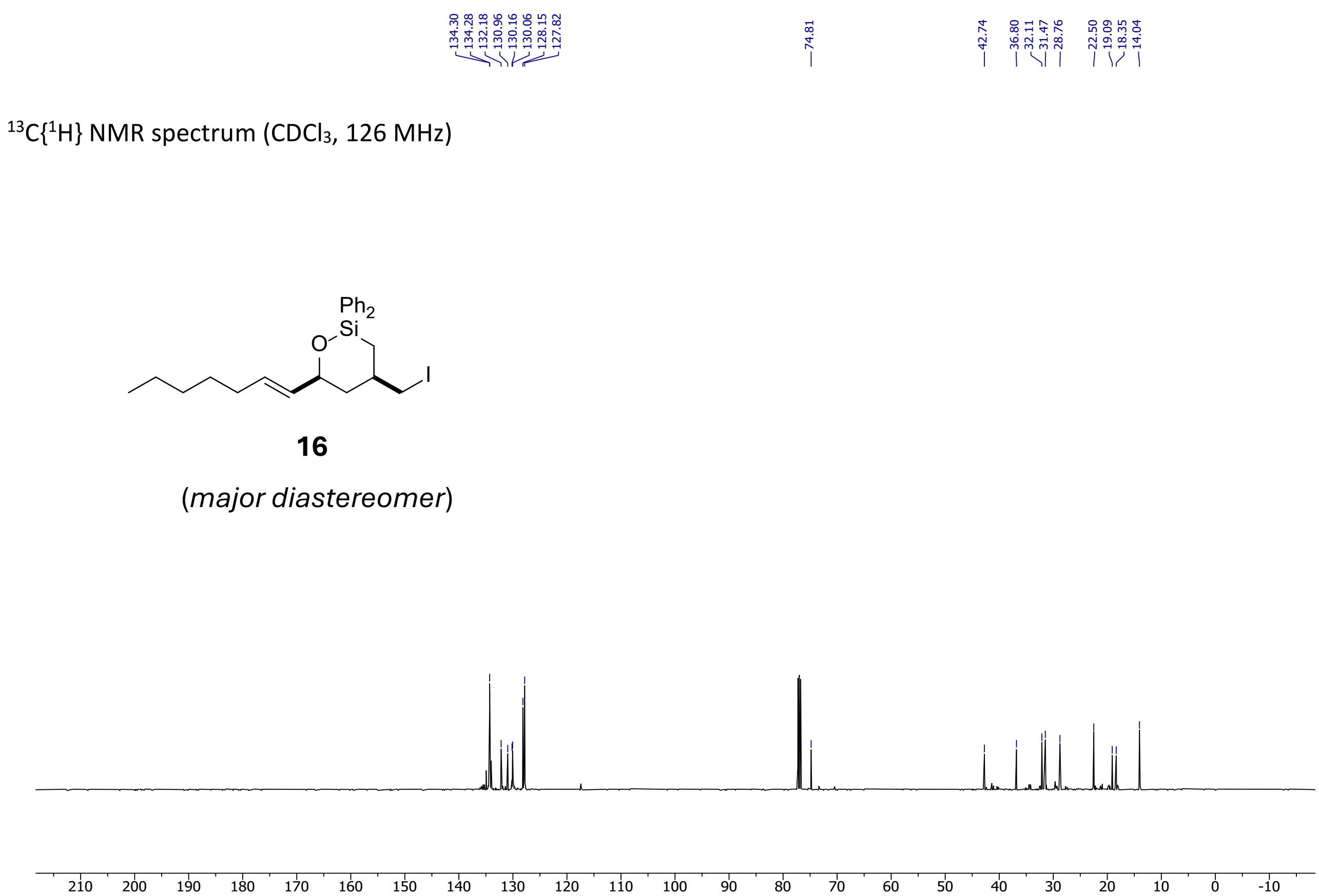

16

(*major diastereomer*)

^{1}H NMR spectrum ($CDCl_3$, 500 MHz)

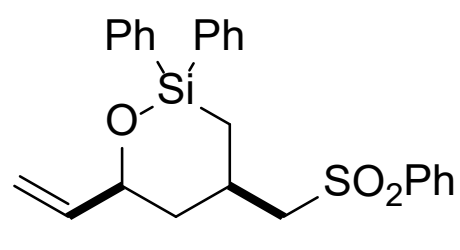

17

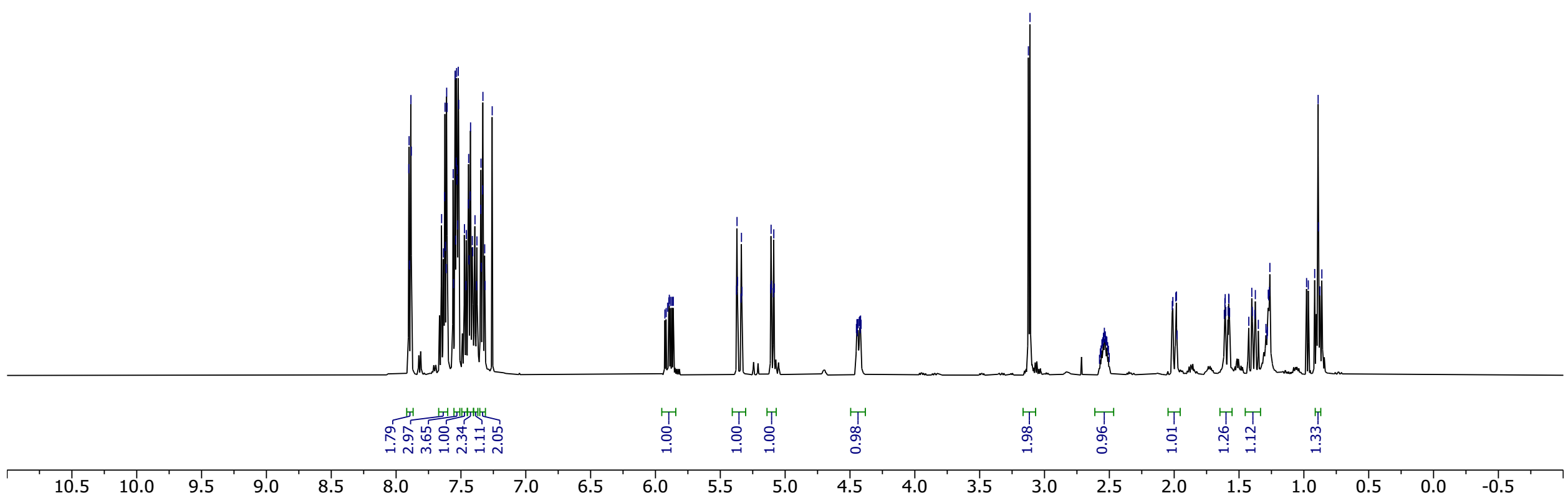

$^{13}C\{^{1}H\}$ NMR spectrum ($CDCl_3$, 126 MHz)

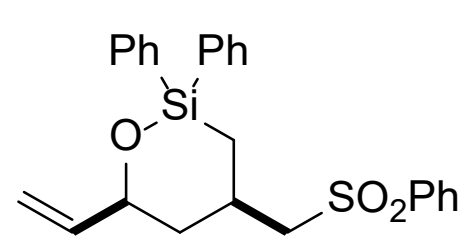

17

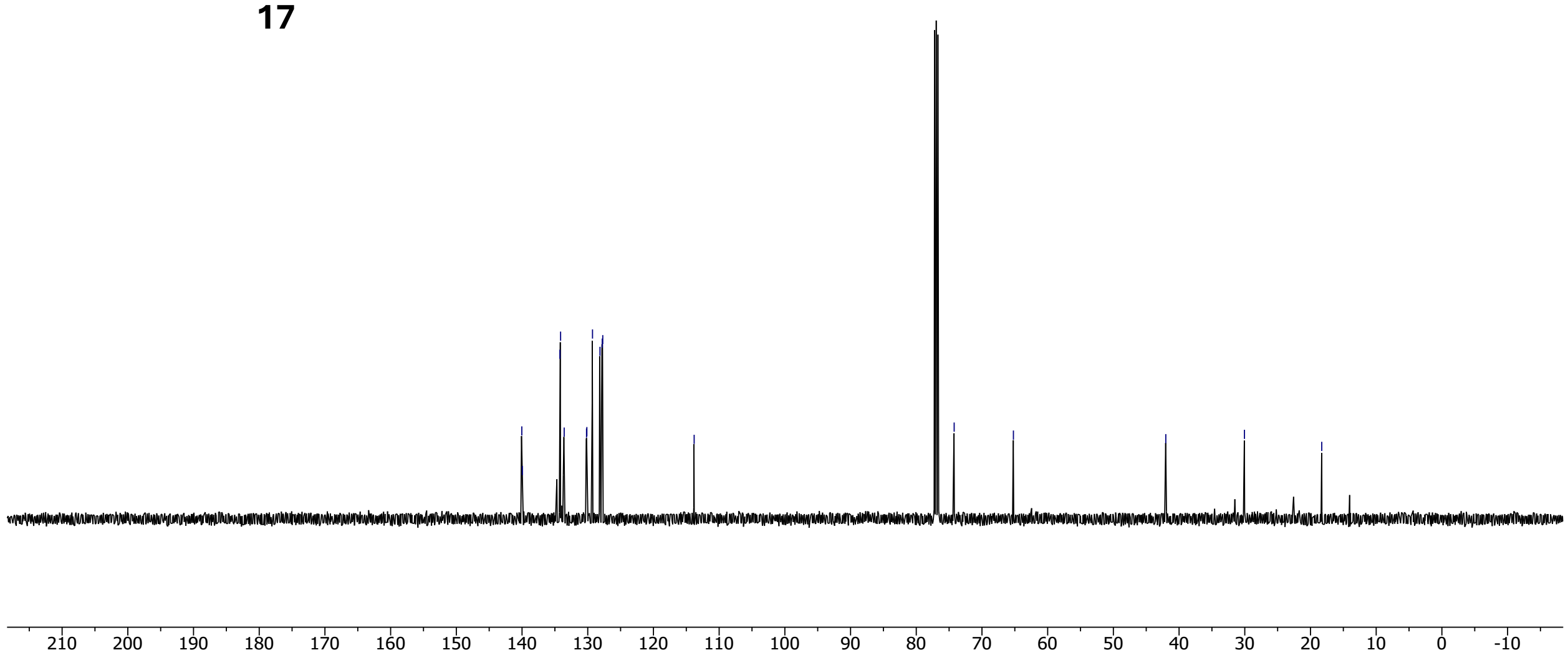

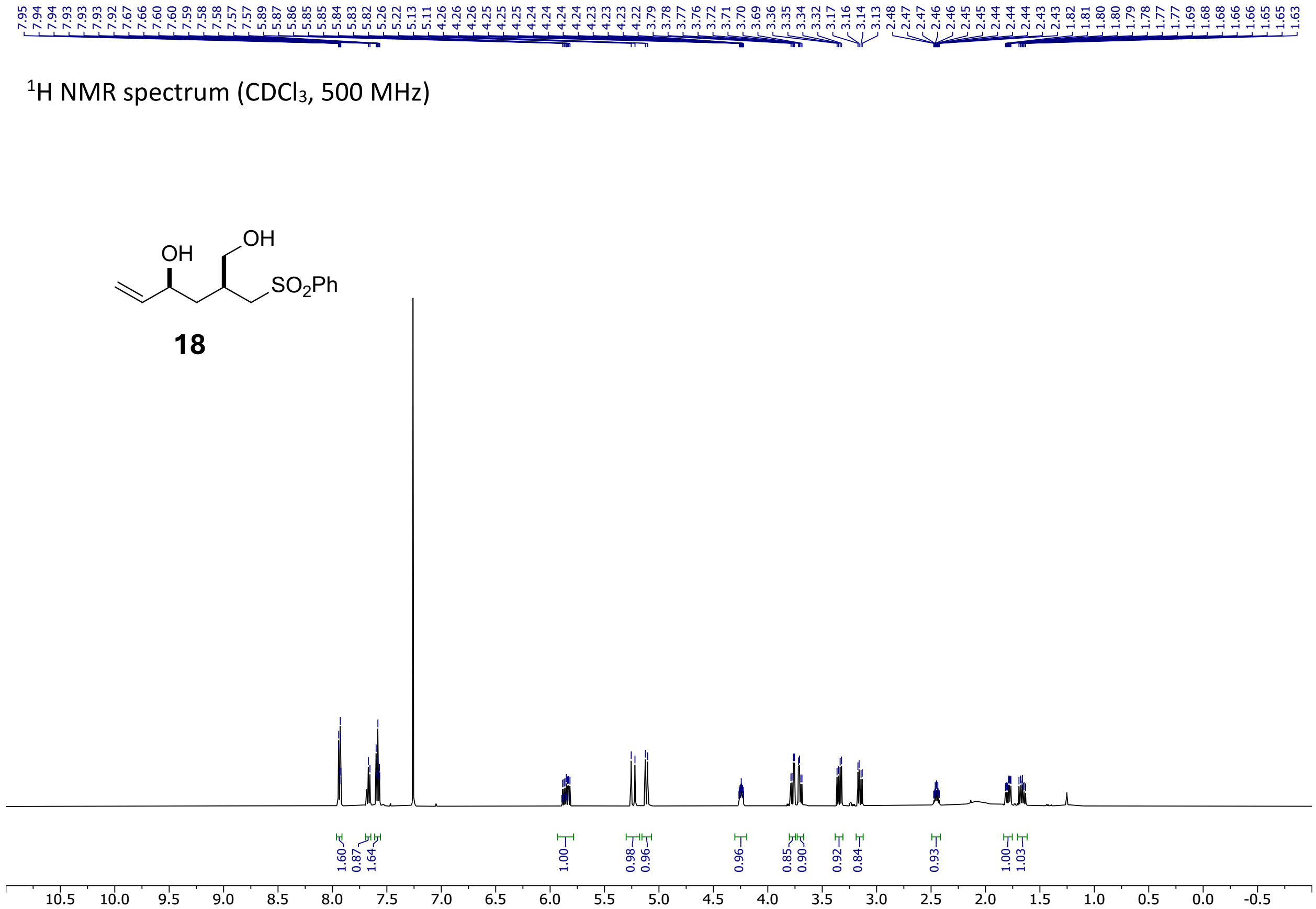

^{1}H NMR spectrum (CDCl$_3$, 500 MHz)

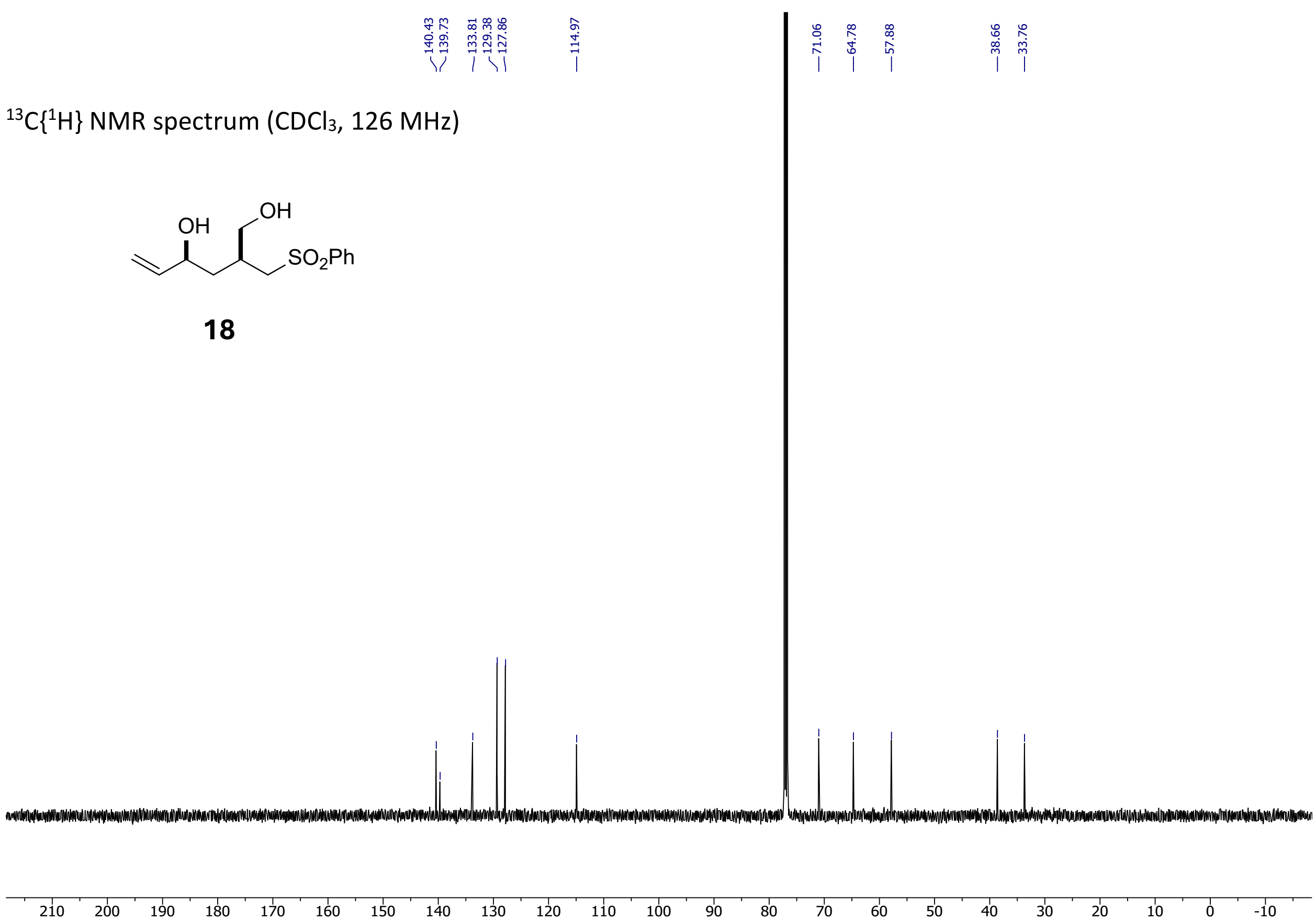

^{13}C{^{1}H} NMR spectrum (CDCl$_3$, 126 MHz)

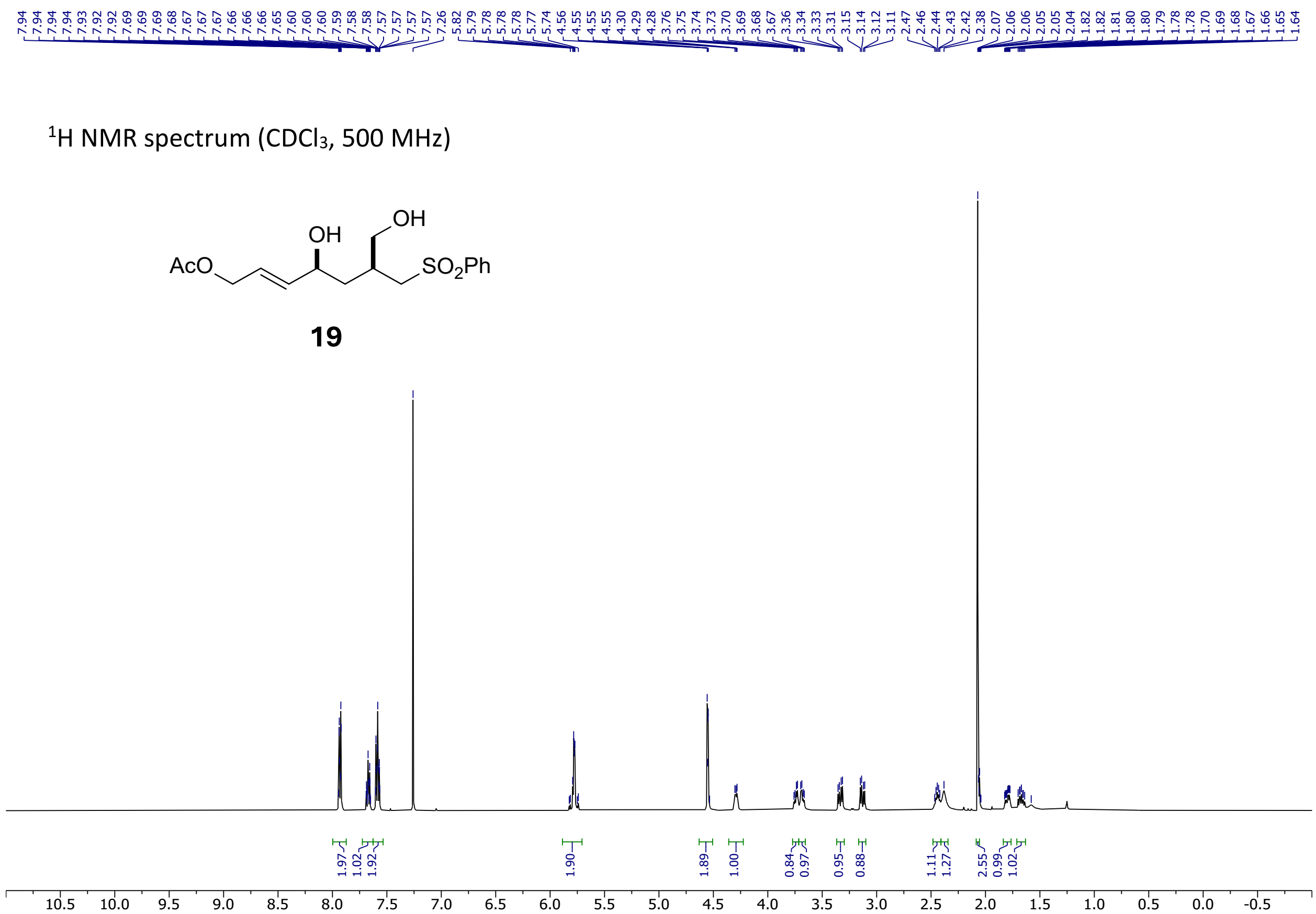

^{1}H NMR spectrum ($CDCl_3$, 500 MHz)
OH
OH
AcO
SO2Ph
19

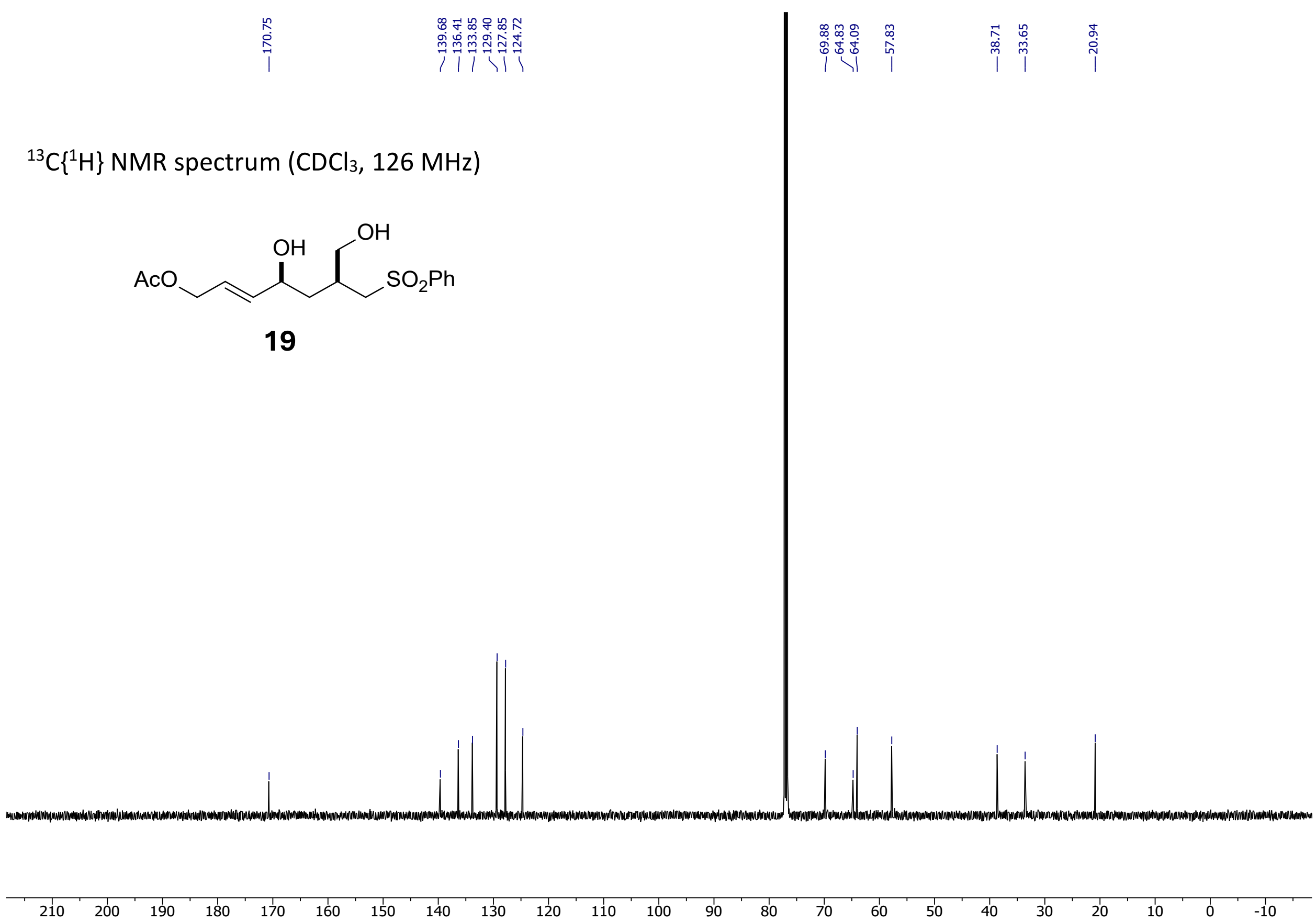

170.75
139.68
136.41
133.85
129.40
127.85
124.72
69.88
64.83
64.09
57.83
38.71
33.65
20.94
^{13}C{^{1}H} NMR spectrum ($CDCl_3$, 126 MHz)
OH
OH
AcO
SO2Ph
19

Figure S6. Summary of computational results.

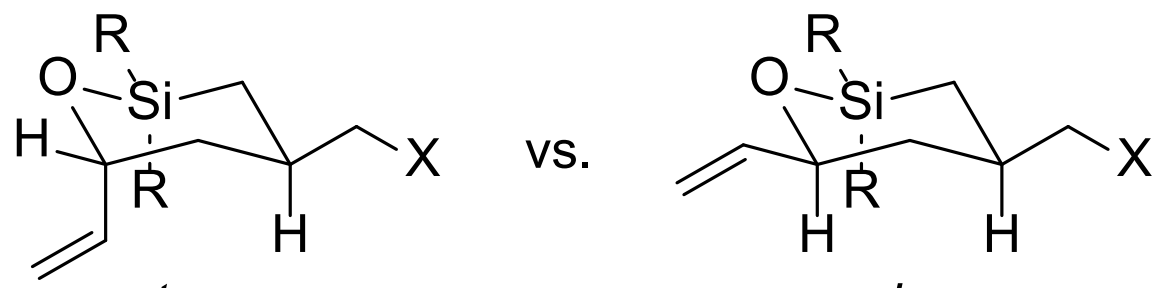

R	X	Conformation	DFT-PBE Energy (Hartree)
Methyl	H	Anti, $-CH_2X$ axial	–718.004221
		**Anti, $-CH_2X$ equatorial**	**–718.005730**
		Syn, $-CH_2X$ axial	–718.000390
		**Syn, $-CH_2X$ equatorial**	**–718.008098**
**$\Delta_{anti-syn}$ = 0.00237 Hartree = 6.22 kJ/mol (computed with DFT-PBE)**			

R	X	Conformation	DFT-PBE Energy (Hartree)
Methyl	I	Anti, $-CH_2X$ axial	–1015.105719
		**Anti, $-CH_2X$ equatorial**	**–1015.107743**
		Syn, $-CH_2X$ axial	–1015.102935
		**Syn, $-CH_2X$ equatorial**	**–1015.110082**
**$\Delta_{anti-syn}$ = 0.00234 Hartree = 6.14 kJ/mol (computed with DFT-PBE)**			

R	X	Conformation	DFT-PBE Energy (Hartree)
Phenyl	H	Anti, $-CH_2X$ axial	–1101.118580
		**Anti, $-CH_2X$ equatorial**	**–1101.119019**
		Syn, $-CH_2X$ axial	–1101.113217
		**Syn, $-CH_2X$ equatorial**	**–1101.122011**
**$\Delta_{anti-syn}$ = 0.00299 Hartree = 7.86 kJ/mol (computed with DFT-PBE)**			

R	X	Conformation	DFT-PBE Energy (Hartree)
Phenyl	I	Anti, $-CH_2X$ axial	–1398.220521
		**Anti, $-CH_2X$ equatorial**	**–1398.220937**
		Syn, $-CH_2X$ axial	–1398.215886
		**Syn, $-CH_2X$ equatorial**	**–1398.223962**
**$\Delta_{anti-syn}$ = 0.00302 Hartree = 7.94 kJ/mol (computed with DFT-PBE)**			

R	X	Conformation	DFT-B3LYP Energy (Hartree)
Methyl	H	Anti, –CH_2X axial	–718.421490
		**Anti, –CH_2X equatorial**	**–718.422715**
		Syn, –CH_2X axial	–718.416921
		**Syn, –CH_2X equatorial**	**–718.425617**
**$\Delta_{anti–syn}$ = 0.00290 Hartree = 7.62 kJ/mol (computed with DFT-B3LYP)**			

R	X	Conformation	DFT-B3LYP Energy (Hartree)
Methyl	I	Anti, –CH_2X axial	–1015.528542
		**Anti, –CH_2X equatorial**	**–1015.530250**
		Syn, –CH_2X axial	–1015.524904
		**Syn, –CH_2X equatorial**	**–1015.533194**
**$\Delta_{anti–syn}$ = 0.00294 Hartree = 7.73 kJ/mol (computed with DFT-B3LYP)**			

.xyz files of PBE-DFT-optimized structures

R=Methyl, X=H (axial), anti

29

C 0.00000000 0.00000000 0.00000000

O 1.43246300 0.13156400 -0.05045300

Si 2.16968700 1.63853800 -0.06901700

C 1.13596400 2.69585300 1.11221000

C -0.37350500 2.34593300 1.07988800

C -0.60506100 0.82081400 1.15289100

H -1.68872800 0.62029800 1.17788400

H -0.18674600 0.43164400 2.09738700

H -0.83350700 2.76855100 1.99123000

C -1.09838000 2.98599000 -0.11003500

H -0.71748100 2.62617000 -1.07723000

H -2.17674600 2.76669400 -0.07867100

H -0.97569400 4.07960300 -0.09940100

H 1.27972800 3.77525800 0.93758000

H 1.52536100 2.49649300 2.12651500

C 3.91472200 1.37234700 0.56356300

H 4.47451000 2.31978300 0.58805600

H 3.90534600 0.95328000 1.57973000

H 4.46284200 0.67474600 -0.08702100

C 2.23733000 2.32953400 -1.82116600

H 2.69555900 3.33065600 -1.82486400

H 2.84585700 1.67876900 -2.46692400

H 1.24070800 2.41917300 -2.27579500

H -0.43432300 0.34146500 -0.95992100

C -0.31485200 -1.45969900 0.16425100

C -1.11490900 -2.15958900 -0.64189700

H -1.33300400 -3.21336800 -0.46182500

H -1.58466500 -1.70087600 -1.51594000

H 0.15945900 -1.93882100 1.02797500

R=Methyl, X=H (equatorial), anti

29

C 0.00000000 0.00000000 0.00000000

O 1.39197100 0.23928300 -0.18541700

Si 2.17125800 1.71967400 -0.06872200

C 1.00368400 2.86481500 0.87907400

C -0.47250200 2.45926700 0.67988600

C -0.65828600 0.96850200 1.00567400

H -1.73234300 0.72277500 1.05020600

H -0.24818000 0.76612600 2.01080500

C -1.41057200 3.32581300 1.52420200

H -2.46734000 3.07096500 1.34516800

H -1.20843500 3.18743000 2.59943700

H -1.27523400 4.39428600 1.29603000

H -0.73921000 2.60621700 -0.38376600

H 1.16052900 3.91413000 0.57668700

H 1.24090000 2.81735700 1.95753400

C 3.78033700 1.39579400 0.84288400

H 4.37173800 2.31853700 0.94669200

H 3.59086900 0.99599000 1.84936300

H 4.39386200 0.66294500 0.29742300

C 2.55407400 2.41636000 -1.77599100

H 3.16048600 3.33129500 -1.68406500

H 3.12646500 1.69623100 -2.38056400

H 1.63794200 2.67156000 -2.32854600

C -0.75824300 -0.05175900 -1.30412900

C -0.23303800 0.06090400 -2.52548000

H -0.85475300 -0.01196600 -3.41852600

H 0.83698500 0.21772500 -2.66617100

H -1.83459500 -0.22944900 -1.19148400

H -0.07581700 -1.01344400 0.44431600

R=Methyl, X=H (axial), syn

29

C 0.00000000 0.00000000 0.00000000

O 1.41138600 0.18894700 -0.05236100

Si 2.21634500 1.65966200 -0.00782500

C	1.03052400	2.88196500	0.81547500
C	-0.46118900	2.54145800	0.57100800
C	-0.73295500	1.05191600	0.86491100
H	-1.81521800	0.85145500	0.78682100
H	-0.45719600	0.85819700	1.91536200
H	-1.05419000	3.10396700	1.31466600
C	-0.94824600	2.99579300	-0.80988300
H	-0.40902300	2.49842200	-1.62764400
H	-2.01828600	2.77367700	-0.94433700
H	-0.81192100	4.08197000	-0.92682700
H	1.24181300	3.91736900	0.50050300
H	1.23125000	2.84655800	1.90078100
C	3.75722500	1.37177200	1.02551700
H	4.36228700	2.28868300	1.10048000
H	3.49847900	1.04859500	2.04396900
H	4.38654000	0.59097100	0.57220300
C	2.73532000	2.20195500	-1.73492300
H	3.30350100	3.14436600	-1.68235300
H	3.38433000	1.44731900	-2.20539400
H	1.87040300	2.36390000	-2.39331600
C	-0.60477100	-0.20956600	-1.36782800
C	0.07122300	-0.36543300	-2.50814600
H	-0.44370000	-0.55799900	-3.44984700
H	1.16074500	-0.30775600	-2.52951900
H	-1.69841200	-0.29242000	-1.37096900
H	-0.14962400	-0.96354100	0.53145600

R=Methyl, X=H (equatorial), syn

29

Si	0.00000000	0.00000000	0.00000000
O	1.67632800	0.05557800	0.04664400
C	2.36945600	1.31510300	0.01639900
C	1.90804000	2.19553200	-1.15751500
C	0.41448600	2.56819800	-1.12929500
C	-0.48652800	1.31962000	-1.25953400
H	-1.54738200	1.60525600	-1.16339500
H	-0.36945300	0.89689500	-2.27443600
H	0.21398400	3.03854700	-0.14698000
C	0.09558400	3.60415000	-2.21149100
H	0.30535700	3.19469700	-3.21267900
H	-0.96646800	3.88939400	-2.18501100
H	0.69760700	4.51737000	-2.08874800
H	2.51338800	3.11618200	-1.14308800
H	2.14169400	1.67539300	-2.10458700
H	2.17506300	1.86422600	0.96103500
C	3.83978200	1.01870800	-0.06529200
C	4.75724700	1.49078200	0.78098000
H	4.47825000	2.12562400	1.62643600
H	5.81780500	1.26181400	0.66521400
H	4.13363400	0.36909400	-0.89727100
C	-0.71900500	0.38861400	1.69767500
H	-1.81829800	0.32417900	1.68287700
H	-0.34926300	-0.32366500	2.45002400
H	-0.45039000	1.40092500	2.03470700
C	-0.44960300	-1.74229100	-0.52201900
H	-1.54155700	-1.86792900	-0.57741200
H	-0.02701900	-1.97738900	-1.50908300
H	-0.06101400	-2.47816200	0.19692400

R=Methyl, X=I (axial), anti

29

C 0.00000000 0.00000000 0.00000000

O 1.43048500 0.13383800 -0.02059300

Si 2.15775300 1.64731000 -0.05596200

C 1.11046300 2.70696100 1.11152400

C -0.39552500 2.36177100 1.06873600

C -0.63295300 0.83041500 1.13251700

H -1.71595700 0.62649700 1.14191800

H -0.23073400 0.45737300 2.09001800

H -0.87595400 2.78418800 1.96827500

C -1.13045600 2.94654000 -0.13388500

H -0.66485200 2.70959300 -1.09663900

H -2.18296500 2.64242800 -0.15511100

I -1.21902200 5.12398600 -0.12078500

H 1.25389500 3.78664800 0.94104700

H 1.48207800 2.50818000 2.13171900

C 3.90157300 1.40838900 0.58522200

H 4.45260400 2.36121400 0.59106900

H 3.89506700 1.00935100 1.60935200

H 4.45578300 0.70338800 -0.05200400

C 2.21510900 2.31752400 -1.81596000

H 2.61505200 3.34307400 -1.82737500

H 2.87028600 1.69203200 -2.44067700

H 1.22630000 2.34506000 -2.29622200

H -0.41661700 0.32727100 -0.97345500

C -0.31951100 -1.45753800 0.17675400

C -1.09728200 -2.16831300 -0.64130500

H -1.31830100 -3.22007800 -0.45362600

H -1.54337300 -1.72247200 -1.53417700

H 0.13282500 -1.92428200 1.05879100

R=Methyl, X=I (equatorial), anti

29

C 0.00000000 0.00000000 0.00000000

O 1.38850800 0.23966800 -0.18536600

Si 2.16857500 1.72210700 -0.07538600

C 0.99689600 2.86609400 0.87023500

C -0.47224700 2.46036600 0.65946200

C -0.66055100 0.96845200 1.00889800

H -1.73240900 0.71483000 1.05541200

H -0.24282200 0.77406800 2.01138500

C -1.44803000 3.27645500 1.49926300

H -2.48759700 2.95799400 1.35536700

H -1.19977800 3.25015400 2.56809000

I -1.47785300 5.39769000 1.00024300

H -0.74308900 2.59588100 -0.40331100

H 1.15300000 3.91460600 0.56745100

H 1.22899700 2.81636100 1.94949700

C 3.77281400 1.40868900 0.84514700

H 4.35682600 2.33573100 0.95141200

H 3.58152100 1.00760500 1.85070200

H 4.39438100 0.68055300 0.30261200

C 2.54778600 2.41607500 -1.78223500

H 3.15173700 3.33246200 -1.68992500

H 3.12232900 1.69684300 -2.38573100

H 1.63207100 2.67069600 -2.33558500

C -0.76344300 -0.05275600 -1.30100800

C -0.24048700 0.06291900 -2.52282500

H -0.86351000 -0.01053400 -3.41474400

H 0.82877100 0.22285000 -2.66524800

H -1.83876400 -0.23474800 -1.18610200

H -0.07798000 -1.01039600 0.45082700

R=Methyl, X=I (axial), syn

29

C 0.00000000 0.00000000 0.00000000

O 1.40546400 0.20353100 -0.06913300

Si 2.21365700 1.67339100 -0.00876700

C 1.01037600 2.90250100 0.77892100

C -0.47424700 2.55256600 0.53037100

C -0.74058700 1.05936000 0.84865500

H -1.82133700 0.84972600 0.78219100

H -0.45702000 0.90030300 1.90236300

H -1.08846400 3.12674200 1.24541000

C -0.97399700 2.91248400 -0.86427000

H -0.36214100 2.50550000 -1.67491100

H -2.01562600 2.60872700 -1.01764800

I -1.00540800 5.06354000 -1.23713900

H 1.21542900 3.93382600 0.44883300

H 1.19093100 2.88990400 1.86724600

C 3.72762400 1.39599200 1.06406400

H 4.32501800 2.31675800 1.15099700

H 3.44541300 1.07476200 2.07684200

H 4.37311800 0.61772200 0.62955200

C 2.77204600 2.20496700 -1.72539500

H 3.36115100 3.13322100 -1.65636200

H 3.41298500 1.43655300 -2.18445600

H 1.92982800 2.39543500 -2.40552800

C -0.61727300 -0.22898200 -1.35889500

C 0.04584500 -0.33742700 -2.51234600

H -0.47537700 -0.54280700 -3.44772700

H 1.13111100 -0.22982100 -2.55061600

H -1.70528000 -0.36702000 -1.34379100

H -0.13912900 -0.95573100 0.54811900

R=Methyl, X=I (equatorial), syn

29

Si 0.00000000 0.00000000 0.00000000

O 1.67795100 0.08070100 -0.00484800

C 2.34554400 1.34845800 -0.06386200

C 1.84657100 2.20839300 -1.24230900

C 0.34217700 2.55784600 -1.16598400

C -0.53644900 1.29693500 -1.26412400

H -1.59862300 1.56614400 -1.14658100

H -0.43143100 0.86220200 -2.27525100

H 0.16650000 3.04579400 -0.18921300

C 0.04208400 3.58409000 -2.25365100

H 0.09659500 3.14806300 -3.25898300

I -1.96055800 4.43398800 -2.13325000

H 0.70081600 4.45826300 -2.19401200

H 2.44262400 3.13469300 -1.25206400

H 2.05993200 1.67471500 -2.18558500

H 2.16111700 1.90983600 0.87526900

C 3.81976200 1.08224000 -0.17782600

C 4.74594400 1.59137200 0.63663100

H 4.47327600 2.23747000 1.47556800

H 5.80797400 1.38149500 0.50069400

H 4.10656800 0.42078200 -1.00271500

C -0.67570000 0.40206100 1.70974300

H -1.77390700 0.32330100 1.72409600

H -0.27686200 -0.29690700 2.45945600

H -0.41349100 1.42162200 2.02885000

C -0.44242100 -1.74955000 -0.49725000

H -1.53393500 -1.88614700 -0.52565900

H -0.04057100 -1.98980400 -1.49160600

H -0.02963600 -2.47419500 0.21938200

R=Phenyl, X=H (axial), anti

43

Si 0.00000000 0.00000000 0.00000000

O 1.67328700 0.00215400 0.05359600

C 2.41805300 1.23551100 -0.04530000

C 1.98670200 2.05835400 -1.27045200

C 0.51106900 2.51346100 -1.27937000

C -0.45397900 1.30218500 -1.29401700

H -1.49832700 1.63957300 -1.19220400

H -0.39217700 0.79564600 -2.27302500

C 0.19273700 3.49344900 -0.14369500

H 0.88298900 4.35057500 -0.15433400

H -0.82996400 3.88511800 -0.24840800

H 0.25449300 3.02230800 0.84828100

H 0.36949600 3.06459600 -2.22661700

H 2.64066900 2.94314900 -1.32971000

H 2.18693000 1.45303200 -2.17201400

H 2.25366100 1.83064100 0.87338400

C	3.87340400	0.87268600	-0.11850400
C	4.81403100	1.34616000	0.70131000
H	5.86345300	1.06807500	0.59279300
H	4.56620400	2.03352000	1.51496500
H	4.13633600	0.17236900	-0.91928900
C	-0.70205300	0.37833500	1.71045700
C	0.07623000	0.13951400	2.85862000
C	-0.42966800	0.38325900	4.13765000
C	-1.73104000	0.86665200	4.29518700
C	-2.52245900	1.10784300	3.16939800
C	-2.00919500	0.86771600	1.89260500
H	-2.64152200	1.07031700	1.02347900
H	-3.53912400	1.48792200	3.28656900
H	-2.12751700	1.05741200	5.29430900
H	0.19238200	0.19370700	5.01481200
H	1.09474400	-0.24045300	2.74627800
C	-0.50094300	-1.73207700	-0.53504500
C	0.16920000	-2.36441900	-1.59978800
C	-0.21016100	-3.63384700	-2.03904900
C	-1.27474900	-4.29783000	-1.42255600
C	-1.95055100	-3.68936100	-0.36295200
C	-1.56357200	-2.42041200	0.07624300
H	-2.09258900	-1.96242500	0.91564900
H	-2.77800100	-4.20630700	0.12704600
H	-1.57263100	-5.29032400	-1.76513900
H	0.32653600	-4.10856900	-2.86299200
H	1.01050300	-1.86181800	-2.08366700

R=Phenyl, X=H (equatorial), anti

43

Si	0.00000000	0.00000000	0.00000000
O	1.67311900	0.00677600	-0.01035300
C	2.47015200	1.20870200	-0.07488100
C	1.89937100	2.23366600	-1.07376100
C	0.44424700	2.67221700	-0.83185000
C	-0.52197700	1.48546200	-1.04113500
H	-1.56062400	1.78904000	-0.83001500
H	-0.50070100	1.18525700	-2.10436300
H	0.35311900	3.01578000	0.21487900
C	0.08064000	3.85186000	-1.73821800
H	0.17563000	3.57091800	-2.79965900
H	-0.95836100	4.17239200	-1.57103600
H	0.73724100	4.71718700	-1.55837500
H	2.56185600	3.11435100	-1.05832000
H	1.96973200	1.80040300	-2.08785700
C	2.67200400	1.77991900	1.30383100
C	3.86843900	1.93994800	1.87382200
H	4.78735900	1.64624400	1.35909900
H	3.97780300	2.36768900	2.87132000
H	1.76854200	2.06693900	1.85158000
H	3.45584700	0.88681500	-0.45261400
C	-0.67271100	0.12824600	1.76049500
C	0.05548000	-0.41848200	2.83486500
C	-0.43793900	-0.37382400	4.14014400
C	-1.67829200	0.21593400	4.39965900
C	-2.41836400	0.76366100	3.34978600
C	-1.91666100	0.72089600	2.04578000
H	-2.51004200	1.16018700	1.23949400
H	-3.38700500	1.22698400	3.54692400
H	-2.06655300	0.25008500	5.41962000

H 0.14600900 -0.80015200 4.95822800

H 1.02932800 -0.87642000 2.64544400

C -0.49745900 -1.64908600 -0.75389900

C 0.29274100 -2.24477500 -1.75457000

C -0.09306700 -3.44266100 -2.35910600

C -1.28250200 -4.06842600 -1.97659600

C -2.07887400 -3.49508600 -0.98274700

C -1.68577100 -2.29977900 -0.37631900

H -2.30927200 -1.87370400 0.41443000

H -3.00468300 -3.98389200 -0.67331500

H -1.58459800 -5.00551500 -2.44770400

H 0.53748600 -3.89225700 -3.12919700

H 1.23112700 -1.77085000 -2.05197300

R=Phenyl, X=H (axial), syn

43

Si 0.00000000 0.00000000 0.00000000

O 1.67105500 0.05574800 -0.00067600

C 2.45573100 1.26471200 -0.05451500

C 1.84572800 2.32700500 -0.99715500

C 0.36196200 2.71711500 -0.82378800

C -0.56346400 1.49011000 -1.01609400

H -1.61120500 1.76030300 -0.80557300

H -0.53811000 1.17135200 -2.07280000

C 0.04888100 3.47692800 0.47162000

H 0.75504100 4.30525500 0.62902200

H -0.96537400 3.90169200 0.42570100

H 0.08396100 2.82890600 1.35822700

H 0.15349700 3.41821000 -1.65214800

H	2.47263900	3.23038900	-0.92407800
H	1.96875200	1.94347400	-2.02523200
C	2.76287500	1.75062300	1.33879300
C	4.00276300	1.94760600	1.79358400
H	4.87679900	1.75527200	1.16505900
H	4.19422400	2.30857700	2.80501700
H	1.90987300	1.93205700	1.99849200
H	3.41793900	0.96624800	-0.50747700
C	-0.67601600	0.01395000	1.76398700
C	0.09704400	-0.50375200	2.82097000
C	-0.39404200	-0.53662000	4.12785800
C	-1.67729100	-0.05827100	4.40512500
C	-2.46442500	0.45402700	3.37118500
C	-1.96519500	0.49037700	2.06641900
H	-2.59462600	0.90410000	1.27388600
H	-3.46751900	0.82941100	3.58280900
H	-2.06315200	-0.08360900	5.42621000
H	0.22571700	-0.93701100	4.93267700
H	1.10331100	-0.87714600	2.61596500
C	-0.45084800	-1.62791400	-0.82960600
C	0.32759200	-2.11858500	-1.89438900
C	-0.01916400	-3.29596800	-2.55890700
C	-1.15782100	-4.00747100	-2.17219900
C	-1.94159600	-3.53962000	-1.11539000
C	-1.58735400	-2.36350800	-0.45019600
H	-2.20104500	-2.02015900	0.38715900
H	-2.82761400	-4.09606700	-0.80308000
H	-1.43015100	-4.92913300	-2.68983100
H	0.60186800	-3.66257300	-3.37900300
H	1.22797300	-1.57748700	-2.19661700

R=Phenyl, X=H (equatorial), syn

43

Si	0.00000000	0.00000000	0.00000000
O	1.67480100	0.00591400	0.01770800
C	2.38668800	1.26097600	-0.03129000
C	1.93290700	2.12818600	-1.21704500
C	0.44684500	2.53056000	-1.18424900
C	-0.47707900	1.29656800	-1.28407800
H	-1.53391500	1.59824600	-1.19788100
H	-0.37035100	0.83720000	-2.28358900
H	0.26212400	3.02106500	-0.20943300
C	0.13831500	3.54850500	-2.28625800
H	0.33181400	3.11519800	-3.28069300
H	-0.91779600	3.85466700	-2.25692500
H	0.75812300	4.45256600	-2.18635000
H	2.55599300	3.03702300	-1.22179300
H	2.15031100	1.58840400	-2.15700400
H	2.19491100	1.82161600	0.90584800
C	3.85176300	0.94245000	-0.10808900
C	4.77247800	1.40557500	0.73968100
H	5.83021100	1.16199200	0.62813800
H	4.49903700	2.04813400	1.58111500
H	4.14076000	0.28653100	-0.93694000
C	-0.66329000	0.45196500	1.70858000
C	0.07927900	0.10750700	2.85440400
C	-0.39007900	0.40259400	4.13588600
C	-1.61926100	1.04692000	4.29933200
C	-2.37316700	1.39732400	3.17725300
C	-1.89560000	1.10438100	1.89709700

H -2.49796800 1.39491800 1.03185600

H -3.33283600 1.90336200 3.29922000

H -1.98805800 1.27753600 5.30058400

H 0.20394300 0.12936600 5.01030000

H 1.04322600 -0.39450700 2.73787400

C -0.49869700 -1.75023200 -0.46533900

C 0.30380100 -2.51957200 -1.32845800

C -0.08227000 -3.80460900 -1.71484200

C -1.28395700 -4.34462700 -1.24889500

C -2.09241400 -3.59809400 -0.38887500

C -1.69911300 -2.31564200 0.00100000

H -2.33306700 -1.75065700 0.68998100

H -3.02805000 -4.01810000 -0.01510100

H -1.58576700 -5.34914500 -1.55077100

H 0.55826800 -4.38895700 -2.37911300

H 1.25050300 -2.11161600 -1.68889900

R=Phenyl, X=I (axial), anti

43

Si 0.00000000 0.00000000 0.00000000

O 1.67693800 0.00969100 0.02341400

C 2.38174900 1.26723300 0.02976200

C 1.96139800 2.15260200 -1.15704000

C 0.46691800 2.56507000 -1.20065700

C -0.46733300 1.33511900 -1.25584000

H -1.51888500 1.65242800 -1.16871700

H -0.37721100 0.86081400 -2.24827800

C 0.15338700 3.51240000 -0.04531500

H 0.88358200 4.32604800 0.02917500

I -1.74861700 4.54910800 -0.28641000

H 0.05746400 3.01093200 0.92410600

H 0.33845100 3.14113800 -2.13325100

H 2.58906000 3.05788600 -1.14973100

H 2.19932900 1.60881600 -2.08768800

H 2.17224400 1.79850100 0.97969300

C 3.85110000 0.96021600 -0.02187700

C 4.74972300 1.41257000 0.85478200

H 5.81093800 1.17666100 0.76242600

H 4.45423000 2.03704900 1.70229800

H 4.16072300 0.32106200 -0.85628100

C -0.65923700 0.34316800 1.73374200

C 0.11395300 -0.02898500 2.85109500

C -0.35178500 0.17458200 4.15162900

C -1.60714800 0.75171900 4.36208700

C -2.39096000 1.12738900 3.26871600

C -1.91885700 0.92602200 1.96895600

H -2.54309300 1.24060700 1.12835900

H -3.36950800 1.58409300 3.42741200

H -1.97307200 0.91156300 5.37818500

H 0.26519400 -0.11784000 5.00358100

H 1.09739600 -0.48019100 2.69665300

C -0.50617400 -1.71609100 -0.56893500

C 0.18412200 -2.35292800 -1.61797600

C -0.20815600 -3.61109800 -2.07832000

C -1.30495400 -4.25684800 -1.50005800

C -2.00098800 -3.64267400 -0.45676700

C -1.60141800 -2.38583200 0.00479800

H -2.14583700 -1.92294900 0.83161300

H -2.85417400 -4.14546100 0.00252500

H -1.61264600 -5.24010700 -1.85995600

H 0.34385200 -4.09132000 -2.88880100

H 1.04982800 -1.86398500 -2.07131800

R=Phenyl, X=I (equatorial), anti

43

Si 0.00000000 0.00000000 0.00000000

O 1.67526700 0.01955600 -0.03379400

C 2.45432300 1.22964500 -0.06822100

C 1.87493500 2.27142300 -1.05021500

C 0.40936200 2.68250900 -0.78287600

C -0.53425000 1.49112000 -1.02764800

H -1.57837500 1.77756300 -0.82547100

H -0.49127800 1.20629300 -2.09442200

H 0.32145600 3.00628000 0.26914300

C 0.09184900 3.88696200 -1.66265500

H 0.06635300 3.62705000 -2.72849400

I -1.85999800 4.77174800 -1.26941300

H 0.79228600 4.71493700 -1.50105500

H 2.53119600 3.15582600 -1.01363000

H 1.94555400 1.85695400 -2.07113100

C 2.64959200 1.77760600 1.32092900

C 3.84598800 1.96154900 1.88349000

H 4.76869300 1.70739700 1.35471600

H 3.95126900 2.36969800 2.88945400

H 1.74245200 2.02207000 1.88301000

H 3.44443100 0.93430300 -0.45634400

C -0.65619000 0.12521500 1.76454800

C 0.04532700 -0.49063800 2.81956700

C -0.43629000 -0.44862100 4.12909200

C -1.63788500 0.20766000 4.41172800

C -2.34970700 0.82519700 3.38148300

C -1.86020600 0.78536100 2.07254600

H -2.42989700 1.28254400 1.28330800

H -3.28684200 1.34188800 3.59699500

H -2.01717900 0.23952400 5.43508900

H 0.12592600 -0.92910800 4.93217600

H 0.98928700 -1.00134500 2.61323700

C -0.49857300 -1.64257400 -0.76129400

C 0.29131900 -2.24439200 -1.75843400

C -0.10265300 -3.43895900 -2.36444200

C -1.29945200 -4.05378500 -1.98714000

C -2.09558600 -3.47357500 -0.99692900

C -1.69469900 -2.28202900 -0.38851600

H -2.31815500 -1.84964900 0.39879400

H -3.02743400 -3.95371100 -0.69246100

H -1.60786800 -4.98798100 -2.45979500

H 0.52699300 -3.89416000 -3.13188500

H 1.23488300 -1.77846900 -2.05179300

R=Phenyl, X=I (axial), syn

43

Si 0.00000000 0.00000000 0.00000000

O 1.67392000 0.05746600 -0.03211800

C 2.43506300 1.27917800 -0.01122200

C 1.83880200 2.35980400 -0.94056700

C 0.34115300 2.73490500 -0.79410800

C -0.56989900 1.50294200 -0.99579500

H -1.62107500 1.76980400 -0.80187100

H -0.52964800 1.19765700 -2.05543800

C 0.06404800 3.48502100 0.50544700

H 0.80231900 4.27023400 0.69930700

I -1.83881500 4.55588700 0.46750600

H -0.02790100 2.83686700 1.38337000

H 0.14349900 3.44540100 -1.61520900

H 2.45762000 3.26588900 -0.84114100

H 1.97236000 1.99920200 -1.97498400

C 2.67566500 1.72828200 1.40710100

C 3.88631000 1.98425300 1.90888600

H 4.79019000 1.86984000 1.30418700

H 4.02256400 2.31709100 2.93856400

H 1.79404400 1.82585000 2.04830300

H 3.41977800 1.01971300 -0.43864800

C -0.64527300 0.00137900 1.77432500

C 0.07339700 -0.68159900 2.77562300

C -0.39455700 -0.73907200 4.08930100

C -1.59997100 -0.11852300 4.43022400

C -2.32954900 0.56265500 3.45408700

C -1.85430400 0.62147000 2.14049400

H -2.43707000 1.17169300 1.39684500

H -3.26930800 1.05276300 3.71515300

H -1.96795500 -0.16391400 5.45713200

H 0.18132400 -1.26980600 4.85012800

H 1.02016300 -1.16582700 2.52307100

C -0.46248300 -1.61616500 -0.83948600

C 0.32032300 -2.12429800 -1.89264100

C -0.04438900 -3.29405300 -2.56119000

C -1.20488500 -3.97828200 -2.19066000

C -1.99355700 -3.49171500 -1.14578800

C -1.62177500 -2.32406800 -0.47580400

H -2.23997500 -1.96507200 0.35168400

H -2.89731100 -4.02645800 -0.84720300

H -1.49117000 -4.89357600 -2.71197300

H 0.57949300 -3.67573600 -3.37213500

H 1.23636600 -1.60330200 -2.18210100

R=Phenyl, X=I (equatorial), syn

43

Si 0.00000000 0.00000000 0.00000000

O 1.67744500 0.01363600 -0.00324500

C 2.37346800 1.27278900 -0.05066900

C 1.91652800 2.13606400 -1.24317400

C 0.41924400 2.52125300 -1.19949200

C -0.48383200 1.27821900 -1.30052500

H -1.54490500 1.56642500 -1.23146200

H -0.35473400 0.80860600 -2.29268400

H 0.23601600 3.02246800 -0.23137900

C 0.16100700 3.53795700 -2.30647900

H 0.22366600 3.08800400 -3.30525500

I -1.82581700 4.42949900 -2.23208300

H 0.83655200 4.39918700 -2.24543200

H 2.53386400 3.04850300 -1.24481600

H 2.13729100 1.59354900 -2.17975200

H 2.17368900 1.83626500 0.88286700

C 3.84330900 0.97553400 -0.12821000

C 4.75649800 1.45848000 0.71648600

H 5.81740200 1.22792400 0.60792900

H 4.47348400 2.10323300 1.55296900

H 4.14069500 0.31662400 -0.95155300

C	-0.64877600	0.47898300	1.70408200
C	0.02286700	0.01429600	2.85178700
C	-0.43356600	0.32830500	4.13298000
C	-1.57890700	1.11296500	4.29365500
C	-2.26026700	1.58456500	3.16996000
C	-1.79612600	1.27188400	1.88929000
H	-2.34077000	1.65916500	1.02419900
H	-3.15354300	2.20072500	3.29035700
H	-1.93859000	1.35822500	5.29466900
H	0.10440300	-0.03945300	5.00883300
H	0.92193800	-0.59750300	2.73827200
C	-0.49556100	-1.75400900	-0.44431100
C	0.32262400	-2.54895900	-1.26844700
C	-0.06582100	-3.83873300	-1.63643700
C	-1.28481000	-4.35670800	-1.19093400
C	-2.10906200	-3.58385200	-0.36979500
C	-1.71390200	-2.29688300	0.00235800
H	-2.36100100	-1.70994500	0.66005300
H	-3.05878400	-3.98651500	-0.01311000
H	-1.58864700	-5.36467700	-1.47888900
H	0.58616600	-4.44378100	-2.27023800
H	1.28185700	-2.15696500	-1.61283200
